# Truncated yet functional viral protein produced *via* RNA polymerase slippage implies underestimated coding capacity of RNA viruses

**DOI:** 10.1038/srep21411

**Published:** 2016-02-22

**Authors:** Yuka Hagiwara-Komoda, Sun Hee Choi, Masanao Sato, Go Atsumi, Junya Abe, Junya Fukuda, Mie N. Honjo, Atsushi J. Nagano, Keisuke Komoda, Kenji S. Nakahara, Ichiro Uyeda, Satoshi Naito

**Affiliations:** 1 Research Faculty of Agriculture, Hokkaido University, Sapporo 060-8589, Japan; 2 Graduate School of Agriculture, Hokkaido University, Sapporo 060-8589, Japan; 3 Institute for Advanced Biosciences, Keio University, Tsuruoka 997-0017, Japan; 4 Graduate School of Media and Governance, Keio University, Fujisawa 252-0882, Japan; 5 Iwate Biotechnology Research Center, Kitakami 024-0003, Japan; 6 National Institute of Advanced Industrial Science and Technology, Sapporo 062-8517, Japan; 7 Center for Ecological Research, Kyoto University, Otsu 520-2113, Japan; 8 Japan Science and Technology Agency, PRESTO, Kawaguchi 332-0012, Japan; 9 Faculty of Agriculture, Ryukoku University, Otsu 520-2194, Japan; 10 Faculty of Advanced Life Science, Hokkaido University, Sapporo 060-0810, Japan; 11 Graduate School of Life Science, Hokkaido University, Sapporo 060-0810, Japan

## Abstract

RNA viruses use various strategies to condense their genetic information into small genomes. Potyviruses not only use the polyprotein strategy, but also embed an open reading frame, *pipo*, in the *P3* cistron in the –1 reading frame. PIPO is expressed as a fusion protein with the N-terminal half of P3 (P3N-PIPO) *via* transcriptional slippage of viral RNA-dependent RNA polymerase (RdRp). We herein show that clover yellow vein virus (ClYVV) produces a previously unidentified factor, P3N-ALT, in the +1 reading frame *via* transcriptional slippage at a conserved G_1–2_A_6–7_ motif, as is the case for P3N-PIPO. The translation of P3N-ALT terminates soon, and it is considered to be a C-terminal truncated form of P3. *In planta* experiments indicate that P3N-ALT functions in cell-to-cell movement along with P3N-PIPO. Hence, all three reading frames are used to produce functional proteins. Deep sequencing of ClYVV RNA from infected plants endorses the slippage by viral RdRp. Our findings unveil a virus strategy that optimizes the coding capacity.

Viruses that possess single-stranded, mRNA-sense genomes are called positive-strand RNA viruses. While most mRNAs in eukaryotic cells are monocistronic, positive-strand RNA viruses encode multiple proteins in single polycistronic genomes. Several strategies are adopted to translate viral internal genes, such as synthesis of subgenomic RNAs^1^,^2^, translation from an internal ribosome entry site^3^,^4^,^5^, stop codon readthrough^6^ and polyprotein production followed by processing with cellular and/or viral proteases^7^,^8^. In addition, ribosomal frameshift (FS)^9^,^10^,^11^,^12^ and transcriptional slippage (TS)^13^ by the viral RNA-dependent RNA polymerase (RdRp) are also used to express overlapping genes from different reading frames^14^,^15^.

Potyviruses belong to the family *Potyviridae* in the picornavirus-like supergroup of positive-strand RNA viruses, and comprise one of the largest genera of plant viruses. They cause diseases in many crops, resulting in severe economic losses. For example, plum pox virus has spread throughout Europe, Asia, North Africa, and South and North America. The estimated costs associated with sharka disease of stone fruits have exceeded 10 billion Euros in the last 30 years^16^. The potyviral genome is approximately 10 kb and contains a long open reading frame (ORF) encoding a polyprotein of approximately 350 kDa. The polyprotein is cleaved into at least 10 mature proteins^17^ (Fig. 1a). Recently, a short ORF, *pipo*, was discovered within the P3 coding region in all members of the family *Potyviridae*^18^. The *pipo* ORF exists in the –1 (or +2) reading frame relative to the polyprotein ORF. PIPO is expressed *in planta* as a fusion protein with the N-terminal half of P3 (P3N-PIPO; Supplementary Fig. 1a)^18^,^19^. P3N-PIPO was reported to be involved in viral cell-to-cell movement through plasmodesmata^19^,^20^,^21^. Knockout mutations of *pipo* expression without disrupting the P3 amino acid sequence rendered turnip mosaic virus (TuMV) noninfectious to *Nicotiana benthamiana*^18^. Four other viral proteins, namely helper component proteinase (HC-Pro), cylindrical inclusion (CI) protein, coat protein (CP) and genome-linked virus protein (NIa-VPg) (Fig. 1a), have also been suggested to be involved in viral cell-to-cell movement^22^,^23^. However, none of these proteins, including P3N-PIPO, has a transmembrane domain for localization to the plasma membrane and plasmodesmata. Recently, a cation-binding protein, PCaP1, of *Arabidopsis thaliana* was shown to interact with TuMV P3N-PIPO based on yeast two-hybrid analysis^19^. PCaP1 and its *N. benthamiana* homologue, NbDREPP, interact with the PIPO domain (*i.e.*, the PIPO part of P3N-PIPO) to recruit P3N-PIPO to plasmodesmata *in planta*^19^,^24^.

At the 5′ end of the *pipo* ORF, there is a G_1–2_A_6–7_ motif that is highly conserved among potyviruses^18^. It has been suggested that either –1/+2 FS or TS preferentially occurs at or around the G_1–2_A_6–7_ motif^18^. However, this (G)_GAA_AAA_A(A) motif (underlines denote the polyprotein reading frame) differs from the typical X_XXY_YYZ slippery sequence of –1 FS^25^,^26^,^27^. In addition, there is no conserved downstream RNA secondary structure that has been suggested to function as a stimulator of –1 FS^28^,^29^,^30^. Although it is unknown whether FS occurs around the G_1–2_A_6–7_ motif, Rodamilans *et al.*^31^ recently reported that P3N-PIPO of the plum pox virus and sweet potato feathery mottle virus, which are potyviruses, might be expressed, at least partially, through TS. Olspert *et al.*^32^ also demonstrated the involvement of TS in the expression of P3N-PIPO in TuMV and in two other potyviruses.

*Clover yellow vein virus* (ClYVV) belongs to the genus *Potyvirus* in the family *Potyviridae*. It causes severe systemic necrosis in legumes, including French bean, broad bean and pea^33^. Previously, we screened pea accessions for those showing necrotic reactions or resistance to ClYVV, and identified three distinct loci on pea genomes that are responsible for the defence responses^34^,^35^,^36^. Recently, we found that ClYVV P3N-PIPO is quantitatively and qualitatively involved in breaking one of the identified resistances and in developing necrotic symptoms *via* activation of salicylic acid-mediated defence signalling in susceptible pea accessions^37^,^38^. The enhanced virulence of ClYVV was ascribed to the increased accumulation of P3N-PIPO in these pea accessions.

The objective of this study is to investigate the mechanistic details of how ClYVV establishes virulence. To characterize the expression mechanism of P3N-PIPO, the highly virulent ClYVV strain RB^38^, which produces increased levels of P3N-PIPO, enables us to examine whether P3N-PIPO is expressed *via* TS or FS, using an *in vitro* translation system^38^. We herein reveal that the ClYVV *P3* cistron produces another alternative reading frame product, P3N-ALT, whose translation terminates immediately downstream of the G_2_A_6_ motif in the +1 reading frame. Although P3N-ALT is considered to be a C-terminal truncated form of P3, it acts cooperatively with P3N-PIPO in viral cell-to-cell movement. Amplicon sequencing analysis using RNAs obtained from ClYVV-infected broad bean plants indicates that genomes with nucleotide insertions or deletions at A_6_ in the G_2_A_6_ motif are produced during infection. This suggests that TS by the viral RdRp is responsible for the expression of P3N-PIPO and P3N-ALT.

## Results

### P3N-PIPO and P3N-ALT are produced *in vitro*

**Figure 1:**
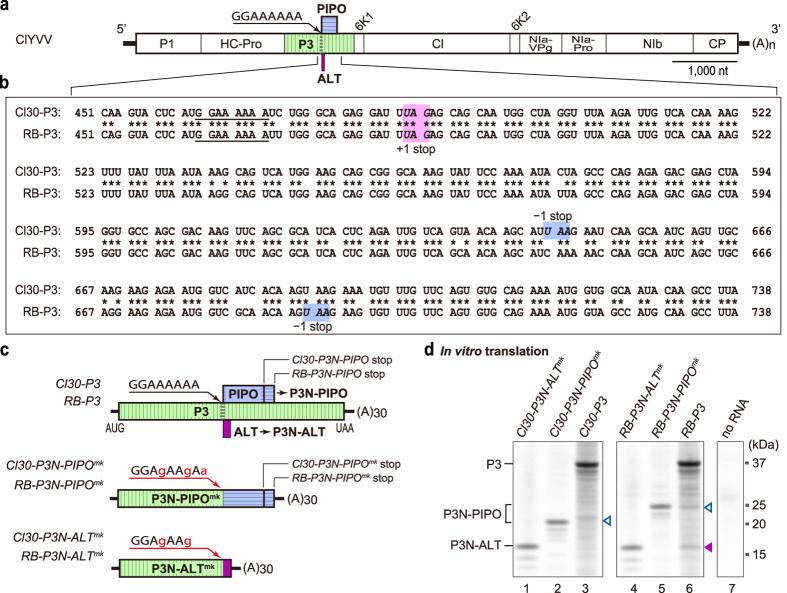
Alternative reading frame proteins encoded in the P3 coding region of ClYVV. (**a**) Schematic diagram of the genomes of two ClYVV variants, Cl30 and RB. The polyprotein expressed from the genome is processed into P1; helper component proteinase (HC-Pro); P3 (green box with vertical stripes); 6K1; cylindrical inclusion (CI); 6K2; nuclear inclusion A (NIa), which is further cleaved into NIa-VPg and proteinase domain (NIa-Pro); nuclear inclusion protein B (NIb); and coat protein (CP). The positions of the *pipo* ORF (blue box with horizontal stripes), the *alt* ORF identified in this study (magenta box), and the conserved G_1-2_A_6-7_ motif are indicated. (**b**) Alignment of the nucleotide sequences of part of the *pipo* ORF of Cl30 and RB. Spaces denote the polyprotein reading frame. The G_2_A_6_ motif is underlined. The stop codons of the *pipo* ORF (–1 stop) and P3N-ALT (+1 stop) are italicized and shaded. The nucleotides are numbered from the start of the *P3* ORF. (**c**) Schematic diagrams of *Cl30-P3* and *RB-P3* RNAs for *in vitro* expression analysis, *Cl30-P3N-PIPO*^*mk*^ and *RB-P3N-PIPO*^*mk*^ RNAs used to prepare size markers for P3N-PIPO, and *Cl30-P3N-ALT*^*mk*^ and *RB-P3N-ALT*^*mk*^ RNAs used to prepare size markers for P3N-ALT. The mutated nucleotides in G_2_A_6_ motif in the RNAs for size marker preparation are indicated by lower case red letters. (**d**) *In vitro* translation analysis using WGE. The radiolabeled translation products were visualized by autoradiography. The positions of P3, P3N-PIPO and P3N-ALT are marked on the left side of the panel. Open and closed arrowheads indicate the P3N-PIPO (–1 reading frame) and P3N-ALT (+1 reading frame), respectively. Positions of molecular mass markers (kDa) are indicated on the right side of the panel. A representative image of triplicated experiments is shown.

To analyse P3N-PIPO expression, we used two isolates of ClYVV, Cl30 and RB^38^ (Fig. 1a). The size of the *pipo* ORF differed between the two isolates (Fig. 1b), with RB expressing a slightly larger P3N-PIPO than Cl30. The accumulated level of P3N-PIPO in RB-infected pea leaves was higher than that in Cl30-infected pea leaves^38^. Our previous study revealed that mRNA carrying the *P3* cistron derived from RB (*RB-P3* mRNA) produced more P3N-PIPO than mRNA carrying that from Cl30 (*Cl30-P3* mRNA) in an *A. thaliana in vitro* translation system^38^. In this study, we first investigated whether *Cl30-P3* and *RB-P3* mRNAs (Fig. 1c) could produce P3N-PIPO in a wheat germ extract (WGE) *in vitro* translation system. The mRNAs were prepared by *in vitro* transcription using bacteriophage SP6 RNA polymerase, and translated in the WGE in the presence of [^35^S]methionine. The translation products were separated by SDS-PAGE and detected by autoradiography. As protein size markers for the P3N-PIPO produced by Cl30 and RB strains, Cl30-P3N-PIPO^mk^ (about 23 kDa) and RB-P3N-PIPO^mk^ (about 25 kDa), respectively, were also synthesized *in vitro*. The *RB-P3* RNA produced a detectable amount of P3N-PIPO, while *Cl30-P3* RNA produced a trace amount of P3N-PIPO in the WGE (Fig. 1d, lanes 6 and 3). Importantly and unexpectedly, we found an additional protein band of about 16 kDa when *RB-P3* mRNA was translated in the WGE (Fig. 1d, lane 6). We hypothesized that this 16-kDa protein was produced from another reading frame, and named it P3N-ALT. A stop codon (UAG) is present 16 nucleotides downstream of the G_2_A_6_ motif in the +1 frame (Fig. 1b, magenta box), and a 16-kDa protein would be produced by shifting the reading frame from that of P3 (zero frame) to the +1 frame at around the G_2_A_6_ motif. Indeed, the size of P3N-ALT matched that of the *in vitro*-synthesized RB-P3N-ALT^mk^ designed as a size marker for the +1-reading frame protein (Fig. 1d, lane 4). These results suggested that two alternative reading frame proteins, P3N-PIPO and P3N-ALT, are produced from the *P3* cistron *in vitro*. Because SP6 RNA polymerase was used to prepare the RNA for the WGE *in vitro* translation experiments, either TS by SP6 RNA polymerase or FS by wheat ribosomes contributed to the production of P3N-PIPO and P3N-ALT *in vitro*. The results prompted us to explore the possibility that P3N-ALT is produced *in planta* during ClYVV infection.

### P3N-ALT is expressed *in planta*

**Figure 2:**
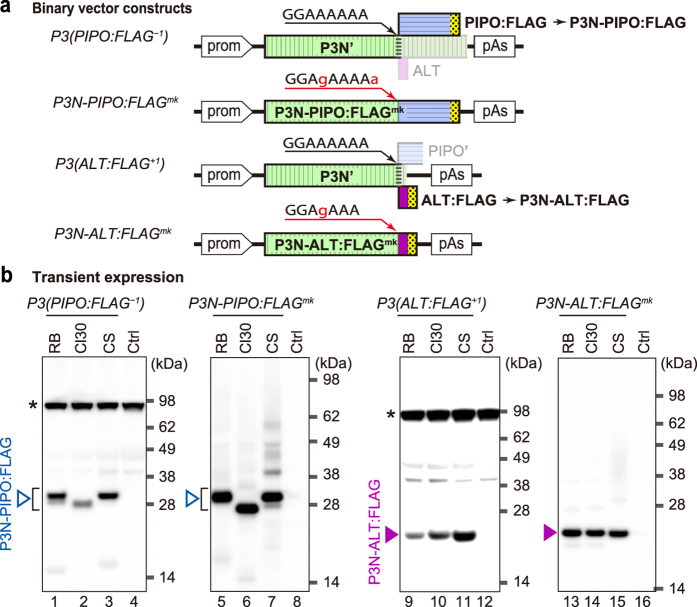
Detection of P3N-PIPO and P3N-ALT products by Agrobacterium-mediated transient expression in *N. benthamiana*. (**a**) Schematic diagrams of the plasmids used. The original or mutated G_2_A_6_ motif is indicated above each diagram with mutated nucleotides in lower case red letters. The *P3* (green box with vertical stripes), *pipo* (blue box with horizontal stripes) and *alt* (magenta box) ORFs from Cl30 and RB strains of ClYVV, and CS strain of BYMV (CS), and the FLAG-tag (yellow box with dots) are marked. These modified *P3* cistrons were inserted in a binary vector between a DEX-inducible promoter (prom) and a poly(A) addition signal (pAs). The binary vectors with *P3*(*PIPO:FLAG*^–1^) express FLAG-tagged P3N-PIPO from the –1 reading frame, and the vectors with *P3*(*ALT:FLAG*^+1^) express FLAG-tagged P3N-ALT from the +1 reading frame. These constructs coincidently express proteins without the FLAG-tag from other frames (shaded). Their expected amino acid sequences are presented in Supplementary Fig. 2. *P3N-PIPO:FLAG*^*mk*^ and *P3N-ALT:FLAG*^*mk*^ RNAs are designed to express FLAG-tagged proteins in-frame, respectively. (**b**) Western blotting using anti-FLAG antibody to detect FLAG-tagged P3N-PIPO as –1 reading frame products after translation of *P3*(*PIPO:FLAG*^–1^) RNA (lanes 1–3) and as in-frame products after translation of *P3N-PIPO:FLAG*^*mk*^ RNA (lanes 5–7). FLAG-tagged P3N-ALT was detected as +1 reading frame products after translation of *P3*(*ALT:FLAG*^+1^) RNA (lanes 9–11) and in-frame products after translation of *P3N-ALT:FLAG*^*mk*^ RNA (lanes 13–15). In the control (Ctrl, lanes 4, 8, 12 and 16), protein samples prepared from leaves that expressed yellow fluorescence by agroinfiltration with the vector carrying the *YFP* gene were analysed as negative controls. Samples in lanes RB, Cl30 and CS were prepared from leaves expressing the P3 derivatives of RB, Cl30 and BYMV-CS, respectively. Open and closed arrowheads indicate the positions of P3N-PIPO:FLAG and P3N-ALT:FLAG, respectively. The asterisks denote background signals derived from non-specific cross-reaction of the anti-FLAG antibody. Positions of molecular mass markers (kDa) are indicated on the right side of each panel. The analysis was repeated four times, and typical images are shown.

We examined whether P3N-ALT is expressed *in planta* as an alternative reading frame product of the *P3* cistron. We prepared binary vectors carrying the modified *P3* cistrons of ClYVV, Cl30 and RB and another potyvirus, bean yellow mosaic virus (BYMV) CS isolate^39^, in which either P3N-PIPO or P3N-ALT was tagged with the FLAG peptide, and placed under the control of a dexamethasone (DEX)-inducible promoter (Fig. 2a). The vectors carrying P3(PIPO:FLAG^–1^) and P3(ALT:FLAG^+1^) express the FLAG-tagged P3N-PIPO proteins as –1 frame products and the FLAG-tagged P3N-ALT proteins as +1 frame products, respectively. P3N-PIPO:FLAG^mk^ and P3N-ALT:FLAG^mk^ were modified by mutations in the G_2_A_6_ motif to produce the FLAG-tagged proteins as zero-frame products, which were used as size markers (Fig. 2a). *Agrobacterium tumefaciens* transformants carrying the binary vectors were infiltrated into *N. benthamiana* leaves, and protein expression was induced with DEX. The proteins expressed in the infiltrated areas were analysed by western blot using an anti-FLAG antibody.

We detected P3N-PIPO:FLAG, which was expressed as a –1 frame product in the infiltrated area (Fig. 2b, lanes 1–3). Their sizes agreed with their respective size markers (Fig. 2b, lanes 5–7), suggesting that in this transient assay, P3N-PIPO was expressed from the *P3* cistron as an alternative frame product. We also detected P3N-ALT:FLAG, which was expressed as a +1 frame product (Fig. 2b, lanes 9–11) whose size corresponded to the respective size markers (Fig. 2b, lanes 13–15).

**Figure 3:**
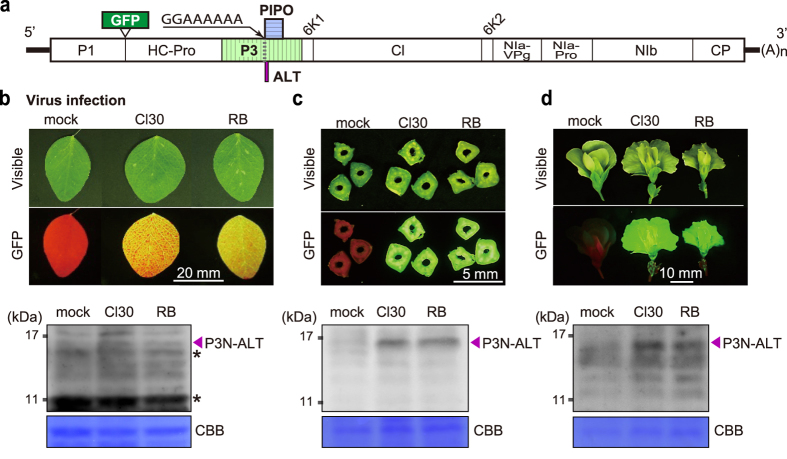
Detection of P3N-ALT accumulated in ClYVV-infected plant tissues. (**a**) Schematic diagram of Cl30 and RB derivatives carrying a GFP gene. (**b**-**d**) Detection of GFP fluorescence and P3N-ALT in systemically infected tissues. Leaf (**b**), stem (**c**) and flower (**d**) tissues were harvested at 5, 10, and 24 days post-inoculation, respectively. The infected areas were confirmed by virus-derived GFP fluorescence (upper panels). P3N-ALT was detected by western blotting (lower panels), using a polyclonal antibody raised against the N-terminal region of P3. Coomassie brilliant blue (CBB)-stained gel images are shown at the bottom of each western blot panel as a loading control. The position of P3N-ALT is marked with an arrowhead. The asterisks denote the background signals derived from non-specific cross-reaction of the antiserum. Positions of molecular mass markers (kDa) are indicated on the left side of each panel. The analysis was repeated three times, and typical images are shown.

To further validate P3N-ALT expression during ClYVV infection, pea plants infected with Cl30 or RB, which were tagged with green fluorescent protein (GFP) (Fig. 3a), were subjected to western blot analysis using antiserum raised against the N-terminal region of P3. The leaf, stem and flower tissues of mechanically inoculated pea plants were harvested at 5, 10 and 24 days post-inoculation. The infected tissue areas were confirmed by virus-derived GFP fluorescence (Fig. 3b–d). In the leaf tissues, only a faint P3N-ALT band was observed (Fig. 3b), whereas in the stem tissue samples, the P3N-ALT band was clearly detected in Cl30- and RB-infected plants (Fig. 3c). P3N-ALT was also detected in the flower tissue samples (Fig. 3d) and in the stem tissues of broad bean plants infected with Cl30 and RB (Supplementary Fig. 3). These results strongly suggested that P3N-ALT, in addition to P3N-PIPO, is produced from the *P3* cistron in ClYVV-infected plants.

### P3N-ALT facilitates the cell-to-cell movement of ClYVV

**Figure 4:**
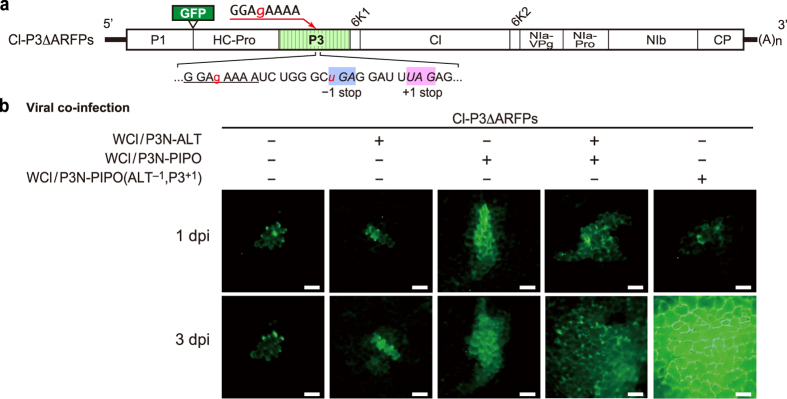
Functional analysis of P3N-ALT supplied *in trans* in ClYVV infection. (**a**) Schematic diagram of Cl-P3ΔARFPs. The G_2_A_6_ motif with the downstream sequence is indicated below each diagram. The mutated nucleotides in Cl-P3ΔARFPs are shown in lower case red letters. The natural stop codon for the *alt* ORF (+1 stop) and the introduced stop codon for the *pipo* ORF (−1 stop) are italicized and shaded. (**b**) Requirement of P3N-ALT and P3N-PIPO for efficient cell-to-cell movement of ClYVV. Pea leaves were biolistically co-inoculated with Cl-P3ΔARFPs and the white clover mosaic virus vectors (WCl) expressing P3N-ALT (WCl/P3N-ALT), P3N-PIPO (WCl/P3N-PIPO), and both P3N-ALT and P3N-PIPO [WCl/P3N-PIPO(ALT^–1^, P3^+1^)]. Cell-to-cell movement was monitored by GFP fluorescence of Cl-P3ΔARFPs. GFP-expressing lesions at 1 or 3 days post-inoculation (dpi) are shown. The biolistic inoculations of 6 leaves with each plasmid or mixture of plasmids were repeated twice, and typical images are shown. Scale bar =50 μm.

We conducted experiments to identify the function of P3N-ALT. It was impossible to knock out the *alt* ORF without affecting the amino acid sequences of P3 and P3N-PIPO; therefore, we designed a mutant of Cl30, termed Cl-P3ΔARFPs, in which alternative reading frame products (ARFPs) are not produced. In Cl-P3ΔARFPs, the G_2_A_6_ motif was mutated to G_2_AGA_4,_ and a TGA stop codon was placed eight nucleotides downstream of the G_2_A_6_ motif in the *pipo* frame, such that it produces a full-length polyprotein from P1 to CP, but not P3N-PIPO or P3N-ALT (Fig. 4a). Propagation of Cl-P3ΔARFPs is predicted to be restricted to single inoculated cells, as P3N-PIPO is required for viral cell-to-cell movement^19^,^20^,^21^. We analysed whether this movement defect of Cl-P3ΔARFPs could be recovered by providing P3N-PIPO and/or P3N-ALT *in trans*. We used white clover mosaic virus (WClMV) vector^40^ to express P3N-ALT in pea plants because no mutual antagonistic effects were observed in a mixed infection of ClYVV and WClMV; both viruses accumulated to levels similar to those observed during single infections^41^. We prepared a WClMV vector series that harboured the *P3* cistron but expressed various reading frame products by mutating the G_2_A_6_ motif: WCl/P3N-PIPO^38^, which expresses P3N-PIPO exclusively; WCl/P3N-ALT, which expresses P3N-ALT exclusively; and WCl/P3N-PIPO(ALT^–1^, P3^+1^)^38^, which expresses P3N-PIPO predominantly and a low amount of P3 and P3N-ALT^41^. These WClMV constructs were biolistically co-inoculated with the Cl-P3ΔARFPs cDNA clone into pea leaf explants, and the cell-to-cell movement of Cl-P3ΔARFPs was analysed by monitoring GFP fluorescence. When Cl-P3ΔARFPs was inoculated alone, the GFP signal was restricted to the initially inoculated single cells (Fig. 4b and Table 1). When co-inoculated with WCl/P3N-ALT, Cl-P3ΔARFPs spread to neighbouring cells, although the movement efficiency was lower than when WCl/P3N-PIPO was used (Fig. 4b and Table 1). This suggested that P3N-ALT alone possesses the ability to support cell-to-cell movement of Cl-P3ΔARFPs, although this ability is weaker than that of P3N-PIPO. In the presence of both WCl/P3N-PIPO and WCl/P3N-ALT, Cl-P3ΔARFPs spread much further (Fig. 4b and Table 1). Cl-P3ΔARFPs moved even further when co-inoculated with WCl/P3N-PIPO(ALT^–1^, P3^+1^) (Fig. 4b and Table 1). This implies that appropriate expression levels of P3N-PIPO and P3N-ALT are required for the efficient cell-to-cell movement of ClYVV. Our results indicated that P3N-ALT functions to support viral cell-to-cell movement of ClYVV in collaboration with P3N-PIPO.

In another experiment in which broad bean plants were used, Cl-P3ΔARFPs co-inoculated with WCl/P3N-ALT and WCl/P3N-PIPO moved to systemic leaves, whereas Cl-P3ΔARFPs failed to infect the whole plant bodies when it was infected alone (Supplementary Fig. 4). This result suggests that regardless of plant species, alternative reading frame products of P3 are required for ClYVV infection and systemic spreading.

To rule out the possibility that a certain protein encoded by the vector part of WClMV contributed to the movement of Cl-P3ΔARFPs with P3N-PIPO or P3N-ALT, two experiments were conducted. First, Cl-P3ΔARFPs was co-inoculated with the WClMV empty vector, and it rarely moved to adjacent cells (Table 1). Second, we prepared two plasmids, pE2113/P3N-PIPO and pE2113/P3N-ALT, which exclusively express P3N-PIPO and P3N-ALT, respectively, under the control of the cauliflower mosaic virus 35S promoter. The Cl-P3ΔARFPs cDNA clone was biolistically co-inoculated with either of the plasmids into pea leaves, and the cell-to-cell movement was analysed. Cl-P3ΔARFPs was observed to spread to the neighbouring cells when either P3N-PIPO or P3N-ALT was supplied *in trans*, although the percentage of multicellular foci in which Cl-P3ΔARFPs moved to two or more cells was much lower with P3N-ALT than with P3N-PIPO (Table 1). This result indicated that P3N-PIPO and P3N-ALT themselves enable Cl-P3ΔARFPs to move cell-to-cell.

### P3N-PIPO and P3N-ALT are produced *via* TS of viral RdRp

We investigated whether the expression of P3N-PIPO and P3N-ALT resulted from FS or TS using *in vitro* analysis. Generally, FS is affected by codons at the A site and/or P site of the translating ribosome^10^,^27^,^42^. We introduced a GAAAAAAUUUG sequence into the *GFP* sequence in three different reading frames to generate *GFP-G*_*1*_*A*_*6*_(*f1*), *GFP-G*_*1*_*A*_*6*_(*f2*) and *GFP-G*_*1*_*A*_*6*_(*f3*) (Supplementary Fig. 5a). Note that the UUUG sequence following G_1_A_6_ is from RB. In these constructs, we designed the modified *GFP* genes to be produced in the –1 and +1 reading frames with molecular masses of 27 kDa and 24 kDa, respectively, in addition to a zero-frame 19 kDa protein product (Supplementary Fig. 5a). We analysed whether these *GFP* variants produce the alternative reading frame products *in vitro*. We found that *GFP-G*_*1*_*A*_*6*_(*f1*), *GFP-G*_*1*_*A*_*6*_(*f2*) and *GFP-G*_*1*_*A*_*6*_(*f3*) produced both –1 and +1 reading frame products with slightly different efficiencies (Supplementary Fig. 5b,c). Thus, regardless of the reading frame, the GAAAAAAUUUG sequence was able to induce production of the alternative reading frame proteins *in vitro*. Because FS efficiency is strongly dependent on the reading frames of slippery sequences^10^,^11^,^12^, the results suggest that FS was not a major cause of the expression of alternative reading frame products. Rather, TS occurred during mRNA preparation by SP6 RNA polymerase in our *in vitro* experiments. This raises the possibility that P3N-PIPO and P3N-ALT are produced by TS of the ClYVV-encoded RdRp *in planta.*

To examine the production of P3N-PIPO and P3N-ALT through TS by the viral RdRp (*i.e.*, NIb), the viral genomic region encompassing the G_2_A_6_ motif of ClYVV multiplied *in planta* was sequenced at a high coverage. To avoid possible carry-over of viral genomes containing insertions and/or deletions (indels), we used infectious cDNA clones as initial inocula instead of virions. The plasmids pCl30 and pRB carrying Cl30 or RB infectious cDNA, respectively, were biolistically inoculated into broad bean plants, which are suitable hosts for biolistic inoculations of leaves still attached to the plant. Viral RNA samples were prepared from systemically infected upper leaves. The 209 nucleotide (nt)-long DNA fragments were amplified using cDNA prepared from the total RNA. The first 49 nucleotides of the amplified fragments, containing the G_2_A_6_ motif, were sequenced using a HiSeq2000 instrument. As controls for indels potentially introduced during PCR amplification, library preparation and/or amplicon sequencing, the plasmid DNAs used for inoculations were also subjected to the same procedure.

**Figure 5:**
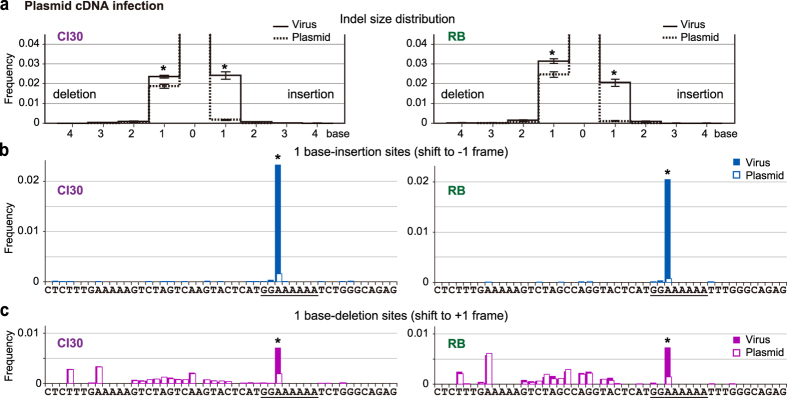
Site and frequency of transcriptional slippage at the ClYVV P3 region *in planta*. (**a**) Indel frequencies were estimated by a negative binomial regression using indel counts observed by amplicon sequencing of the *P3* region encompassing the G_2_A_6_ motif. Amplicons prepared from cDNA of systemically propagated Cl30 or RB virus (solid line), or from their parental plasmids used for inoculation (pCl30 or pRB; dashed line), were subjected to sequencing. Estimated frequencies of each size of indels are shown. Error bars indicate standard errors of the estimated frequencies. Asterisks indicate an indel size in which the viral RNA showed a higher frequency than the corresponding control plasmid (*p* <0.05, by the negative binomial regression). (**b**,**c**) The *P3* region encompassing the G_2_A_6_ motif was obtained from Cl30 and RB virus (filled bars), and their parental plasmids (open bars), and sequenced to analyse frequencies of transcriptional slippage to produce P3N-PIPO (**b**; one base insertion) and P3N-ALT (**c**; one base deletion). The sequences of the 49 analysed bases of Cl30 (left panels) and RB (right panels) *P3* are indicated under the graph. Fisher’s exact test was used to analyse the enrichment of indels in the viral cDNA at each site. The asterisks indicate a statistically significant enrichment of the indels in the viral cDNA (*q*-value <0.05).

After sequencing and quality filtering, 48,191, 31,313, 20,584 and 29,468 reads from Cl30, pCl30, RB and pRB, respectively, were obtained for downstream analyses. Amplicon sequencing revealed the sizes and frequencies of indels that occurred in the region during systemic infection and multiplication of viruses. The size of the indels in Cl30 and RB viral amplicons ranged from four base insertions to four base deletions, while indels in the plasmid amplicons ranged from two base insertions to three base deletions. Among the observed indels, one base insertions or deletions were significantly enriched in cDNA prepared from systemically infected viruses (Fig. 5a).

To examine if the occurrence of one base insertions or deletions was enriched at particular sites, we analysed the frequencies of indels and their statistical significance using Fisher’s exact test. One base insertions (–1 frame; Fig. 5b) or deletions (+1 frame; Fig. 5c) at the 33^rd^ (numbered from the amplicon’s 5′ end) adenine (33A) occurred significantly more in both Cl30 and RB compared with their parental plasmids used for inoculation. Since it is unable to identify the exact position where indels had occurred within a homopolymer stretch such as A_6_ by sequencing, we interpreted these results to mean that indels had been introduced within the A_6_ sequence rather than only at 33A during viral RNA replication *in planta*, Further analysis using a negative binomial model fitting with indel counts observed in three, five, seven or nine base-windows (the sizes that partially or entirely cover consecutive nucleotides in the G_2_A_6_ motif) also supported the result obtained from Fisher’s exact test (Supplementary Figs 6 and 7 and Supplementary Table 2). Another feature of the indel occurrence was that one base deletions were observed at various sites and more frequently than one base insertions. However, this pattern of deletion occurrence was highly correlated between the viral and plasmid samples and, in fact, no statistical difference was observed other than at 33A (Fig. 5c). It is likely that the deletion occurrence patterns contain technical artefacts. Thus, the results strongly suggest that viral RdRp-mediated TS occurs at the A_6_ sequence in the G_2_A_6_ of ClYVV, and that P3N-PIPO and P3N-ALT are produced in ClYVV-infected plants.

### Diverse viruses may produce frameshift products like P3N-ALT

**Figure 6:**
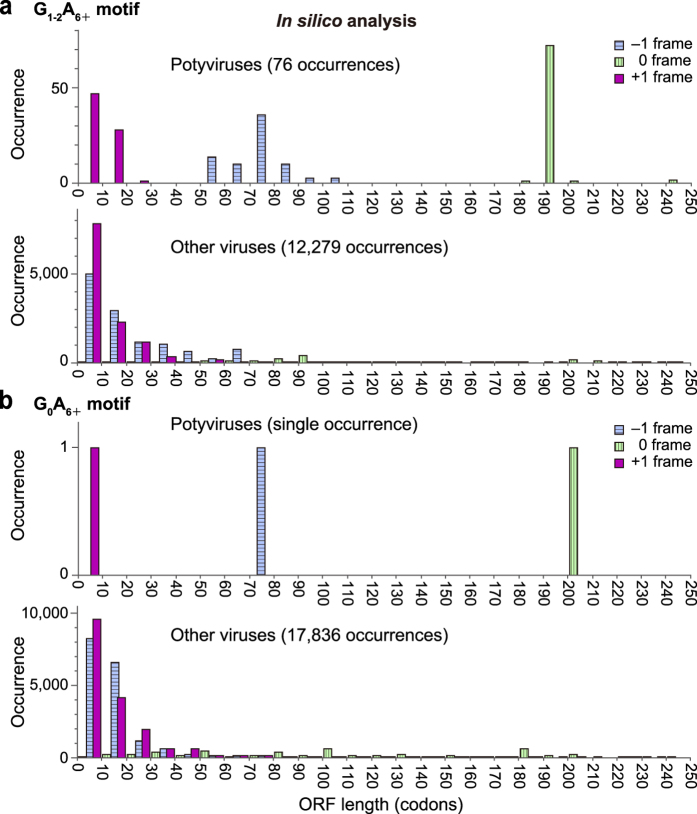
Distribution of lengths of ORFs potentially produced by transcriptional slippage (TS) in single-strand RNA viruses. A simulation was performed to obtain the amino acid sequences of proteins potentially produced by TS. Information on genomic sequences and ORF positions of single-strand RNA viruses were obtained from the NCBI website (http://www.ncbi.nlm.nih.gov/). ORF sequences with a 1 base insertion or deletion at the G_1–2_A_6+_(**a**) or G_0_A_6+_(**b**) motif were generated and translated into amino acid sequences and are presented for potyviruses (upper panels) and other viruses (lower panels). Magenta bar, blue bar with horizontal stripes and green bar with vertical stripes represent distribution of the –1, +1 and original reading frames, respectively. Differences in the numbers of entries among viral species in the database were normalized per site by selecting only one entry with the longest predicted amino acid sequence following the simulated indel of a virus when multiple entries for the virus with the same length, and the same start and stop codon coordinates of the original ORF containing the motif exist. Note that in (**b**), The G_0_A_6_ motif is found only in lupine mosaic virus (GenBank/EMBL/DDBJ Accession No. NC_014898), which does not carry the G_1–2_A_6+_motif^67^.

We carried out *in silico* analyses to identify P3N-ALT from other potyviruses (Supplementary Fig. 8). If indels occur at the conserved G_1–2_A_6_ motif, one to twenty ALT amino acid residues are expected to follow the motif in the +1 frame, while 56–101 amino acid residues are expected to follow the motif in the –1 frame (Fig. 6a). In ClYVV, ALT is five amino acids long, and in more than half (23/35) of potyvirus species analysed, ALT is no more than five amino acids long.

This structural feature and expression pattern of P3N-ALT suggested that truncated yet functional products produced *via* TS have not been identified in diverse RNA virus species, and that the coding capacity of the viruses may have been underestimated. TS has been detected in genome populations of potyviruses and other positive- and negative-strand RNA viruses and retroviruses^13^,^43^,^44^,^45^,^46^. We explored the sequences validated to be sufficient to cause TS in genomes of other RNA viruses. In addition to the G_1–2_A_6+_ motif, we searched for stretches of more than six adenines without a preceding guanine (hereafter referred to as G_0_A_6+_), because TS was reported to occur at G_0_A_6+_sequences in mammalian cells and *Escherichia coli*^47^,^48^. It is noteworthy that lupine mosaic virus (LuMV), which is a distantly related potyvirus^49^, is an exception in that it does not carry the G_1–2_A_6–7_ motif, but carries a G_0_A_6_ motif (Fig. 6b upper panel, Supplementary Fig. 8) at a similar position in the *P3* cistron as other potyviruses. It is likely that LuMV uses the G_0_A_6_ motif for the expression of P3N-PIPO and P3N-ALT. Full-length viral RNA sequences available from nucleotide sequence databases (GenBank, EMBL and DDBJ) revealed that out of 14,149 accessions, 5,133 carry a G_1–2_A_6+_ motif and 11,624 carry a G_0_A_6+_ motif (Supplementary Table 1). This indicates that 30–80% of known RNA viruses have motifs that potentially cause TS. Moreover, 77.5% and 77.0% of all expected products terminate within six codons downstream of the G_1–2_A_6+_ and G_0_A_6+_ motifs, respectively (Fig. 6a,b lower panels).

## Discussion

Our study uncovered another hidden component of ClYVV, P3N-ALT, and determined that the major mechanism that produces P3N-ALT is TS. The *P3* cistron of the ClYVV genome produces three proteins whose C-termini are different, P3 in the zero frame, P3N-PIPO in the –1 frame and P3N-ALT in the +1 frame. The results of western blot analyses, functional analyses, and high-coverage sequencing of viral genomic fragments *in planta* provided convincing evidence that P3N-ALT is expressed in ClYVV-infected plants, as is P3N-PIPO.

The expression of P3N-ALT seems to be a prerequisite for efficient infection by ClYVV (Table 1 and Fig. 4). Among the potyviruses, the *alt* ORF expressed in the +1 reading frame, unlike the *pipo* ORF that has 56–101 amino acid residues, has an extension of 1–20 amino acids from the G_1–2_A_6–7_ motif (Supplementary Fig. 8), and the extension of ClYVV is five amino acids. We detected P3N-ALT, which was transiently expressed as a frameshift product from the *P3* cistron of another potyvirus, BYMV, in *N. benthamiana* by agroinfiltration in this study (Fig. 2). Recent high-coverage sequencing of other potyviral genomes in plant tissues infected with TuMV, sweet potato feathery mottle virus, bean common mosaic virus and bean common mosaic necrosis virus detected indels that presumably cause +1 frameshifting for their P3N-ALT expression, as well as those for P3N-PIPO^31^,^32^. It is difficult to imagine that such short stretches of amino acids would endow some specific function to P3N-ALT. Therefore, we propose that P3N-ALT should be regarded as a C-terminal truncated form of P3.

Expression of truncated proteins such as P3N-ALT by a similar TS was also reported in negative-strand RNA viruses, including Ebola^44^ and Sendai viruses^45^, members of the family *Paramyxoviridae* and a positive-strand RNA virus, hepatitis C virus (HCV)^50^, a member of the family *Flaviviridae*. Because these truncated proteins are accompanied by another alternative reading frame product as in the case of ClYVV, in which P3N-ALT is accompanied by P3N-PIPO (Supplementary Fig. 1a), it is impossible to make a mutant virus that only lacks the truncated protein without affecting the other alternative reading frame product. Therefore, the functions of the truncated proteins remain to be determined. In this study, we used a heterologous virus vector, WClMV, and an expression cassette to provide either or both of the alternative reading frame products *in trans* to the infectious Cl-P3ΔARFPs that lacks both (Fig. 4, Supplementary Fig. 4, and Table 1). Our data suggest that P3N-ALT contributes to ClYVV virulence by functioning independently and with P3N-PIPO to facilitate cell-to-cell movement. P3N-PIPO localizes in plasmodesmata and assists the cell-to-cell movement of potyviruses by interacting with a host cation-binding protein, PCaP1, at its C-terminal PIPO domain^19^,^24^. How P3N-ALT, which lacks the PIPO domain, functions in cell-to-cell movement has yet to be determined.

Our analyses demonstrate that P3N-PIPO and P3N-ALT can be produced from RNA genomes through indels that occurred by TS. It is intriguing that viral RNAs harbouring indels are used to produce proteins required for viral infection by expanding the protein coding capacity. Although TS occurs at an adenine stretch in HCV^13^, TS may be used only by a few viral species, such as potyviruses and HCV. However, our *in silico* analysis showed that 30–80% of known RNA viruses have motifs that potentially cause TS (Fig. 6 and Supplementary Table 1). This may suggest that alterations of protein coding capacity *via* TS may be prevalent in RNA viruses.

In positive-strand RNA viruses like ClYVV, TS may not be ideal for expanding protein coding capacity because unlike FS, TS in positive-strand RNA viruses inevitably causes the accumulation of indels in their viral genomes. Therefore, for TS to be a viable option for positive-strand RNA viruses to expand their coding capacity, there must be a way to remove genomic RNAs with indels. A possible mechanism to eliminate RNA genomes with indels in potyviruses is nonsense-mediated mRNA decay (NMD)^51^,^52^,^53^. Recently, Garcia *et al.*^54^ demonstrated that RNAs of positive-strand RNA viruses that synthesize subgenomic RNAs trigger the NMD pathway. This is because, except for the 3′-most subgenomic RNA, the natural termination codon is recognized as a premature termination codon due to the presence of a long 3′-untranslated region. In their report, the potyviral genome was shown to evade degradation by NMD because potyviruses use the polyprotein strategy and the termination codon is located near the 3′ end of the genomic RNA. This, in turn, suggests that the ClYVV genomic RNAs that harbour indels producing P3N-PIPO or P3N-ALT will be selectively eliminated by NMD because the termination codons of P3N-PIPO and P3N-ALT are located more than 6,000 nucleotides from the 3′ end, which will induce strong NMD^55^. Mahajan *et al.*^56^ demonstrated that in tobacco etch virus (TEV), which is a potyvirus, the RNA genome has to be translated through at least one half of the *CP* cistron to be efficiently replicated. They showed that mutant TEV genomes containing a stop codon within the 5′ half of the *CP* cistron did not accumulate in tobacco cells, despite the dispensability of CP during replication. The NMD mechanism might mediate the elimination of mutated TEV genomic RNA, although there remains a possibility that only genomic RNAs that have been translated throughout the polyprotein ORF are positively selected as replication templates.

One of the notable findings of our study is that P3N-ALT (*i.e*., the truncated P3) is a functional protein during ClYVV infection (Supplementary Fig. 1a). Indels introducing a premature stop codon to produce a truncated protein are generally considered to be detrimental and useless mutations and have not been the subject of further functional analyses. As mentioned above, the G_1_A_6+_ and G_0_A_6+_ motifs, which are sufficient to cause TS^47^,^48^, are prevalent in the genome sequences of a diverse range of RNA viruses. This study raises the possibility that functional truncated proteins produced by an expansion of the protein coding capacity *via* TS may exist in other RNA viruses (Fig. 6, Supplementary Table 1 and Supplementary Fig. 1b).

In summary, we identified P3N-ALT, a hidden component of ClYVV, which is most likely produced *via* TS of the viral RdRp. Our findings not only add another repertoire to viral functions, but also imply that numerous RNA viruses may use TS to produce as yet unidentified protein products.

## Methods

### Viruses

The infectious clone of ClYVV isolate no. 30 containing a coding sequence for GFP, pClYVV/C3-S65T-Sal^57^ and its derived virus are referred to pCl30 and Cl30, respectively, in this study. RB is a chimeric ClYVV between two isolates, no. 30 and highly virulent 90-1 Br2 (ref. 38), and its infectious clone (referred to as pRB) was used as a virulent ClYVV in this study. BYMV-CS that was previously isolated from red clover^39^ and the WClMV vector were also used, as described below^40^.

### Plasmids

For the *in vitro* translation assay, the P3 regions of Cl30 (ref. 36) and RB^38^ derived from in pE2113/Cl30-P3 and pE2113/RB-P3, respectively^38^, were cloned into the pSP64 Poly(A) vector (Promega, Madison, WI, USA) using the *Bam*HI and *Sac*I sites. The resulting plasmids, pSP/Cl30-P3 and pSP/RB-P3, were used as templates for *in vitro* transcription reactions and to generate their variants. The variants were produced by site-directed mutagenesis using an inverse PCR technique^58^. pSP/Cl30-P3 was used as a template to construct pSP/Cl30-P3N-PIPO^mk^ (to express P3N-PIPO size marker for Cl30) and pSP/Cl30-P3N-ALT^mk^ (to express P3N-ALT size marker for Cl30). pSP/RB-P3 was used as a template to construct pSP/RB-P3N-PIPO^mk^ (to express P3N-PIPO size marker for RB) and pSP/RB-P3N-ALT^mk^ (to express P3N-ALT size marker for RB). The primers used for construction are shown in Supplementary Table 3. Inverse PCR was conducted using KOD-Plus-Neo (TOYOBO, Osaka, Japan). Following the digestion of the template plasmid DNA with *Dpn*I, PCR products were phosphorylated by T4 polynucleotide kinase and circularized using a Ligation-Convenience kit (Nippon Gene, Tokyo, Japan).

To construct plasmids for the agroinfiltration assay, we first generated vector pTA/XhSp-3FLAG, which is a modified version of pTA7001 (ref. 59). We generated a DNA fragment (5′-CTCGAGatggccACTAGTggtggaagtggaggtagtggtggaagtggaggtagtATGGACTACAAAGACCATGACGGTGATTATAAAGATCATGACATCGATTACAAGGATCATGATGGGtaaTCTAGA-3′) harbouring *Xho*I site, *Spe*I site, Gly/Ser linker, three copies of flag epitope tag coding sequence^60^, and *Xba*I site, by an overlap extension PCR. The PCR product was digested with *Xho*I and *Xba*I and inserted between the *Xho*I and *Spe*I sites of pTA7001 to generate pTA/XhSp-3FLAG.

The region between the 5′ end of the *P3* cistron and 3′ end of *pipo* was amplified by PCR with primers 1009 and 1011 (Supplementary Table 3) using WClMV vectors, P3^+P3N-PIPO^ and P3N-PIPO^41^, as template DNAs to generate pTA/RB-P3(PIPO:FLAG^–1^) and pTA/RB-P3N-PIPO:FLAG^mk^, respectively. The cDNA product for pTA/Cl30-P3(PIPO:FLAG^–1^) was amplified by PCR using pCl30 (ref. 61) as a template and primers 1009 and 1012 (Supplementary Table 3). The cDNA product for pTA/Cl30-P3N-PIPO:FLAG^mk^ was amplified by two-step PCR using pCl30 as a template and primers 1009, 1012, P3as3 and P3s3, as described previously^41^. The cDNA product for pTA/CS-P3(PIPO:FLAG^–1^) was amplified by PCR using the infectious clone of BYMV-CS^39^ as a template and primers 1010 and 1013 (Supplementary Table 3). The cDNA product for pTA/CS-P3N-PIPO:FLAG^mk^ was amplified by two-step PCR using the BYMV infectious clone as a template and primers 1010, 1013, 3669 and 3668 (Supplementary Table 3), as described previously^41^. The PCR products were digested with *Xho*I and *Spe*I, and inserted into pTA/XhSp-3FLAG.

The region between the 5′ end of the *P3* cistron and 3′ end of *alt* was PCR amplified using primers 1009 and 1124 (Supplementary Table 3) with plasmid pCl30 as the template, primers 1009 and 1123 (Supplementary Table 3) with plasmid of the WClMV vector that expresses P3 of RB in infected plants as the template^41^, and primers 1010 and 1125 with plasmid the BYMV infectious clone as the template to generate pTA/Cl30-P3(ALT:FLAG^+1^), pTA/RB-P3(ALT:FLAG^+1^) and pTA/CS-P3(ALT:FLAG^+1^), respectively. To generate pTA/Cl30-P3N-ALT:FLAG^mk^, pTA/RB-P3N-ALT:FLAG^mk^ and pTA/CS-P3N-ALT:FLAG^mk^, the cDNA fragments were amplified by PCR using primers 1009 and 1350 (Supplementary Table 3) with the plasmid pTA/Cl30-P3(ALT:FLAG^+1^) as the template, primers 1009 and 1349 (Supplementary Table 3) with plasmid pTA/RB-P3(ALT:FLAG^+1^) as the template and primers 1010 and 1351 (Supplementary Table 3) with plasmid pTA/CS-P3(ALT:FLAG^+1^) as the template, respectively. The amplified fragments were introduced into pTA/XhSp-3FLAG using *Xho*I and *Spe*I sites. The binary vector carrying a gene for yellow fluorescent protein (YFP) was prepared as follows. The cDNA of the *YFP* gene with the *Xho*I and *Spe*I sites was amplified by PCR using pGWB641 (ref. 62) with primers 796 and 797 (Supplementary Table 3). The PCR fragment was inserted in pTA7001 between the *Xho*I and *Spe*I sites to generate pTA/YFP, and was used for *Agrobacterium*-mediated transient expression experiments.

For the cell-to-cell movement assay, we modified the *P3* cistron of the infectious ClYVV clone, Cl30 (ref. 38), as follows. A Cl30 mutant that was defective in the expression of both P3N-PIPO and P3N-ALT (pCl-P3ΔARFPs) was made by substituting guanine and thymine for the second adenine of and the adenine eight nucleotides downstream from the G_2_A_6_ motif, respectively, as described previously^38^ using primers delPIPO_s and delPIPO_as (Supplementary Table 3). We prepared the transient expression vector pE2113 expressing P3N-ALT under con-trol of 35S promoter (pE2113/P3N-ALT) and the WClMV vector expressing P3N-ALT (pWCl/P3N-ALT), by inserting the Cl30 *P3* cistron that had exchanged the G_2_A_6_ motif for the GGAGAAA sequence into the *Bam*HI-*Sac*I site of pE2113 and the *Spe*I and *Xho*I site of the WClMV vector, as described previously^38^ using primers afs_s and afs_as (Supplementary Table 3). Similarly, pE2113/P3N-PIPO and pWCl/P3N-PIPO, which express P3N-PIPO of Cl30 exclusively, were constructed using primers P3as3 and P3s3 (Supplementary Table 3). The WClMV vector that mainly expressed P3N-PIPO, with low amounts of P3N-ALT, and P3 was constructed previously (pWCl/Cl30_P3N-PIPO)^38^. This WClMV vector was redesignated as WCl/P3N-PIPO(ALT^–1^, P3^+1^) in this study.

### *In vitro* transcription and translation

The plasmid DNAs were linearized with *Eco*RI and extracted with phenol/chloroform (1:1 v/v), followed by ethanol precipitation. RNA was synthesized from the linearized plasmid DNA using the AmpliCap SP6 high-yield message maker kit (Cellscript, Madison, WI, USA) in the presence of a cap analogue, according to the manufacturer’s instructions. After the transcription reaction, the template DNA was digested with RNase-free DNase I and the mRNA was purified using an RNeasy Mini kit (Qiagen, Hilden, Germany).

Translation reaction cocktail (15 μl) for WGE (Promega) was prepared according to a previous report^63^, with slight modifications. The reaction mixture contained 7.5 μl of WGE, 1.2 μl of 1 mM amino acid mixture lacking methionine, 0.17 μl of 5 μM L-methionine, 12 units of RNasin (Promega), approximately 300 ng of mRNA and 0.13 μl of [^35^S]methionine (43.5 TBq mmol^–1^, American Radiolabeled Chemicals, St. Louis, MO, USA). The mixture was incubated at 25 °C for 90 min.

After incubation, the translation products were separated on a NuPAGE 4–12% Bis-Tris Gel (ThermoFisher Scientific, Waltham, MA, USA) with MES running buffer (Invitrogen). The protein bands were visualized using a FLA-7000 image analyzer (Fuji Photo Film, Tokyo, Japan). For quantification, the acquired images were processed using the MultiGauge (Fuji Photo Film). The band intensities were measured using ImageJ [NIH (http://rsbweb.nih.gov/ij)]. The % accumulation level for the –1 frame product A_–1_ was determined as follows:


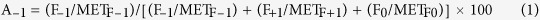


where the band intensity for each zero-frame, –1 frame, and +1 frame products are denoted by F_0_, F_–1_ and F_+1_, respectively, and the number of methionine codons in each product is denoted by MET_F0_, MET_F–1_ and MET_F+1_, respectively. A_1_ was determined accordingly.

### Agrobacterium-mediated transient expression

Agrobacterium-mediated transient expression was conducted as described previously^41^. Agrobacterium LBA4404 cells transformed with each construct were suspended in MES buffer [10 mM 2-(*N*-morpholino)ethanesulfonic acid (MES), 10 mM MgCl_2_, pH 5.7], and the suspensions were adjusted to OD_600_ = 1.0. Acetosyringone was added to the suspensions (final concentration, 200 μM), followed by incubation at room temperature for 2–4 h. The suspensions were infiltrated into *N. benthamiana* leaves using needleless syringes. Leaves were sprayed with 30 μM DEX solution containing 0.01% Tween-20 one day after agroinfiltration^59^. Leaves were collected at 1 day after DEX treatment and used for western blot analysis.

Western blotting was conducted as described previously^64^. Proteins were electrophoresed through a 12% NuPAGE Bis-Tris gel (ThermoFisher Scientific) in MES-SDS buffer, followed by electrotransfer to a PVDF membrane. To detect the FLAG-tagged proteins, monoclonal ANTI-FLAG M2-Peroxidase Clone M2 (Sigma-Aldrich, MO, USA) was used at a 1:5000 dilution. The chemiluminescence signals were detected with ECL Prime or Select (GE Healthcare, Little Chalfont, Buckinghamshire, UK) using an LAS-4000 (GE Healthcare).

### Detection of the P3N-ALT protein in ClYVV-infected plants

To detect P3N-ALT, rabbit polyclonal antibodies were raised against the partial peptide of the N-terminal region of ClYVV P3, SLTGQVIQFDTKMLIS. After fractionation of the antibodies that possessed affinity to the immunized peptide, their specificity to the N-terminal region of P3 was confirmed (Supplementary Fig. 9). Western blotting to detect P3N-ALT in samples from ClYVV-infected plants was carried out with a 1000-fold diluted sample of the affinity fractionated antibody solution, as described previously^38^.

### Functional analysis of P3N-ALT by ClYVV infection

Detached leaves of a susceptible pea line, PI 250438, were biolistically co-inoculated with tungsten particles coated with 800 ng of pCl-P3ΔARFPs and 200 ng of either of the pE2113 and WClMV vectors, as described previously^38^,^65^. The inoculated leaves were kept in petri dishes with moistened filter paper. The GFP signal was monitored using an epifluorescence microscope (VB 7010; Keyence, Osaka, Japan).

### Image data acquisition and data processing

For GFP fluorescence and gel images, the acquired images were processed using Photoshop 12.0.4 or CS5 software (Adobe Systems, San Jose, CA, USA). For gel images of Western blotting and autoradiograms, contrast was adjusted for the sake of visibility as appropriate.

### Amplicon sequencing analysis of ClYVV genomic RNAs *in planta*

ClYVV infectious plasmid clones, Cl30 and RB^38^, were biolistically inoculated into the first or second pair of true leaves from bottom of three broad bean plants, which were grown independently in separated plastic pots for two weeks, as described above. Systemically propagated viral RNA in upper leaves that showed symptoms at 7 days post-inoculation was analysed. Total RNA was extracted from these upper leaves using the TRIzol reagent, according to the manufacture’s manual (ThermoFisher Scientific). The cDNA was synthesized using ReverTra Ace (TOYOBO) with random 9-mers in a mixture containing 500 ng of total RNA. The 209-nt amplicons were prepared in 50 μl reaction mixtures containing 1 μl of cDNA solutions, 25 μl 2 × PCR buffer for KOD-FX neo (TOYOBO), 0.4 mM dNTPs, 1 U KOD-FX neo (TOYOBO) and 0.3 μM primer pairs: 3879 and 3881 for samples of Cl30; and 3880 and 3882 for samples of RB (Supplementary Table 3). The PCR conditions were: 94 °C for 2 min, followed by 20 cycles of denaturation at 98 °C for 30 s, annealing at 62 °C for 30 s and elongation at 68 °C for 2 min. The mixture was incubated at 72 °C for 7 min following completion of the last cycle. The amplicons were also prepared from 10 pg of parental infectious plasmid DNAs. One ng of the amplicons was used to prepare libraries for sequencing using a HiSeq2000 (Illumina, San Diego, CA, USA). The library preparation was performed using a modified protocol for double digest restriction-associated DNA sequencing^66^. Briefly, the amplicons were digested with 5 units of *Bgl*II and 2.5 units of *Nde*I (New England Biolabs, Ipswich, MA, USA) simultaneously, with adapter ligation using T4 DNA Ligase (Enzymatics, Beverly, MA, USA). Amplicon-adaptor complexes were purified using Agencourt AMPure XP (Beckman Coulter, Brea, CA, USA) and were amplified by PCR with index and universal primers.

The raw sequence reads were aligned using BWA 0.7.10 software (http://sourceforge.net/projects/bio-bwa/) to the 209 nt sequence of Cl30 or RB. We did not filter the raw reads before alignment but did remove reads with bases with low quality values around indels after the alignment. The quality values of inserted bases, and bases adjacent to inserted or deleted base(s) were analysed. The threshold of the quality value was set to 30. Base substitutions in the reads were not analysed in this study. Counts of reads with indels and total reads for each sample were obtained from the reads remaining after filtering. To analyse enrichment of particular sizes of indels, the counts were then fitted to a negative binomial model:


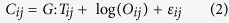


where *C*, *G*, *T*, *O*, and *ε* are number of counts with a particular type of indels, genotype of virus (*i.e.*, Cl30 or RB), type of RNA sample (*i.e.*, inoculated plasmid or propagated virus), total number of reads of the sample, and residual, respectively. *O* is the log link function. Frequencies of indels per site were analysed by Fisher’s exact test using R software (3.1.2) and the glm.nb function in the MASS package (7.3–35).

### Viral sequence analysis and simulation of translation products produced by TS

Full-length genomic sequences of RNA viruses were downloaded in the GenBank format from the NCBI website (http://www.ncbi.nlm.nih.gov/). The sequences were semi-automatically curated to exclude non-RNA viral genomes using a custom Perl script and by manual inspection. ORFs with annotations for noncanonical codon usage or in an unconventional translational manner, such as ribosomal frameshift were excluded. Next, using a custom Perl script, (i) sequences of ORFs with G_1–2_A_6+_ and G_0_A_6+_ motifs were parsed, (ii) translation products produced from RNA genomes with +1 or –1 base indels at each G_1–2_A_6+_ or G_1_A_6+_ motif were simulated, and (iii) the lengths of the simulated peptides were recorded. The length of a translation product of an ORF that contained no termination codons in the reading frame changed by a simulated indel was set to 0. In order to normalize difference of the number of entries of viral species in the database, the list of the motif site and the length of simulated peptides was then normalized per motif site by selecting only one entry (accession) with the longest predicted amino acid sequence following the simulated indel of a virus when multiple entries for the virus with the same length, and the same start and stop codon coordinates of the original ORF containing the motif exist.

## Additional Information

**How to cite this article**: Hagiwara-Komoda, Y. *et al.* Truncated yet functional viral protein produced *via* RNA polymerase slippage implies underestimated coding capacity of RNA viruses. *Sci. Rep.* **6**, 21411; doi: 10.1038/srep21411 (2016).

## Supplementary Material

Supplementary Information

## Figures and Tables

**Table 1:** Effect of supplementing P3N-PIPO and P3N-ALT *in trans*.

ClYVV type	Co-inoculated plasmid(s)	Number of observed foci^a^		Cell-to-cell moving
		Single cell	Multicellular^b^	
Cl30	–	9	22	71%
Cl-P3ΔARFPs	–	35	0	0%
Cl-P3ΔARFPs	WCl/P3N-PIPO	10	25	71%
Cl-P3ΔARFPs	WCl/P3N-ALT	29	5	15%
Cl-P3ΔARFPs	WCl/P3N-ALT +WCl/P3N-PIPO	25	101	80%
Cl-P3ΔARFPs	WCl/P3N-PIPO(ALT^–1^, P3^+1^)	3	22	88%
Cl-P3ΔARFPs	WCl/empty^c^	27	1	4%
Cl-P3ΔARFPs	pE2113/P3N-PIPO	13	25	66%
Cl-P3ΔARFPs	pE2113/P3N-ALT	27	9	25%

^a^Foci were observed at 3 days post-inoculation.

^b^Foci with diameters of two or more cells were considered multicellular.

^c^empty vector.
