# Supplementary material for: Truncated yet functional viral protein produced via RNA polymerase slippage implies underestimated coding capacity of RNA viruses
Source: Sci Rep. 2016 Feb 22;6:21411. doi: 10.1038/srep21411 (PMC4761962; doi:10.1038/srep21411)
Supplement: Supplementary Information [file srep21411-s1.pdf]

Supplementary Information

Truncated yet functional viral protein produced *via* RNA polymerase slippage implies underestimated coding capacity of RNA viruses

Yuka Hagiwara-Komoda*,§, Sun Hee Choi*, Masanao Sato*,§, Go Atsumi, Junya Abe, Junya Fukuda, Mie N. Honjo, Atsushi J. Nagano, Keisuke Komoda, Kenji S. Nakahara§, Ichiro Uyeda & Satoshi Naito§

* These authors contributed equally to this work.

§ These authors jointly supervised this work.

This file includes:

Supplementary Figures 1–9

Supplementary Tables 1–3

Supplementary Methods

Supplementary References

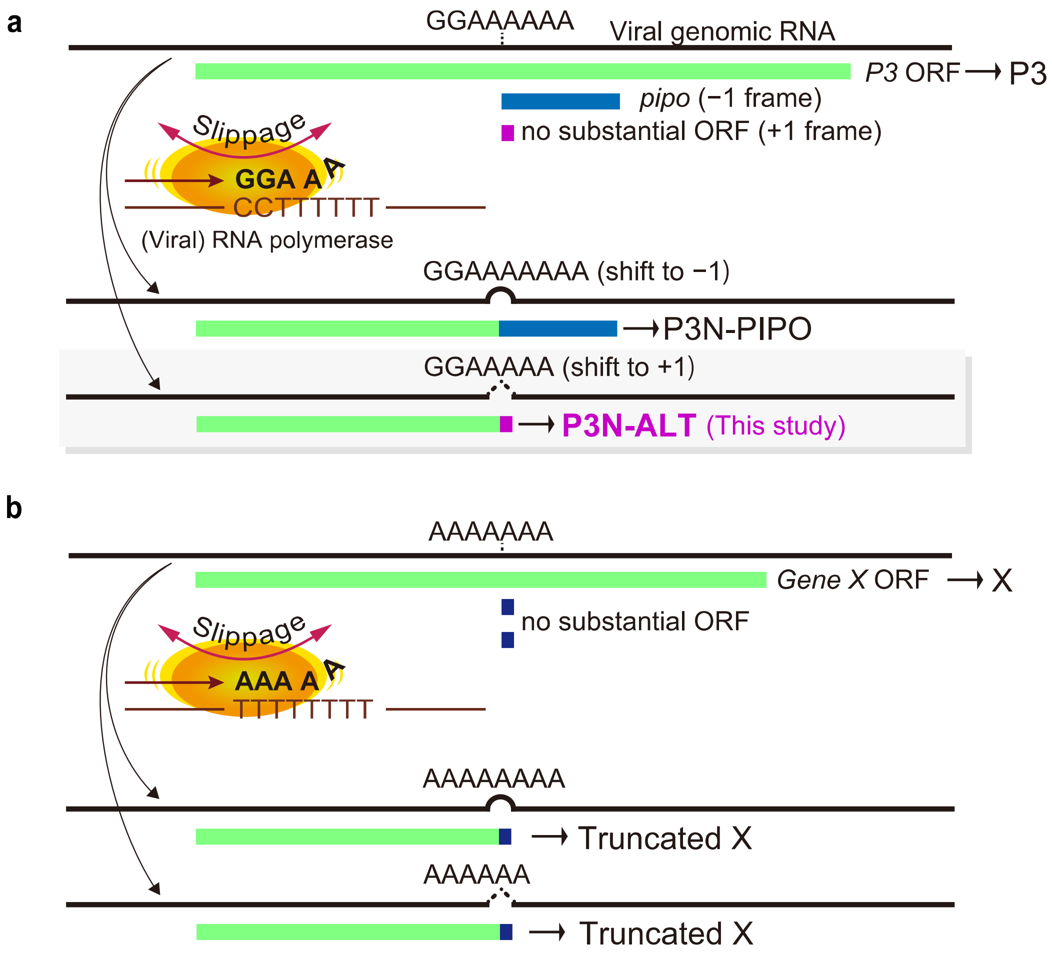

Supplementary Figure 1. Schematic model of the production of viral protein truncations *via* transcriptional slippage (TS) by viral RNA polymerases. (**a**) A small ORF exists in the *P3* cistron at the –1 reading frame in the large ORF that encodes the potyvirus polyprotein. Potyviruses produce the P3N-PIPO protein *via* TS by viral RNA polymerase NIb at the $G_{1\text{-}2}A_{6\text{-}7}$ motif. This study revealed that TS also causes frameshift to the +1 reading frame to produce P3N-ALT. Although P3N-ALT is considered a truncation of P3 because there is no substantial ORF at the +1 reading frame following to the motif, it contributes to the cell-to-cell movement of ClYVV in infected plants. (**b**) The findings in this study imply that truncated, yet functional, proteins from a certain gene *X* can be produced *via* TS, even without a substantial ORF in alternative reading frames of the RNA genomes of other viruses. In fact, as unveiled in this study, a number of $G_{1\text{-}2}A_{6\text{-}7}$ and $G_0A_{6\text{-}7}$ motifs with no substantial ORFs in one or two alternative frames are found in a diverse range of RNA viruses. Our study implied that not only a fusion protein such as P3N-PIPO shown in panel (**a**), but also a truncated protein as P3N-ALT could be produced *via* TS that causes a frameshift to an alternative frame where no substantial ORF exists.

RB-P3(PIPO:FLAG^{-1})

P3N’ (34.3 kDa)
MGKSLTGQVIQFDTKMLISSIYRPRQMEMIINEEPFVLVLAMQSPSVLLALFNSASLEKAVEVWLHKDMRVSHVMTMLALLAAKVSAAKMVNLQMEIIEA
SASHFLAAMDTIHKPMHSINTANIFLMNLEEGRSTDRTIDELGFHSLKKSSQVLMEKIWAEDLEQQWLGLRLSQKFYLIRQSWKQRAKYSKILAQRDELG
ASDKFSASLRLSATSIKNQAISCRKRMVATRLVVEVEVVVEVEVVWTTKTMTVIIKIMTSITRIMMGKLDDPGLPAFVRIIGFDNVRQVQCISFIAHTPE
SY*

P3N-PIPO (29.8 kDa)
MGKSLTGQVIQFDTKMLISSIYRPRQMEMIINEEPFVLVLAMQSPSVLLALFNSASLEKAVEVWLHKDMRVSHVMTMLALLAAKVSAAKMVNLQMEIIEA
SASHFLAAMDTIHKPMHSINTANIFLMNLEEGRSTDRTIDELGFHSLKKSSQVLMEKNLGRGFRAAMARFKIVTKVLFNKAVMEAAGKVFQNISPERRAR
CQRQVQRITQIVSNKHQKPSNQLQEENGRNKTSGGSGGSGGSGGSM**DYKDHDGDYKDHDIDYKDHDG***

P3N-ALTy (18.2 kDa)
MGKSLTGQVIQFDTKMLISSIYRPRQMEMIINEEPFVLVLAMQSPSVLLALFNSASLEKAVEVWLHKDMRVSHVMTMLALLAAKVSAAKMVNLQMEIIEA
SASHFLAAMDTIHKPMHSINTANIFLMNLEEGRSTDRTIDELGFHSLKKSSQVLMEKFGQRI*

Cl30-P3(PIPO:FLAG^{-1})

P3N’ (32.5 kDa)
MGKSLTGQVIQFDTKMLISSIYRPRQMEKIINEEPFVLVLAMQSPSVLLALFNSASLEKAVEVWLHKDMRVSHVMTMLALLAAKVSAAKMVNLQMEIIEA
SAGHFLAAMDTIHKPMHSINTANIFLMNLEEGRSTDRTIDELGFHSLKKSSQVLMEKIWAEDLEQQWLGLRLSQKFYLIKQSWKQRAKYSKILAQRDELG
ASDKFSASLRLSVTSILVVEVEVVVEVEVVWTTKTMTVIIKIMTSITRIMMGKLDDPGLPAFVRIIGFDNVRQVQCISFIAHTPESY*

P3N-PIPO (28.0 kDa)
MGKSLTGQVIQFDTKMLISSIYRPRQMEKIINEEPFVLVLAMQSPSVLLALFNSASLEKAVEVWLHKDMRVSHVMTMLALLAAKVSAAKMVNLQMEIIEA
SAGHFLAAMDTIHKPMHSINTANIFLMNLEEGRSTDRTIDELGFHSLKKSSQVLMEKNLGRGFRAAMARFKIVTKVLFNKAVMEAAGKVFQNTSPERRAR
CQRQVQRITQIVSNKHTSGGSGGSGGSGGSM**DYKDHDGDYKDHDIDYKDHDG***

P3N-ALT (18.1 kDa)
MGKSLTGQVIQFDTKMLISSIYRPRQMEKIINEEPFVLVLAMQSPSVLLALFNSASLEKAVEVWLHKDMRVSHVMTMLALLAAKVSAAKMVNLQMEIIEA
SAGHFLAAMDTIHKPMHSINTANIFLMNLEEGRSTDRTIDELGFHSLKKSSQVLMEKSGQRI*

CS-P3(PIPO:FLAG^{-1})

P3N’ (35.3 kDa)
MGNQSVGSHIRIDTELLVKSVYKPELMAHIIEQEPFLLVLAMQSPATLMALFNSSSLEKAVQYWLHKDMQVSHIMTMLAVLASNVSASKLLTTQFEVIEA
SAPQILAAMDHVYKPMHSINTANTFLMNLNESRETDKTIDELGFYSFKKSTRILMEKTLMADLDQQWQELGLLERFSLIKRSWQVRAKYSSFAIQREEKG
ITDKFTTSLRLSGAQIKHQALAKRDQIVRFAERRVERTLVVEVEVVVEVEVVWTTKTMTVIIKIMTSITRIMMGKLDDPGLPAFVRIIGFDNVRQVQCIS
FIAHTPESY*

P3N-PIPO (30.6 kDa)
MGNQSVGSHIRIDTELLVKSVYKPELMAHIIEQEPFLLVLAMQSPATLMALFNSSSLEKAVQYWLHKDMQVSHIMTMLAVLASNVSASKLLTTQFEVIEA
SAPQILAAMDHVYKPMHSINTANTFLMNLNESRETDKTIDELGFYSFKKSTRILMEKNLDGGLRSTMARIRIVGKILFNKAIVASASKVFKFCNSERRER
YHRQVHNLTQVIRGTNKAPGPCQERSNCAICREKSREDTSGGSGGSGGSGGSM**DYKDHDGDYKDHDIDYKDHDG***

P3N-ALT (17.8 kDa)
MGNQSVGSHIRIDTELLVKSVYKPELMAHIIEQEPFLLVLAMQSPATLMALFNSSSLEKAVQYWLHKDMQVSHIMTMLAVLASNVSASKLLTTQFEVIEA
SAPQILAAMDHVYKPMHSINTANTFLMNLNESRETDKTIDELGFYSFKKSTRILMEKP*

RB-P3(ALT:FLAG^{+1})

P3N’ (18.4 kDa)
MGKSLTGQVIQFDTKMLISSIYRPRQMEMIINEEPFVLVLAMQSPSVLLALFNSASLEKAVEVWLHKDMRVSHVMTMLALLAAKVSAAKMVNLQMEIIEA
SASHFLAAMDTIHKPMHSINTANIFLMNLEEGRSTDRTIDELGFHSLKKSSQVLMEKIWAEDY*

P3N-PIPO’ (26.2 kDa)
MGKSLTGQVIQFDTKMLISSIYRPRQMEMIINEEPFVLVLAMQSPSVLLALFNSASLEKAVEVWLHKDMRVSHVMTMLALLAAKVSAAKMVNLQMEIIEA
SASHFLAAMDTIHKPMHSINTANIFLMNLEEGRSTDRTIDELGFHSLKKSSQVLMEKNLGRGLLVVEVEVVVEVEVVWTTKTMTVIIKIMTSITRIMMGK
LDDPGLPAFVRIIGFDNVRQVQCISFIAHTPESY*

P3N-ALT:FLAG (21.9 kDa)
MGKSLTGQVIQFDTKMLISSIYRPRQMEMIINEEPFVLVLAMQSPSVLLALFNSASLEKAVEVWLHKDMRVSHVMTMLALLAAKVSAAKMVNLQMEIIEA
SASHFLAAMDTIHKPMHSINTANIFLMNLEEGRSTDRTIDELGFHSLKKSSQVLMEKFGQRITSGGSGGSGGSGGSM**DYKDHDGDYKDHDIDYKDHDG***

Cl30-P3(ALT:FLAG^{+1})

P3N’ (18.4 kDa)
MGKSLTGQVIQFDTKMLISSIYRPRQMEKIINEEPFVLVLAMQSPSVLLALFNSASLEKAVEVWLHKDMRVSHVMTMLALLAAKVSAAKMVNLQMEIIEA
SAGHFLAAMDTIHKPMHSINTANIFLMNLEEGRSTDRTIDELGFHSLKKSSQVLMEKIWAEDY*

P3N-PIPO’ (26.2 kDa)
MGKSLTGQVIQFDTKMLISSIYRPRQMEKIINEEPFVLVLAMQSPSVLLALFNSASLEKAVEVWLHKDMRVSHVMTMLALLAAKVSAAKMVNLQMEIIEA
SAGHFLAAMDTIHKPMHSINTANIFLMNLEEGRSTDRTIDELGFHSLKKSSQVLMEKNLGRGLLVVEVEVVVEVEVVWTTKTMTVIIKIMTSITRIMMGK
LDDPGLPAFVRIIGFDNVRQVQCISFIAHTPESY*

P3N-ALT:FLAG (21.8 kDa)
MGKSLTGQVIQFDTKMLISSIYRPRQMEKIINEEPFVLVLAMQSPSVLLALFNSASLEKAVEVWLHKDMRVSHVMTMLALLAAKVSAAKMVNLQMEIIEA
SAGHFLAAMDTIHKPMHSINTANIFLMNLEEGRSTDRTIDELGFHSLKKSSQVLMEKSGQRITSGGSGGSGGSGGSM**DYKDHDGDYKDHDIDYKDHDG***

CS-P3(ALT:FLAG^{+1})

P3N’ (18.0 kDa)
MGNQSVGSHIRIDTELLVKSVYKPELMAHIIEQEPFLLVLAMQSPATLMALFNSSSLEKAVQYWLHKDMQVSHIMTMLAVLASNVSASKLLTTQFEVIEA
SAPQILAAMDHVYKPMHSINTANTFLMNLNESRETDKTIDELGFYSFKKSTRILMEKTY*

P3N-PIPO’ (25.9 kDa)
MGNQSVGSHIRIDTELLVKSVYKPELMAHIIEQEPFLLVLAMQSPATLMALFNSSSLEKAVQYWLHKDMQVSHIMTMLAVLASNVSASKLLTTQFEVIEA
SAPQILAAMDHVYKPMHSINTANTFLMNLNESRETDKTIDELGFYSFKKSTRILMEKNLLVVEVEVVVEVEVVWTTKTMTVIIKIMTSITRIMMGKLDDP
GLPAFVRIIGFDNVRQVQCISFIAHTPESY*

P3N-ALT:FLAG (21.5 kDa)
MGNQSVGSHIRIDTELLVKSVYKPELMAHIIEQEPFLLVLAMQSPATLMALFNSSSLEKAVQYWLHKDMQVSHIMTMLAVLASNVSASKLLTTQFEVIEA
SAPQILAAMDHVYKPMHSINTANTFLMNLNESRETDKTIDELGFYSFKKSTRILMEKPTSGGSGGSGGSGGSM**DYKDHDGDYKDHDIDYKDHDG***

Supplementary Figure 2. Amino acid sequence of the constructs used in Fig. 2. Amino acid sequences of the three alternative reading frame products are shown for each of *RB-P3(PIPO:FLAG^{-1})*, *Cl30-P3(PIPO:FLAG^{-1})*, *CS-P3(PIPO:FLAG^{-1})*, *RB-P3(ALT:FLAG^{+1})*, *RB-P3(ALT:FLAG^{+1})* and *CS-P3(ALT:FLAG^{+1})* RNAs. For P3N’ and P3N-PIPO’, the C-terminal regions are different among the virus constructs. P3 and P3N sequences are shown in green, PIPO sequences are in blue (underlined), ALT sequences are in magenta (underlined), and the FLAG-tag sequence is shown in black (bold).

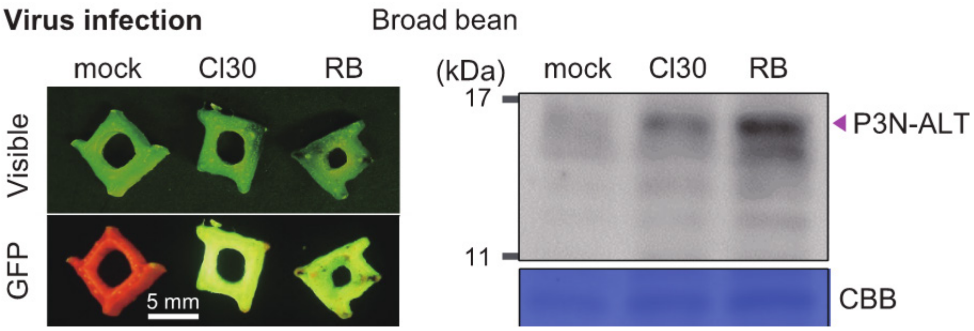

Supplementary Figure 3. Detection of P3N-ALT accumulation in ClYVV-infected broad bean stems. The stem tissues systemically infected with Cl30 and RB as in Fig. 3 were harvested at 10 days post-inoculation. The infected areas were confirmed by virus-derived GFP fluorescence (left panel). P3N-ALT was detected by western blotting using a polyclonal antibody raised against the N-terminal region of P3. CBB-stained gel image is shown at the bottom of the western blot panel as a loading control. The position of P3N-ALT is marked with an arrowhead. Positions of molecular mass markers (kDa) are indicated on the left side of the panel.

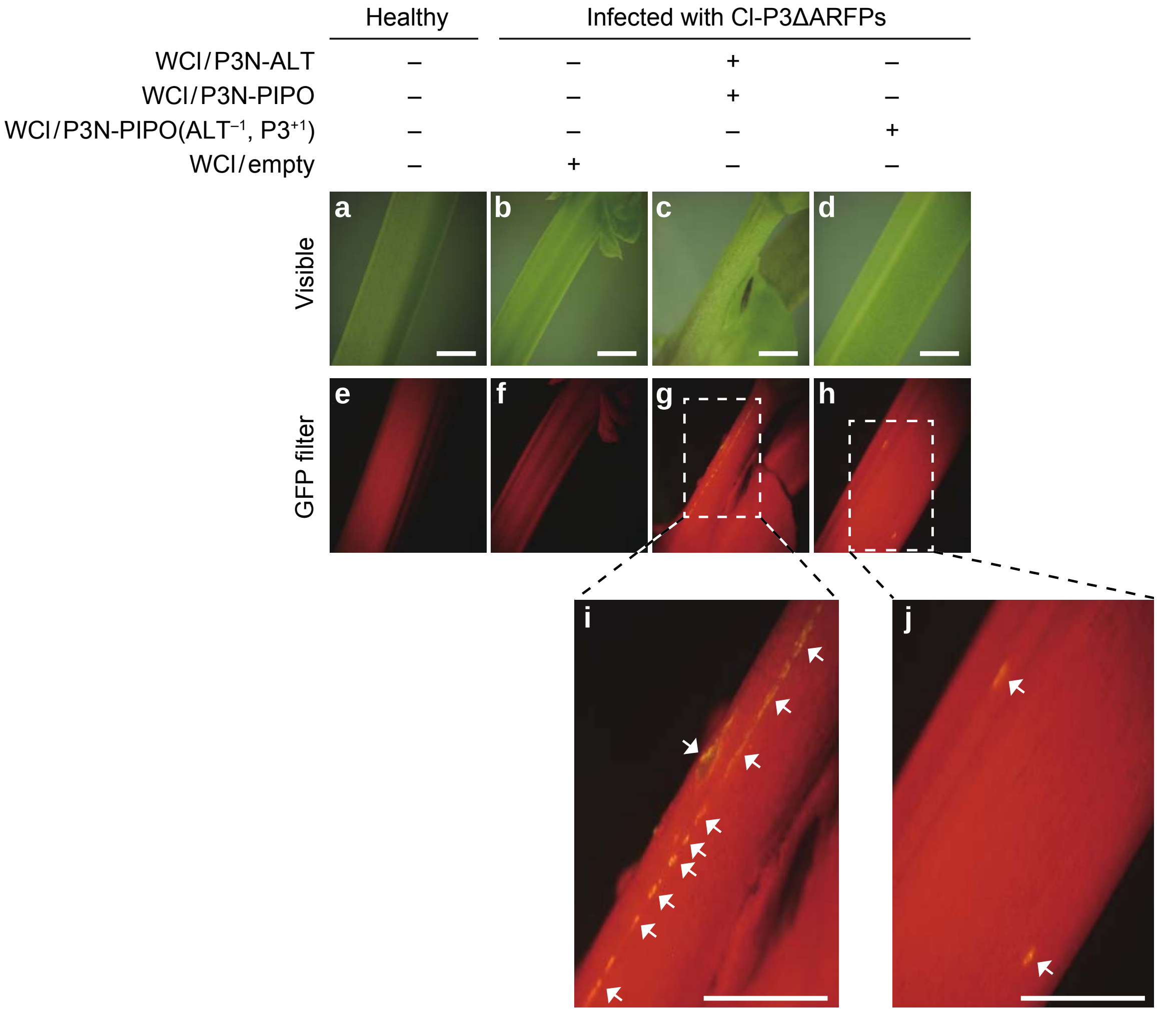

Supplementary Figure 4. Systemic infection of broad bean plants with ClYVV requires P3N-ALT and P3N-PIPO. Broad bean leaves were biolistically co-inoculated with Cl-P3ΔARFPs (Fig.4a) and WClMV vectors, WCl/P3N-ALT expressing P3N-ALT, WCl/P3N-PIPO expressing P3N-PIPO, WCl/P3N-PIPO(ALT^{-1}, P3^{+1}) expressing both P3N-PIPO and P3N-ALT, or an empty vector WCl/empty as indicated. Images of a healthy plant (**a**, **e**) are shown for comparison. Systemic movement of Cl-P3ΔARFPs was examined by monitoring GFP fluorescence derived from Cl-P3ΔARFPs. Visible images (**a**–**d**) and GFP fluorescence images in stems at 7 or 8 days post-inoculation (**e**–**j**) are shown. When co-inoculated with Cl-P3ΔARFPs and WCl/empty, no GFP signal was detected in any of the non-inoculated parts of the plants (**f**), whereas sporadic GFP signals (arrows) were detected in systemic stems of plants that were co-inoculated with WCl/P3N-ALT plus WCl/P3N-PIPO (**g**, **i**) or WCl/P3N-PIPO(ALT^{-1}, P3^{+1}) (**h**, **j**). This suggests that expression of P3N-ALT and P3N-PIPO *in trans* using WClMV vectors at least partly compensated for the defect of Cl-P3ΔARFPs to enable systemic spread of the virus in the inoculated broad bean plants. The test was repeated twice, and representative images are shown. Scale bar = 5 mm.

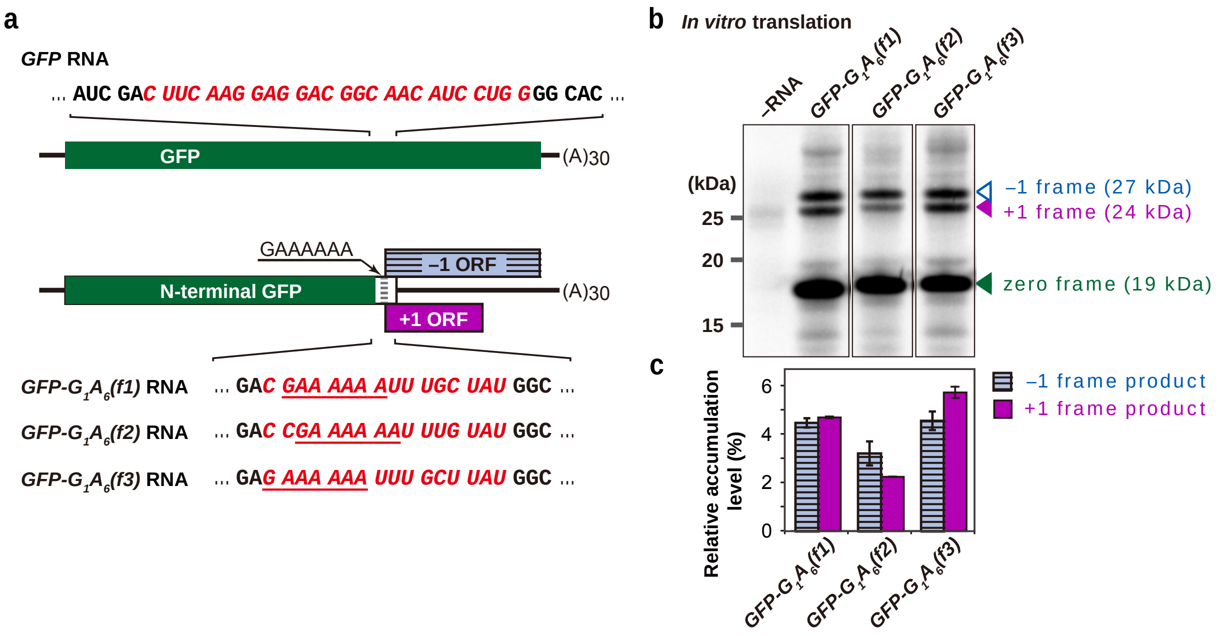

Supplementary Figure 5. Alternative reading frame proteins are produced regardless of the original reading frame *in vitro*. (**a**) Schematic diagram of RNA used for *in vitro* translation analysis. The nucleotide sequences around the G_2A_6 motif derived from RB were replaced with *GFP* RNA sequence (red italic letters) in three different reading frames to generate *GFP-G$_1$A$_6$(f1)*, *GFP-G$_1$A$_6$(f2)* and *GFP-G$_1$A$_6$(f3)* RNAs. The replaced sequences are marked in red italic letters. Spaces denote the zero reading frame. The G_2A_6 motif is underlined. (**b**) *In vitro* translation analysis using WGE. RNA prepared *in vitro* were translated in the presence of [^{35}S]methionine. The translation products were separated by SDS-PAGE, and the signals were visualized by autoradiography. The positions of zero frame, +1 frame, and –1 frame products are marked on the right side of the panel. Positions of molecular mass markers (kDa) are indicated on the left. (**c**) The accumulated levels of –1 and +1 frame proteins produced in each construct. In the calculation, the number of methionine codons present in each product was taken into account. The value for each product was obtained from at least two independent translation assays. The means and standard deviations are shown.

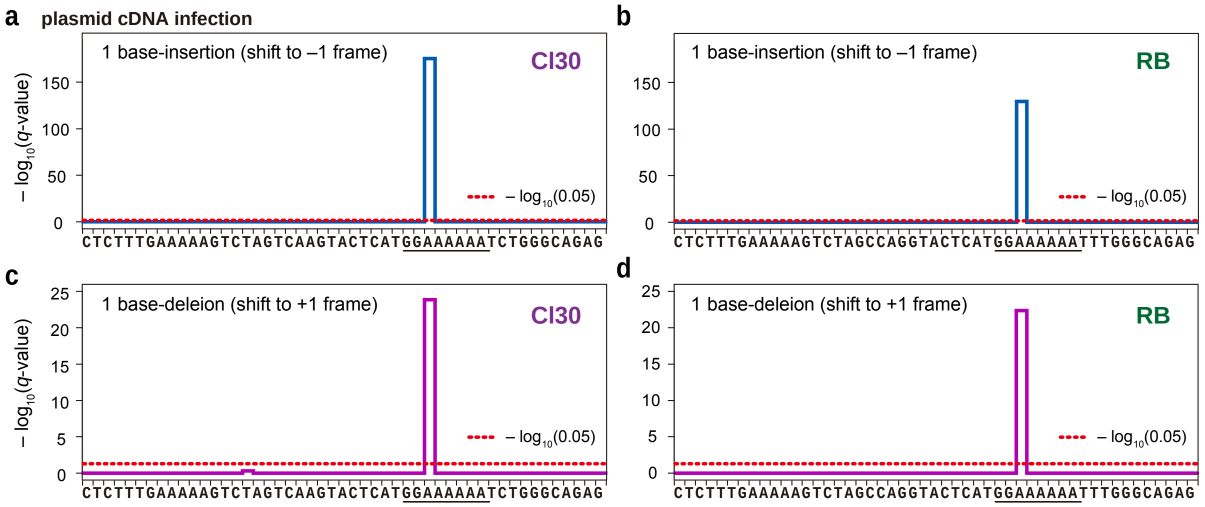

Supplementary Figure 6. Results of statistical tests for enrichment of indels in systemically infected viral cDNA. (**a**–**d**) Solid lines indicate *q*-values for enrichment of one base insertion (**a**,**b**) and one base deletion (**c**,**d**) in viral cDNAs from Cl30 (**a**,**c**) and RB (**b**,**d**), which are shown in Fig. 5b,c. Fisher's exact test was performed for the indel counts observed at each position in the amplicons from systemically infected viral RNA and control plasmids used for inoculation (Supplementary Methods). The *q*-values were obtained by multiple test correction of *p*-values calculated by Fisher's exact test using Storey's method. The vertical axis represents negative $\log_{10}$-transformed *q*-values. The red dashed lines indicate a statistical cut-off (q-value = 0.05).

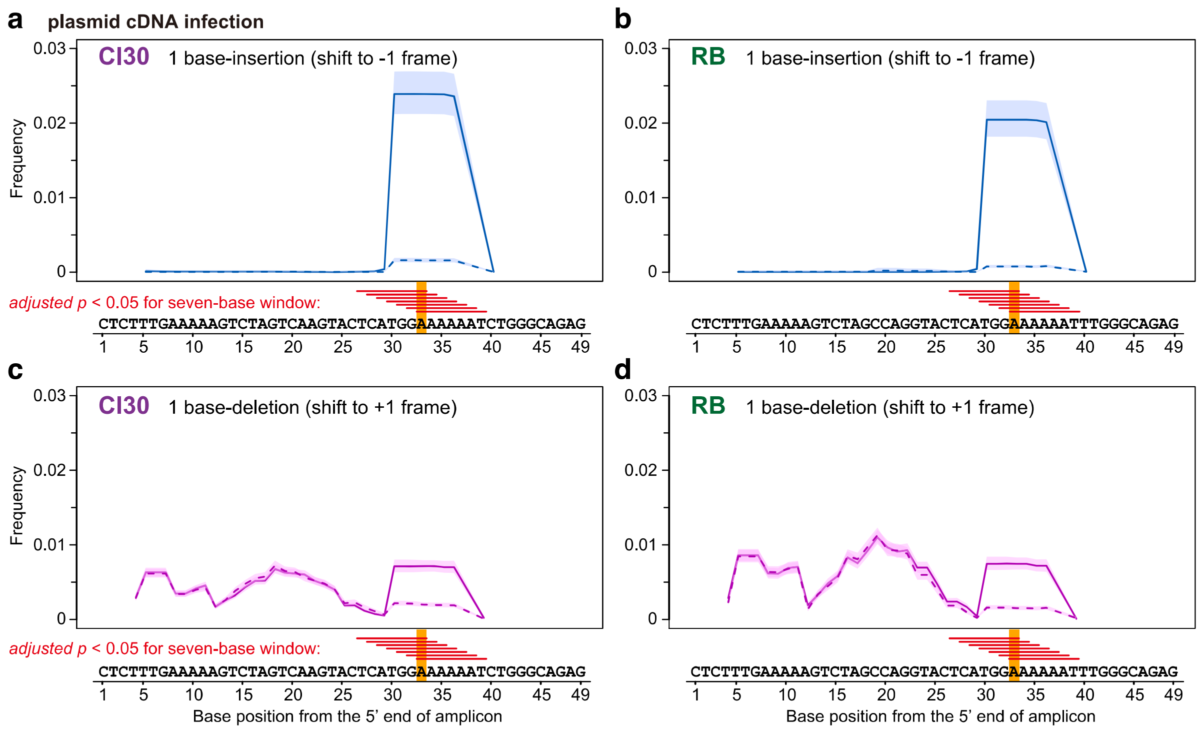

Supplementary Figure 7. Frequency of transcriptional slippage at ClYVV P3 region estimated using a negative binomial regression. Frequencies of one base insertions in Cl30 (**a**) and RB (**b**), and that of one base deletions in Cl30 (**c**) and RB (**d**), are shown. Sites and distribution of indels at the ClYVV P3 region were measured by amplicon sequencing of cDNA prepared from total RNA of Cl30 or RB-infected broad bean leaves. As controls, plasmids used for inoculation were subjected to sequencing. The frequency of insertions and deletions per base was estimated using a negative binomial regression as a ratio to the total number of reads obtained from respective samples. Solid and dashed lines indicate estimated indel frequencies in systemically propagated viral sample and inoculated plasmid sample, respectively. The indicated frequency was estimated in a window size of seven bases (Supplementary Table 2), with a step size of one base. Standard errors of the estimated frequencies are shown as shaded area. Numbers at the bottom indicate the position in the amplicons (numbered from the 5′ end). Seven base-windows in which indel frequencies are statistically significantly different between viral RNA and control plasmid are shown with red bars under the plot (adjusted $p < 0.05$). The adjusted p-values were obtained by multiple test correction using Holm's method. The 33rd A, which is the common site among all the significant windows is highlighted by orange bars. Estimated indel frequencies obtained using different window sizes are shown in Supplementary Table 2.

Supplementary Figure 8. Alignment of the P3N-ALT amino acid sequences of potyviruses. Potyviral ORF sequences were retrieved from GenBank using the queries "potyvirus" and "complete genome". These entries were further filtered by selecting for those with annotation for the *P3* cistron. The *P3* cistron sequences of the resultant 197 potyvirus genomes were obtained and one base deletion at the $G_{1–2}A_{6+}$ or G_0A_{6+} motif was simulated using a custom Perl script. The simulated sequences were aligned using Muscle v3.8.31 software. For those entries that gave identical P3N-ALT amino acid sequence, only one entry is shown. These include (the omitted Accession Nos. are listed in the parentheses), plum pox virus: AB576046 (AB576047, AB576048, AB576049, AB576050, AB576051, AB576052, AB576054, AB576055, AB576056, AB576057, AB576058, AB576059, AB576061, AB576062, AB576063, AB576064, AB576065, AB576066, AB576067, AB576068, AB576069, AB576070, AB576073, AB576074, AB576075, AB576076, AB576077, AB576078, AB576079, AB576080); potato virus Y: AB711153 (AB711154, AB711155, AB714134); AB711143 (AB711152); AB711147 (AB711148); AB711145 (AB711146); bean yellow mosaic virus: AB079886 (AB079887); konjac mosaic virus: NC_007913 (AB219545); papaya leaf distortion mosaic virus: AB088221 (NC_005028); soybean mosaic virus: AY294044 (AJ312439); zucchini yellow mosaic virus: AB188115 (AB188116); Algerian watermelon mosaic virus: EU410442 (NC_010736); Moroccan watermelon mosaic virus: EF579955 (NC_009995); sugarcane mosaic virus: JX237862 (JX237863); peanut mottle virus: AF023848 (NC_002600); turnip mosaic virus: AB701740 (AB701741); AB701702 (AB701728, AB701731, AB701732); AB701700 (AB701719); AB701712 (AB701714); AB194790 (AB194791); AB194785 (AB194786, AB194787, AB194788, AB194789, AB194792, AB194793, AB194794, AB194795, AB194796, AB194797, AB194798, AB194799, AB194800, AB194801, AB194802); AB701733 (AB701738); AB701734 (AB701735); AB701690 (AB701691); Habenaria mosaic virus: AB818538 (NC_021786); chilli veinal mottle virus: AJ972878 (AM909717). As a result, 125 entries are shown. Lupine mosaic virus (LuMV; Accession No. NC_014898) was recently isolated[68] and is distantly related to other potyviruses[49]. LuMV does not carry the $G_{1–2}A_{6–7}$ motif[67], but carries G_0A_6 within the *P3* cistron at a similar position to other potyviruses. Amino acid sequences of the ALT region were aligned manually. LuMV was placed at the bottom of the figure. Number of amino acid residues from the N-terminus of P3N is indicated. The amino acid sequence EK, which is highlighted in yellow, is of the expected slippage site of $G_{1–2}A_{6–7}$ motif in P3. P3N and ALT regions are marked in green and magenta, respectively.

Accession	P3N		Viral species
AB576053	G-----------------LEVD-KCDEFKN--VKLLIRSIYKPQIMEQVLKEEPYLLLMSVLSPGV	46	plum pox virus
AB576046	G-----------------LEVD-KCDEFKN--VKLLIRSIYKPQIMEQVLKEEPYLLLMSVLSPGV	46	plum pox virus
AB576072	G-----------------LEVD-RCDEFKN--VKLLIRSIYKPQIMEQVLKEEPYLLLMSVLSPGV	46	plum pox virus
AB576071	G-----------------LEVD-RCDEFKN--VKLLIRSIYKPQIMEQVLKEEPYLLLMSVLSPGV	46	plum pox virus
AB545926	G-----------------LEVD-KCDEFKN--VKLLIRSIYKPQIMEQVLKEEPYLLLMSVLSPGV	46	plum pox virus
AB576045	G-----------------LEVD-KCDEFKN--VKLLIRSIYKPQIMEQVLKEEPYLLLMSVLSPGV	46	plum pox virus
AB576060	G-----------------LEVD-KCDEFKN--VKLLIRSIYKPQIMEQVLKEEPYLLLMSVLSPGV	46	plum pox virus
NC_001445	G-----------------LEVD-KCDEFKN--VKLLIRSIYKPQIMEQVLKEEPYLLLMSVLSPGV	46	plum pox virus
NC_004752	G-----------------LVHH-SEHDSRS--VKLLIKSVYKPKLMEQVLMEEPYLLVFAMVSPCV	46	yam mosaic virus
AB711144	G-IPGACPELGSTISPFREGGI-IMSESAA--LKLLLKGIFRPKVMRQLLLDEPYLLILSILSPGI	62	potato virus y
AB714135	G-IPGACPELGSTISPFREGGI-IMSESAA--LKLLLKGIFRPKVMRQLLLDEPYLLILSILSPGI	62	potato virus y
AB711153	G-VPNACPELGSTISPFREGGV-IMSESAA--LKLLLKGIFRPKVMRQLLLDEPYLLILSILSPGI	62	potato virus y
NC_001616	G-VPNASPELGSTISPFREGGV-IMSESAA--LKLLLKGIFRPKVMRQLLLDEPYLLILSILSPGI	62	potato virus y
AM113988	G-VPNACPELGSTISPFREGGV-IMSESAA--LKLLLKGIFRPKVMRQLLLDEPYLLILSILSPGI	62	potato virus y
AB711143	G-IPNACPELGSTISPFREGGV-IMSESAA--LKLLLKGIFRPKVMRQLLLDEPYLLILSILSPGI	62	potato virus y
AB711151	G-VPNACPELGSTISPFREGGV-IMSESAA--LKLLLKGIFRPKVMRQLLLDEPYLLILSILSPGI	62	potato virus y
AB711149	G-VPNACPELGSTISPFREGGV-IMSESAA--LKLLLKGIFRPKVMRQLLLDEPYLLILSILSPGI	62	potato virus y
AB711147	G-VPNACPELGSTISPFREGGV-IMSESAA--LKLLLKGIFRPRVMRQLLLDEPYLLILSILSPGI	62	potato virus y
AB711145	G-IPNACPELGSTISPFREGGV-IMSESAA--LKLLLKGIFRPKVMRQLLLDEPYLLILSILSPGI	62	potato virus y
AB711150	G-VPNACPELGSTISPFREGGV-IMSESAA--LKLLLKGIFRPKVMRQLLLDEPYLLILSILSPGI	62	potato virus y
FJ214726	G-IPSACPELGSTRSPFREGGV-IMSESEA--LKLLLKGIFRPKVMRQLLLDEPYLLVLSILSPGI	62	potato virus y
AB126033	G----TVENHKVKIDDQPGRCG--VSEFHA--IRMLIKGIYRPSVMYELLSEEPYLLVFSILSPSI	58	pepper mottle virus
AF501591	GIVENHKVQIDS-----QPSRC-GVSEFHA--IRMLIKGIYRPSVMYELLSEEPYLLVFSILSPSI	58	pepper mottle virus
NC_001517	G------IVENHKVQIDNQPSRCGVSEFHA--IRMLIKGIYRPSVMYELLSEEPYLLVFSILSPSI	58	pepper mottle virus
AB570195	G-------------------KK-FTVEYEA--VKLLIKAIYRPKLMRTLMEDEPYLICLAMCSPGV	44	lily mottle virus
AM048875	G-------------------RK-FTVEYEA--VKLLIKAIYRPKLMRTLMEDEPYLICLAMCSPGV	44	lily mottle virus
JN127341	G-------------------QK-FTVEYEA--VKLLIKAIYRPKLMRTLLEDEPYLICLAMCSPGV	44	lily mottle virus
NC_003492	G------------------DWN-FGNRVRT-DTKFLLKSIYRPDLLERIIEHEPFVLVLAMQSPAV	46	bean yellow mosaic virus
AB079888	G------------------DWN-FGNRVRT-DTKFLLKSIYRPDLLERIIEHEPFVLVLAMQSPAV	46	bean yellow mosaic virus
AB079886	G------------------DYN-FGSRVRI-DTKFLLKSIYRPDLLERIIEHEPFVLVLAMQSPAV	46	bean yellow mosaic virus
**AB011819**	G-----------------KSLT-GQVIQFD--TKMLISSIYRPRQMEKIINEEPFVLVLAMQSPSV	46	**clover yellow vein virus**
NC_007913	G-----------------TANQ-LLMPQNA--FSLVIKGIYRPEVMLQILEEEPYLLMMTLLSPGV	46	konjac mosaic virus
AJ885005	G-----------------KFVA-SHLVMTN--FNTLIKAIYRADLMRDILTHEPYLVMLAVLSPGV	46	thunberg fritillary virus
AB088221	G-----------------DLHS-KQAPQCS--IKLLCKCIYRPKLMRQCIEEEPFLLILACISPGV	46	papaya leaf distortion mosaic virus
NC_001768	G-----------------LVYS-ENNDASA--VKALTQAIFRPDVLSELIEKEPYLMVFALVSPGI	46	tobacco vein mottling virus
AJ131401	G-----------------TSNS-QINEYAT--IKMLAKAVYRPKLMKEIIHEQPYMLVMSLMSPGI	46	potato virus A
NC_004039	G-----------------TSNS-QINGYAT--IKMLAKAVYRPKLMKEIIHEQPYMLVMSLMSPGI	46	potato virus A
AJ131400	G-----------------TPNS-QINGYAT--IKMLAKAVYRPKLMREIIHEQPYMLVMSLMSPGI	46	potato virus A
AJ131402	G-----------------TPNS-QINGYAT--IKMLAKAVYRPKLMKEIIHEQPYMLVMSLMSPGI	46	potato virus A
AJ131403	G-----------------TSNS-QINGYST--IKMLAKAVYRPKLMREIIHEQPYMLVMSLMSPGV	46	potato virus A
NC_007728	G--------------------M-VEQRMKC--ETALISSIFKPKRMIQILEDDPYILLLGMISPSV	43	east Asian passiflora virus
AB604610	G--------------------E-VEQRMRC--ETALISSIFKPRRMIQLLNEDPYILLLGMVSPAV	43	east Asian passiflora virus
DQ399708	G--------------------E-AQQRMKC--ETALIKSIFKPKRLIQILEDDPYILLMGLISPSI	43	watermelon mosaic virus
AB218280	G--------------------E-AQQRMKC--ETALIKSIFKPKRMIQILEDDPYILLMGLISPSI	43	watermelon mosaic virus
KC292915	G--------------------V-AQQRMKC--ETALITSIFKPKRMIHILEEDPYILLMGLISPSI	43	watermelon mosaic virus
AY294044	G--------------------E-VQQRMKC--ETALITSIFKPKRMVQILENDPYILLMGLVSPSI	43	soybean mosaic virus
D00507	G--------------------E-VQQRMKC--ETALITSIFKPKRMIQILENDPYILLMGLVSPSI	43	soybean mosaic virus
AY294045	G--------------------E-VQQRMKC--ETALITSIFKPKRMVQILENDPYILLMGLVSPSI	43	soybean mosaic virus
AY216010	G--------------------E-VQQRMKC--ETALITSIFKPKIMVQILENDPYVLLMGLVSPSI	43	soybean mosaic virus
AY216987	G--------------------E-VQQRMKC--ETALITSIFKPKIMVQILENDPYVLLMGLVSPSI	43	soybean mosaic virus
AY278998	G--------------------T-PTQRIKL--EEQLIKGIFKPKLMMQLLHDDPYILLLGMISPTI	43	zucchini yellow mosaic virus
AB188115	G--------------------T-PTQHIKL--EEQLIKGIFKPKLMMQLLHDDPYILLLGMISPTI	43	zucchini yellow mosaic virus
AY278999	G--------------------A-PTQRIKL--EEQLIKGIFKPKLMMQLLHDDPYILLLGMISPTI	43	zucchini yellow mosaic virus
AY279000	G--------------------A-PTQRIKL--EEQLIKGIFKPKLMMQLLHDDPYILLLGMISPTI	43	zucchini yellow mosaic virus
AF014811	G--------------------T-PTQRIRL--EEQLIKGIFKPKIMMQLLHDDPYILLLGMISPTI	43	zucchini yellow mosaic virus
EU410442	G------------------------TTTHY--LGKLIKSVYRPNELMELLLNEPYIVALSLSSPSL	40	Algerian watermelon mosaic virus
EF579955	G------------------NPD-EAPVMPD--IFKIIKCVYKQNDMCKLLFDEPYIAVVALHSPAV	45	Moroccan watermelon mosaic virus
AJ278405	G------------------TLT-QQTFNTL--LKSLTKNMFKPERVKQIIEEEPFLLMMAVASPTV	45	sugarcane mosaic virus
JX237862	G------------------TLT-QQTFNTL--LKSLTKNMFKPERIKQIIEEEPFLLMMAVASPTV	45	sugarcane mosaic virus
AJ297628	G------------------TLT-QQTFNTL--LKTLTKNMFKPNKIKQIIEEEPFLLMMAIAPPTV	45	sugarcane mosaic virus
U57358	G------------------TLT-HNTFSSL--MKLLIKNMFKPHEMKKIIEEEPFLVMMAVTSPTV	45	sorghum mosaic virus
NC_003377	G------------------TIT-HKSFSTL--ISHLIKNMFKPREMRKIIEEEPFLIMLSVVSPTV	45	maize dwarf mosaic virus
AB465608	G-----------------VTAL-PDIQERC--IRTIIKGVYKPDVMYTILSEDPYALLLSVMSPRI	46	sweet potato feathery mottle virus
AB439206	G-----------------VTAL-PDIQERC--IRTIIKGVYKPDVMYTILSEDPYALLLSVMSPRI	46	sweet potato feathery mottle virus
NC_001841	G-----------------VTAL-PDVQERC--IRTIIKGVYKPDVMYTILSEDPYALLLSVMSPRI	46	sweet potato feathery mottle virus
NC_001671	G--------------TQKMEINMCCCQRKNLLIKQLIRAIYRPKLLTEIIETEPFVLMLAIVSPSI	52	pea seedrne mosaic virusrus
EU119422	G------------------TLE-NLSVMKA--VQVLIKSLWRPKLMNLVLTQEPFLIALAVVSPSV	45	cocksfoot streak virus
GU214748	G------------------DAN-SGRRMRM--ERALIKGIFRPKLLMHIIHEDPYTLMMSLLSPCM	45	freesia mosaic virus
AF023848	G------------------EAP-HARRMRM--EKALIQGIFKPKQLVYLIEEDPYILMMSLVSPTL	45	peanut mottle virus
NC_000947	G----------PILNNEDIDPTEYRTPSWH--LRRLIKGIYNPQVLLDDIRIDRYLPLYALLSPGV	54	Japanese yam mosaic virus
NC_011541	G----------DPLAEETTEQI-LSDPNWN--LQLLIQGIYKPRVMQENLIWNRYLPLYAMLSPGI	53	narcissus yellow stripe virus
AB701695	G----------TKWEDAHGTSN-IDNPQWC--IKRLIKGVYRPKQLKEDMLTNPFLPLYALLSPGV	53	turnip mosaic virus
AB701694	G----------TKWEDAHGTIN-IDNPQWC--IKRLIKGVYRPKQLKEDMLTNPFLPLYALLSPGV	53	turnip mosaic virus
AB701737	G----------TKWEDAHGANN-IDNPQWC--IRRLIKGVYRPKQLKEDMLTNPFLPLYALLSPGV	53	turnip mosaic virus
AB701720	G----------TKWEDAHGANN-IDNPQWC--IRRLIKGVYRPKQLKEDMLTNPFLPLYALLSPGV	53	turnip mosaic virus
AB701726	G----------TKWEDAHGASN-IDNPQWC--IRRLIKGVYRPKQLKEDMLTNPFLPLYALLSPGV	53	turnip mosaic virus
AB701705	G----------TKWEDAHGTSN-IDNPQWC--IRRLIKGVYRPKQLKEDMLTNPFLPLYALLSPGV	53	turnip mosaic virus
AB701696	G----------TKWKDTHGVNN-IDNPQWC--IRRLIKGVYRPKQLKEDMLLNPFLPLYALLSPGV	53	turnip mosaic virus
AB701706	G----------TKWEDAHGASN-IDNPQWC--IKRLIKGVYRPKQLKEDMLTNPFLPLYALLSPGV	53	turnip mosaic virus
AB701707	G----------TKWEEAHGASN-IDNPQWC--IKRLIKGVYRPKQLKEDMLTNPFLPLYALLSPGV	53	turnip mosaic virus
AB701721	G----------TKWEDAHGASN-IDNPQWC--IKRLIKGVYRPKQLKEDMMANPFLPLYALLSPGV	53	turnip mosaic virus
AB701697	G----------TKWEDTHGVSN-IDNPQWC--IRRLIKGVYRPKQLREDMLTNPFLPLYALLSPGV	53	turnip mosaic virus
AB701725	G----------TKWEDEHGANN-IDNPQWC--IKRLIKGVYKPKLLKQDMLTNPYLPLYALLSPGV	53	turnip mosaic virus
AB701701	G----------TKWEDEHGFDN-IDDPQWC--IKRLIKGVYRPKLLKQDMLLNPFLPLYALLSPGV	53	turnip mosaic virus
AB701740	G----------TKWEDEHGFDN-IDDPQWC--IKRLIKGVYRPKLLKQDMLLNPFLPLYALLSPGV	53	turnip mosaic virus
AB701702	G----------TEWENAHGADN-IDNPQWC--IKRLVKGVYRPKQLKEDMLANPFLPLYALLSPGV	53	turnip mosaic virus
AB701710	G----------TEWENVHGADN-IDNPQWC--IKRLVKGVYRPKQLKEDMLANPFLPLYALLSPGV	53	turnip mosaic virus
AB701736	G----------TEWENAHGADN-IDNPQWC--IKRLVKGVYRPKQLKEDMLANPFLPLYALLSPGV	53	turnip mosaic virus
AB701708	G----------TEWENAHGADN-IDNPQWC--IKRLVKGVYRPKQLKEDMLANPFLPLYALLSPGV	53	turnip mosaic virus
AB701703	G----------TEWENAHGADN-IDNPQWC--IKRLVKGVYRPKQLKEDMLLNPFLPLYALLSPGV	53	turnip mosaic virus
AB701699	G----------TEWENAHGADN-IDNPQWC--IKRLVKGVYRPKQLKEDMLANPFLPLYALLSPGV	53	turnip mosaic virus
AB701729	G----------TEWENAHGAEN-IDNPQWC--IKRLVKGVYRPKQLKEDMLANPFLPLYALLSPGV	53	turnip mosaic virus
AB701716	G----------TEWEDAHGVDN-IDNPQWC--IKRLVKGVYRPKQLKEDMLANPFLPLYALLSPGV	53	turnip mosaic virus
AB701717	G----------TEWEDAHGVDN-IDNPQWC--IKRLVKGVYRPKQLKEDMLANPFLPLYALLSPGV	53	turnip mosaic virus
AB701739	G----------TDWENAHGAGN-IDDPQWC--IRRLVKGVYRPKQLKEDMLANPFLPLYALLSPGV	53	turnip mosaic virus
AB701700	G----------TEWENAHGADN-IDDPQWC--IKRLVKGVYKPKQLKEDMLANPFLPLYALLSPGV	53	turnip mosaic virus
AB701727	G----------TEWEDTHGASN-IDNPQWC--IKRLIKGVYRPKQLKEDMLANPFLPLYALLSPGV	53	turnip mosaic virus
AB701742	G----------TEWEDTHGVSN-IDNPQWC--IKRLIKGVYRPKQLKEDMLANPFLPLYALLSPGV	53	turnip mosaic virus
AB701711	G----------TEWEDTHGASN-IDNPQWC--IKRLIKGVYRPKQLKEDMLANPFLPLYALLSPGV	53	turnip mosaic virus
AB362512	G----------TEWEDTHGASN-IDNPQWC--IKRLIKGVYRPKQLKEDMLANPFLPLYALLSPGV	53	turnip mosaic virus
AB701712	G----------TEWEDTHGASN-IDNPQWC--IKRLIKGVYRPKQLKEDMLANPFLPLYALLSPGV	53	turnip mosaic virus
AB701718	G----------TEWEDTHGASN-IDNPQWC--IKRLIKGVYRPKQLKEDMLANPFLPLYALLSPGV	53	turnip mosaic virus
AB701715	G----------TEWEDTHGASN-IDNPQWC--IKRLIKGVYRPKQLREDMLANPFLPLYALLSPGV	53	turnip mosaic virus
AB701713	G----------TEWEDTHGASN-IDNPQWC--IKRLIKGVYRPKQLREDMLANPFLPLYALLSPGV	53	turnip mosaic virus
AB194790	G----------TEWEDTHGSSN-IDNPQWC--IKRLIKGVYKPKQLKEDMLANPFLPLYALLSPGV	53	turnip mosaic virus
AB194785	G----------TEWEDTHGSSN-IDNPQWC--IKRLIKGVYKPKQLKEDMLANPFLPLYALLSPGV	53	turnip mosaic virus
AB701730	G----------TEWEDTHGASN-IDNPQWC--IKRLIKGVYRPKQLKEDMLANPFLPLYALLSPGV	53	turnip mosaic virus
AB701733	G----------TEWEDTHGASN-IDNPQWC--IKRLIRGVYRPKQLKEDMLANPFLPLYALLSPGV	53	turnip mosaic virus
AB701709	G----------TEWEDTHGANN-IDDPQWC--IKRLIKGVYRPKQLKEDMLANPFLPLYALLSPGV	53	turnip mosaic virus
AB701704	G----------TEWEDAHGASN-IDDPQWC--IKRLIKGVYRPKQLKEDMLANPFLPLYALLSPGV	53	turnip mosaic virus
AB362513	G----------TAWEEAHGFNN-IDDPQWC--IKRLIKGVYRPKQLKEDMLANPFLPLYALLSPGV	53	turnip mosaic virus
AB701722	G----------TKWEDVHGTKN-IDDPQWC--IKRLVKGVYRPKQLKEDMLANPFLPLYALLSPGV	53	turnip mosaic virus
AB701723	G----------TKWEDVHGTKN-IDDPQWC--IKRLVKGVYRPKQLKEDMLANPFLPLYALLSPGV	53	turnip mosaic virus
AB701724	G----------TKWEEIHGTKN-IDDPQWC--IKRLVKGVYRPKQLKEDMLANPFLPLYALLSPGV	53	turnip mosaic virus
AB440238	G----------TKWEDVHGAKN-IDDPQWC--IKRLVKGVYRPKQLKEDMLANPFLPLYALLSPGV	53	turnip mosaic virus
AB440239	G----------TEWEDVHGAKN-IDDPQWC--IKRLVKGVYRPKQLKEDMLANPFLPLYALLSPGV	53	turnip mosaic virus
AB701734	G----------TRWEDIHGAQN-VDDPQWC--IRRLIKGVYRPKQLKEDMLANPFLPLYALLSPGV	53	turnip mosaic virus
AB701698	G----------TKWADTHGATN-IDNPQWC--IKRLIKGVYRPKQLKADMLENPFLPLYALLSPGV	53	turnip mosaic virus
AB701690	G----------TILEDAYGAEN-IADPQWN--IKRLIQGIYKPKVLKEDMLLNPYLPLYALLSPGV	53	turnip mosaic virus
AB701692	G----------TILEDAYGAEN-IADPQWN--IKRLIQGIYKPKVLKEDMLLNPYLPLYALLSPGV	53	turnip mosaic virus
AB701693	G----------TILEDAYGAGN-IADPQWN--IKRLIQGIYKPKVLKEDMLLNPYLPLYALLSPGV	53	turnip mosaic virus
AB818538	G-----------------TNYNGTDFHTRS--LKQVIRGVYRPNELRSILSHDPYILTMALLSPAI	47	Habenaria mosaic virus
NC_005778	G-----------------EETQ-SIPGFKT----RLMRSVYRPSVFEQLMIDEPLTLTLALASPTL	44	chilli veinal mottle virus
AJ972878	G-----------------EEMQ-SLPGFKT----RLMRSVYRPSVFEQLMIDEPLTLTLALASPTL	44	chilli veinal mottle virus
NC_014898	G---------RTDNLDPSALGN-GNNRTNI--MSQLIRSIYRPNEMHRMLTEHPLYIVYAMLSPGV	54	lupine mosaic virus

Supplementary Figure 8 (part 1/3)

Accession	P3N		Viral species
AB576053	LMALFNSGSLEKATQYWITRSHSLAAITSMLSALAAKVSLASTLNAQMSVIDEHAAVLCDSVFDGT	112	plum pox virus
AB576046	LMALFNSGSLEKATQYWITRSHSLAAITSMLSALAAKVSLASTLNAQMSVIDEHAAVLCDSVFDGT	112	plum pox virus
AB576072	LMALFNSGSLEKATQYWITRSHSLAAITSMLSALAAKVSLASTLNAQMSVIDEHAAVLCDSVFDGT	112	plum pox virus
AB576071	LMALFNSGSLEKATQYWITRSHSLAAITSMLSALAAKVSLASTLNAQMSVIDEHAAVLCDSVFDGT	112	plum pox virus
AB545926	LMALFNSGSLEKATQYWITRSHSLAAITSMLSALAAKVSLASTLNAQMSVIDKHAAVLCDSVFDGT	112	plum pox virus
AB576045	LMALFNSGSLEKATQYWITRSHSLAAITSMLSALAAKVSLASTLNAQMXVIDEHAAVLCDSVFDGT	112	plum pox virus
AB576060	LMALFNSGSLEKATQYWITRSHSLAAITSMLSTLAAKVSLASTLNAQMSVIDKHAAVMCDSVFDGT	112	plum pox virus
NC_001445	LMALFNSGSLEKATQYWITRSHSLAAITSMLSALAAKVSLASTLNAQMSVIDEHAAVLYDSVFVGT	112	plum pox virus
NC_004752	LMAMFNSGSLERATHFWLRQQDDLAFISSALSTLAQKVSTAHSLIDQLDIINEMASPMLEQIFVGS	112	yam mosaic virus
AB711144	LMAMYNNGIFELAVRLWINEKQSIAMIASLLSALALRVSAAETLVAQRIIIDTAATDLLDATCDGF	128	potato virus y
AB714135	LMAMYNNGVFELAVKLWINEKQSVAMIASLLSALALRVSAAETLVAQRIIIDTAATDLLDATCDGF	128	potato virus y
AB711153	LMAMYNNGIFELAVRLWINEKQSIAMIASLLSALALRVSAAETLVAQRIIIDAAATDLLDATCDGF	128	potato virus y
NC_001616	LMAMYNNGIFELAVRLWINEKQSIAMIASLLSALALRVSAAETLVAQRIIIDAAATDLLDATCDGF	128	potato virus y
AM113988	LMAMYNNGIFELAVRLWINEKQSIAMIASLLSALALRVSAAETLVAQRIIIDTAATDLLDATCDGF	128	potato virus y
AB711143	LMAMYNNGIFELAVRLWINEKQSIAMIASLLSALALRVSAAETLVAQRIIIDAAATDLLDATCDGF	128	potato virus y
AB711151	LMAMYNNGIFELAVRLWINEKQSIAMIASLLSALALRVSAAETLVAQRIIIDAAATDLLDATCDGF	128	potato virus y
AB711149	LMAMYNNGIFELAVKLWINEKQSIAMIASLLSALALRVSAAETLVAQRIIIDAAATDLLDATCDGF	128	potato virus y
AB711147	LMAMYNNGIFELAVRLWINEKQSIAMIASLLSALALRVSAAETLVAQRIIIDAAATDLLDATCDGF	128	potato virus y
AB711145	LMAMYNNGIFELAVRLWINEKQSIAMIASLLSALALRVSAAETLVAQRIIIDTAATDLLDATCDGF	128	potato virus y
AB711150	LMAMYNNGIFELAVRLWINEKQSIAMIASLLSALALRVSAAETLVAQRIIIDAAATDLLDATCDGF	128	potato virus y
FJ214726	LMAMYNNGIFELAVKLWINEKQSIAMIASLLSALALRVSAAETLVAQRIIMDAAATDLLDATCDGF	128	potato virus y
AB126033	LIAMYNDRAFELAVQIWLEKEQSIPLIATILTNLAAKVSVATTLVQQLQLIELSADQLLNVTCDGF	124	pepper mottle virus
AF501591	LIAMYNDRAFELAVQIWLEKEQSIPLIATILTNLAAKVSVATTLVQQLQLIELSADQLLNVTCDGF	124	pepper mottle virus
NC_001517	LIAMYNDRAFELAVQIWLEKEQSIPLIATILTNLAAKVSVATTLVQQLQLIELSADQLLNVTCDGF	124	pepper mottle virus
AB570195	LIALFNSGSLETAINYWIARHKDTAQIFALLTVLATKVSVARTLTEQLAILSDHATDMLKIMDRTF	110	lily mottle virus
AM048875	LIALFNSGSLETAINYWIARHKDTAQIFALLTVLATKVSVARTLTEQLAILSDHATDVLKIMDRTF	110	lily mottle virus
JN127341	LIALFNSGSLELAINYWIARHKDTAQIFALLTVLATKVSVARTLTEQLALLSDHATDVLKIMDRTF	110	lily mottle virus
NC_003492	LLALFNSASLEKAVQYWMHREMQVSHIMTLLAVLASNVSASRLLTTQFEIIEASAPQILAEMDKVH	112	bean yellow mosaic virus
AB079888	LLALFNSASLEKAVQYWMHREMQVSHIMTLLAVLASNVSASKLLTTQFEIIEASAPQILAEMDKVH	112	bean yellow mosaic virus
AB079886	LLALFNSASLEKAVQYWMHREMQVSHIMTLLAVLASNVSASKLLTTQFEIIEASAPQILAEMDKVH	112	bean yellow mosaic virus
**<u>AB011819</u>**	LLALFNSASLEKAVEVWLHKDMRVSHVMTMLALLAAKVSAAKMVNLQMEIIEASAGHFLAAMDTIH	112	**<u>clover yellow vein virus</u>**
NC_007913	MIALANSGSLERGMQMWIRRDKGFARMFVIIYTLAAKVTTARTLERQLNIIQETAPALFNEIFDGF	112	konjac mosaic virus
AJ885005	LLALFNSGSLEVATRMWIRQDQNIAQIMTMLSILAAKVSVSKVLLEQKRIIEMNAEHLLRATDHTF	112	thunberg fritillary virus
AB088221	LLALYNSQHLELALKYWMSKQQSVAALFAMIHGLAAKVTVAQTLNEQRLILERGARNLISVMETIH	112	papaya leaf distortion mosaic virus
NC_001768	LMAMSNSGALEFGISKWISSDHSLVRMASILKTLASKVSVADTLALQKHIMRQNANFLCGELINGF	112	tobacco vein mottling virus
AJ131401	LIALANSGALEMGIHHWIREGDSLVKMAHMLRTVAQNVSVARATWVQQEIISDSAQQMLETILNGT	112	potato virus A
NC_004039	LIALANSGALEMGIHHWIREGDSLVKMAHMLRTVAQNVSVARATWVQQEIISDSAQQMLETILNGT	112	potato virus A
AJ131400	LIALANSGALEMGIHHWIREGDSLVKMAHMLRTVAQNVSVARATWVQQEIISDSAQQMLETILNGT	112	potato virus A
AJ131402	LIALANSGALEMGIHHWIREGDSLVKMAHMLRTVAQNVSVARATWVQQEIISDSAQQMLETILNGT	112	potato virus A
AJ131403	LIALANSGALEMGIHHWIREGDSLVKMAHMLRTLAQNVSVARATWVQQEIISDSAQQMLEIILNGT	112	potato virus A
NC_007728	LIHMYRMRHFEKGIQTWISKDQNVTKIFIIMEQLTKKLAVNDILLDQLQIISGSSGHLLELLVDCP	109	east Asian passiflora virus
AB604610	LIHMYRMRHFEKGIQTWVHKDQNIAKIFIILEQLTKKVVLNDVLIDQLQVISGSSGHLLEVLSSCP	109	east Asian passiflora virus
DQ399708	LIHMYRMKHFEKSIELWISKEHSVAKIFIIMEQLTRKIAANDLLLEQLDIIAGTSQKLMDVLEDCP	109	watermelon mosaic virus
AB218280	LIHMYRMKHFEKGIELWISKEHSVAKIFIIMEQLTRKIAANDLLLEQLDIIAGTSQKLMDVLEDCP	109	watermelon mosaic virus
KC292915	LIHMYRMKHFEKGIEMWISKEHSVAKIFIIMEQLTRKIAANDLLLEQLNIIAGTSHKLMDVLEDCP	109	watermelon mosaic virus
AY294044	LIHMYRMKHFEKGVELWISKEHSVAKIFIILEQLTKRVAANDVLLEQLEMISETSERFMSILEDCP	109	soybean mosaic virus
D00507	LIHMYRMKHFEKGVELWISKEHSVAKIFIILEQLTKRVAANDVLLEQLEMISETSERFMSILEDCP	109	soybean mosaic virus
AY294045	LIHMYRMKHFEKGVELWISKEHSVAKIFIILEQLTKRVAANDVLLEQLEMISETSERFTSILEDCP	109	soybean mosaic virus
AY216010	LIHMYRMKHFEKGVELWISKEHSVAKIFIILEQLTKRVAANDVLLEQLEMISETSERFMSILEDCP	109	soybean mosaic virus
AY216987	LIHMYRMKHFEKGMELWISKEHSVAKIFIILEQLTKRVAANDVLLEQLEMISETSERFMSILEDCP	109	soybean mosaic virus
AY278998	LVHMYRMRHFERGIEIWIKRDHEIGKIFVILEQLTRKVALAEVLVDQLDLISEASPHLLEIMKGCQ	109	zucchini yellow mosaic virus
AB188115	LVHMYRMRHFERGIEIWIKRDHEIGKIFVILEQLTRKVALAEVLVDQLGLISEASPHLLEIMKGCQ	109	zucchini yellow mosaic virus
AY278999	LVHMYRMRHFERGIEIWIKRDHEIGKIFVILEQLTRKVALAEILVDQLDLISEASPHLLEILKGCQ	109	zucchini yellow mosaic virus
AY279000	LVHMYRMRHFERGIEIWIKRDHEIGKIFVILEQLTRKVALAEILVDQLDLISEASPHLLEILKGCQ	109	zucchini yellow mosaic virus
AF014811	LVHMYRMRHFERGIEIWIKRDHEIGKIFVILEQLTRKVALAEILVDQLDLISEASPHLLEIMNGCQ	109	zucchini yellow mosaic virus
EU410442	LLSLFNSGSLELALKHWIKRDQDVAEMIVLVESIAKRVTVARSLSDQFKAITINSRPLKLQLERDI	106	Algerian watermelon mosaic virus
EF579955	LIAMFNSGSLEVAIKYWIKRDQEVSEMFAMIETLAQKVSVARSIGDQFKEISINSRAIRQQLEAKI	111	Moroccan watermelon mosaic virus
AJ278405	LISLYNNCYIEQAMTYWIVKNQGIAAIFAQLEALAKKTSQAELLVLQMQILEKASNQLRLAVTGLN	111	sugarcane mosaic virus
JX237862	LISLYNNCYIEQAMTYWIVKNQGIAAIFAQLEALAKKTSQAELLVLQMQILEKASNQLRLAVTGLN	111	sugarcane mosaic virus
AJ297628	LISLYNNCYIEQAMTYWIVKNQGVAAIFAQLEALAKKTSQAELLVLQMQILEKASNQLRLAVTGLN	111	sugarcane mosaic virus
U57358	LVAMYNNCYLEQAMSYWIVKNQGVAAIFAQLEALAKETSQAELIVQQMSILEKASTQLRFAVSGIS	111	sorghum mosaic virus
NC_003377	LISLYNNCHIENAMAYWITKNQGVAAMFAQLEALAKETSKAELLIQQMTILEKASNQLKLAVMGLN	111	maize dwarf mosaic virus
AB465608	LLALLNSGSLDRSMEAWITEDQEVAVIIGTLQELAKKVSTSRVLEKQLKVIESQAHTLLFDPSLVR	112	sweet potato feathery mottle virus
AB439206	LLALLNSGSLDRSMEAWITEDQEVAVIIGTLQELAKKVSTSRVLEKQLKVIESQAHTLLFDPSLVR	112	sweet potato feathery mottle virus
NC_001841	LLALLNSGSLDRSMEAWITEDQEVAVIIGTLQELAKKVSTSRVLEKQLKVIESQAHTLLFDPAFVR	112	sweet potato feathery mottle virus
NC_001671	LKAMFRSGTFNQAIKFYMHRSKPTAQTLAFLEALSERVSRSRVLSEQFNIIDGALKELKSLANMSM	118	pea seedrne mosaic virusrus
EU119422	LIAMHTSGTFELAIERWLEDNQDIAVLFSTLNSLAEKVSRSRFLVDQMHMIEESCEELRRLAWDIK	111	cocksfoot streak virus
GU214748	LLNLYNVGGLEVAMREWIVKEASVSAIFATMSRMAESVSRADMVVEQLMVIRSHAGHFLELLASLN	111	freesia mosaic virus
AF023848	LINLFNVGGLEVAMKHWIKKEMNIGLIFSMLSSLAQKVSRADLVNEQITMIDANAAQFIETLAGID	111	peanut mottle virus
NC_000947	LIAMYNSASLEILTREYLRKDDEFVSIVLILESLARKVSVSTSLMSQLMLIEGEAQYIIEAVQGIK	120	Japanese yam mosaic virus
NC_011541	LLAFYNSGSLETMTKYFLQKDNDLVVLLVILESLATKVSKSRSVLAQLHILEKGASDVIEAVQNIK	119	narcissus yellow stripe virus
AB701695	ILAFYNSGSLEYLMNHYIRADSNVAVLLVVLKSLARKVSASQSVLAQLQVIERSLPELVEARANIT	119	turnip mosaic virus
AB701694	ILAFYNSGSLEYLMNHYIRADSNVAVLLVVLKSLARKVSTSQSVLAQLQVIERCLPELVEARANIT	119	turnip mosaic virus
AB701737	ILAFYNSGSLEYLMNHYIRADSNVAVLLVVLKSLARKVSASQSVLAQLQIIERSLPELVEARANVT	119	turnip mosaic virus
AB701720	ILAFYNSGSLEYLMNYYIKADSNTAVLLVVLKSLAKKVSASQSVLAQLQVIERSLPELVEARANIV	119	turnip mosaic virus
AB701726	ILAFYNSGSLEYLMNYYIKADSNTAVLLVVLKSLAKKVSASQSVLAQLQVIERSLPELVEARANIV	119	turnip mosaic virus
AB701705	ILAFYNSGSLEYLMNHYIKADSNTAVLLVVLKSLAKKVSASQSVLAQLQVIERSLPELVEARANIV	119	turnip mosaic virus
AB701696	ILAFYNSGSLEYLMNHYIKADSSVAVLLVVLKSLARKVSASQSVLAQLQVIERSLPELVEARANID	119	turnip mosaic virus
AB701706	ILAFYNSGSLEHLMNHYIRADSNVAVLLVVLKSLAKKVSTSQSVLAQLQIIERSLPELVEARANIN	119	turnip mosaic virus
AB701707	ILAFYNSGSLEHLMNHYIRADSNVAVLLVVLKSLAKKVSTSQSVLAQLQIIERSLPELVEARANIN	119	turnip mosaic virus
AB701721	ILAFYNSGSLEYLMNHYIKADSNIAVLLVVLKSLAKKVSVSQSVLAQLQVIERSLPELVEARANIT	119	turnip mosaic virus
AB701697	ILAFYNSGSLEYLMSHYIKADSNVAVLLVVLKSLAKKVSTSQSVLAQLQIIERSLPELVEARANIA	119	turnip mosaic virus
AB701725	ILAFYNSGSLEYLMNHYIRADSNVAVLLVVLKSLARKVSTSQSVLAQLQIIERSLPELVEARANVS	119	turnip mosaic virus
AB701701	ILAFYNSGSLEYLMNHYIRADSNVAVLLVVLKSLARKVSTSQSVLAQLQIIDRSLPELVEARANVI	119	turnip mosaic virus
AB701740	ILAFYNSGSLEYLMNHYIRADSNVAVLLVVLKSLARKVSTSQSVLAQLQIIDRSLPELVEARANIN	119	turnip mosaic virus
AB701702	ILAFYNSGSLEYLMNHYIGADSNVAVLLVVLKSLAQKVSTSQSVLAQLQIIERSLPELVEAKANIN	119	turnip mosaic virus
AB701710	ILAFYNSGSLEYLMNHYIGADSNVAVLLVVLKSLAQKVSTSQSVLAQLQIIERSLPELVEAKANIN	119	turnip mosaic virus
AB701736	ILAFYNSGSLEYLMNHYIGADSNVAVLLVVLKSLAQKVSTSQSVLAQLQIIERSLPELVEAKANIN	119	turnip mosaic virus
AB701708	ILAFYNSGSLEYLMNHYIRADSNVAVLLVVLKSLAQKVSTSQSVLAQLQIIERSLPELVEAKANIN	119	turnip mosaic virus
AB701703	ILAFYNSGSLEHLMNHYIRADSNVAVLLVVLKSLARKVSTSQSVLAQLQIIERSLPELVEAKANIN	119	turnip mosaic virus
AB701699	ILAFYNSGSLEHLMNHYIRADSNVAVLLVVLKSLAKKVSTSQSVLAQLQIIERSLPELIEAKANIN	119	turnip mosaic virus
AB701729	ILAFYNSGSLEHLMNYYIRADSNVAVLLVVLKSLAKKVSTSQSVMAQLQIIERSLPELIEAKANIN	119	turnip mosaic virus
AB701716	ILAFYNSGSLEHLMNHYISADSNVAVLLVVLKSLAQKVSTSQSVLAQLQIIERSLPELIEAKANVK	119	turnip mosaic virus
AB701717	ILAFYNSGSLEHLMNHYISADSNVAVLLVVLKSLAQKVSTSQSVLAQLQIIERSLPELIEAKANVK	119	turnip mosaic virus
AB701739	ILAFYNSGSLEYLMNHYIRADSNVAVLLVVLKSLAKKVSTSQSVLAQLQIIDRSLPELVEAKANIN	119	turnip mosaic virus
AB701700	ILAFYNSGSLEYLMNHYIRADSNVAVLLVVLKSLAKKVSTSQSVLAQLQIIDRSLPELVEAKANIN	119	turnip mosaic virus
AB701727	ILAFYNSGSLEYLMNHYIRVDSNVAVLLVVLKSLAKKVSTSQSVLAQLQIIERSLPELIEAKANVN	119	turnip mosaic virus
AB701742	ILAFYNSGSLEYLMNHYIRVDSNVAVLLVVLKSLAKKVSTSQSVLAQLQIIERSLPELIEAKANVN	119	turnip mosaic virus
AB701711	ILAFYNSGSLEYLMNHYIRVDSNVAVLLVVLKSLAKKVSTSQSVLAQLQIIERSLPELIEAKTNVN	119	turnip mosaic virus
AB362512	ILAFYNSGSLEYLMNHYIRVDSNVAVLLVVLKSLAKKVSTSQSVLAQLQIIERSLPELIEAKANVN	119	turnip mosaic virus
AB701712	ILAFYNSGSLEYLMNHYIRVDSNVAVLLVVLKSLAKKVSTSQSVLAQLQIIERSLPELIEAKTNVN	119	turnip mosaic virus
AB701718	ILAFYNSGSLEYLMNHYIRVDSNVAVLLVVLKSLAKKVSTSQSVLAQLQIIERSLPELIEAKTNVN	119	turnip mosaic virus
AB701715	ILAFYNSGSLEYLMNHYIRVDSNVAVLLVVLKSLAKKVSTSQSVLAQLQIIERSLPELIEAKHNVN	119	turnip mosaic virus
AB701713	ILAFYNSGSLEYLMNHYIRVDSNVAVLLVVLKSLAKKVSTSQSVLAQLQIIERSLPELIEAKTNVD	119	turnip mosaic virus
AB194790	ILAFYNSGSLEYLMNHYIRVDSNVAVLLVVLKSLAKKVSTSQSVLAQLQIIERSLPELIEAKANVN	119	turnip mosaic virus
AB194785	ILAFYNSGSLEYLMNHYIRVDSNVAVLLVVLKSLAKKVSTSQSVLAQLQIIERSLPELIEAKANVN	119	turnip mosaic virus
AB701730	ILAFYNSGSLEYLMNHYIRADSNVAVLLVVLKSLARKVSTSQSVLAQLQIIERSLPELIEAKANVN	119	turnip mosaic virus
AB701733	ILAFYNSGSLEYLMNHYIRVDSNVAVLLVVLKSLAKKVSTSQSVLAQLRIIERSLPELIEAKANVN	119	turnip mosaic virus
AB701709	ILAFYNSGSLEYLMNHYIRVDSNVAVLLVVLKSLAKKVSTSQSVLAQLQIIERSLPELVEARANVN	119	turnip mosaic virus
AB701704	ILAFYNSGSLEYLMNHYIRADSNVAVLLVVLKSLAKKVSTSQSVLAQLQIIERSLPELVEARANIN	119	turnip mosaic virus
AB362513	ILAFYNSGSLEYLMNHYIRADSNVAVLLVVLKSLAKKVSTSQSVLAQLQIIDRSLPELVEARANIA	119	turnip mosaic virus
AB701722	ILAFYNSGSLENLMNHYIRADSNVAVLLVVLKSLAKKVSTSQSVLAQLQTIERSLPEIVEARANIT	119	turnip mosaic virus
AB701723	ILAFYNSGSLEHLMNHYIRADSNVAVLLVVLKSLAKKVSTSQSVLAQLQVIERSLPEIVEARANIT	119	turnip mosaic virus
AB701724	ILAFYNSGSLEYLMNHYIRADSNVAVLLVVLKSLAKKVSTSQSVLAQLQTIERSLPEIVEARANIT	119	turnip mosaic virus
AB440238	ILAFYNSGSLEYLMNHYIRVDSNVAVLLVVLKSLAKKVSVSQSVLAQLQIIERSLPELVEARANIA	119	turnip mosaic virus
AB440239	ILAFYNSGSLEYLMNHYIRVDSNVAVLLVVLKSLAKKVSVSQSVLAQLQIIERSLPELVEARANIA	119	turnip mosaic virus
AB701734	ILAFYNSGSLEYLMNHYIRADSNVAVLLVVLKSLANKVSVSQSVLAQLQIIERSLPEIVEARANIA	119	turnip mosaic virus
AB701698	ILAFYNSGSLEHLMNHYIRVDSNVAVLLVVLKSLAKKVSTSRSVMAQLQIIERSLPELVEARANIN	119	turnip mosaic virus
AB701690	ILAFYNSGSLECLMDYYIQADCNIAVLLVVLKSLAQKISTSQSVLAQLQIIDRSLPELVEARANVV	119	turnip mosaic virus
AB701692	ILAFYNSGSLECLMDYYIQADCNIAVLLVVLKSLAQKISTSQSVLAQLQIIDRSLPELVEARANVV	119	turnip mosaic virus
AB701693	ILAFYNSGSLECLMDYYIQADCNIAVLLVVLKSLAQKISTSQSVLAQLQIIDRSLPELVEARANVV	119	turnip mosaic virus
AB818538	LTSLFTTGSLYQATLSLIPEDTSARHLVCLLTSLAGRVSRLEDLHDQVNIIEENLGAFLEILSVGD	113	Habenaria mosaic virus
NC_005778	LNEFYTNGSLYEAMEFVGHTQMPVRVAVTKILELAQKVNRAESIYEQASLISNHLSELHGIIDQMP	110	chilli veinal mottle virus
AJ972878	LNEFYTNGSLHEAMEFVGHTQMPVRVVVTKILELAQKVNRAESIYDQASLISNHLSELHEIIDQMP	110	chilli veinal mottle virus
NC_014898	LIALFNSGSLDKMADMYVRSDMDLSAVASSLTLLAHKVSIAKTLEFQLDLMQRQAEVLHNLISQSN	120	lupine mosaic virus

Supplementary Figure 8 (part 2/3)

Accession	P3N P3N		ALT ALT		Viral species
AB576053	KPY-ASYMMAVKTLERMKARTESDHTLNDLGFSVLR-QATPHLVEK	156	FISRNWSKLGKS--------	168	plum pox virus
AB576046	KPY-ASYMMAVKTLERMKARTESDHTLNDLGFSVLR-QATPHLVEK	156	VISRNWSKLGKS--------	168	plum pox virus
AB576072	KPY-ASYMMAVKTLERMKARTESDHTLNDLGFSVLR-QATPHLVEK	156	VISRNWSKLGKS--------	168	plum pox virus
AB576071	KPY-ASYMMAVKTLERMKARTESDHTLNDLGFSVLR-QATPHLVEK	156	IISRNWSKLGKS--------	168	plum pox virus
AB545926	KPY-ASYMMAVKTLERMKARTESDHTLNDLGFSVLR-QATPHLVEK	156	VISRNWSKLGKS--------	168	plum pox virus
AB576045	KPY-ASYMMAVKTLERMKARTESDHTLNDLGFSVLR-QATPHLVEK	156	IISRNWSKLGKS--------	168	plum pox virus
AB576060	KPY-ASYMMAVKTLERMKARTESDHTLNDLGFSVLR-QATPHLVEK	156	VISRNWSKLGKS--------	168	plum pox virus
NC_001445	QPY-ASYMMAVKTLERMKARTESDHTLNDLGFSVLR-QATPHLVEK	156	VISRNWSKLGKS--------	168	plum pox virus
NC_004752	KPN-TSYMLAVNFLMRIKSRLDTDESLQLAGFRSLM-YTSCHHVEK	156	TMWLFWSWSGKN--------	168	yam mosaic virus
AB711144	NLN-LTYPTALMVLQVVKNRNECDDTLFKAGFSHYN-MSVVQIMEK	172	II------------------	174	potato virus y
AB714135	NLN-LTYPTALMVLQVVKNRNECDETLFKAGFSHYN-MSVVQIMEK	172	II------------------	174	potato virus y
AB711153	NLH-LTYPTALMVLQVVKNRNECDDTLFKAGFPSYN-TSVVQIMEK	172	II------------------	174	potato virus y
NC_001616	NLH-LTYPTALMVLQVVKNRNECDDTLFKAGFPSYN-TSVVQIMEK	172	II------------------	174	potato virus y
AM113988	NLH-LTYPTALMVLQVVKNRNECDDTLFKAGFPSYN-TSVVQIMEK	172	II------------------	174	potato virus y
AB711143	NLH-LTYPTALMVLQVVKNRNECDDTLFKAGFPSYN-TSVVQIMEK	172	II------------------	174	potato virus y
AB711151	NLH-LTYPTALMVLQVVKNRNECDDTLFKAGFPSYN-TSVLQIMEK	172	II------------------	174	potato virus y
AB711149	NLH-LTYPTALMVLQVVKNRNECDDTLFKAGFPSYN-TSVVQIMEK	172	II------------------	174	potato virus y
AB711147	NLH-LTYPTALMVLQVVKNRNECDDTLFKAGFPSYN-TSVLQIMEK	172	II------------------	174	potato virus y
AB711145	NLH-LTYPTALMVLQVVKNRNECDDTLFKAGFPSYN-TSVVQIMEK	172	II------------------	174	potato virus y
AB711150	NLH-LTYPTALMVLQVVKNRSECDDTLFKAGFPSYN-TSVVQIMEK	172	II------------------	174	potato virus y
FJ214726	NLY-LTYPTALLILEVVKNRNECDDTLFKAGFLN-S-ASVVQIMEK	171	II------------------	173	potato virus y
AB126033	RVS-FAYQSALTLLTRMRDQAKANSELISGGFNEYD-QDLAWTLEK	168	IIKASYTTNGKN--------	180	pepper mottle virus
AF501591	RVS-FAYQSALTLLTRMRDQAKANSELISGGFNEYD-QDLAWTLEK	168	IIKASYTTNGKN--------	180	pepper mottle virus
NC_001517	RVS-FAYQSALTLLTRMRDQAKANSELISGGFNEYD-QDLAWTLEK	168	IIKASYTTNGKN--------	180	pepper mottle virus
AB570195	ATQ-HSVSLVHTFLMQLEEKRHTDVSLVGSGFANLS-ATTHELMEK	154	IIWRSWMRPGEI--------	166	lily mottle virus
AM048875	ATQ-HSVSLVHTFLMQLEEKRHTDVSLVGSGFANLS-ATTHELMEK	154	IIWRSWMRPGKI--------	166	lily mottle virus
JN127341	ATQ-HSVSLVHTFLMQLEEKRHTDVSLVGSGFANLC-ASTHELMEK	154	IIWRSWMRHGTH--------	166	lily mottle virus
NC_003492	QPM-HSIHSANVFLMNLNESRETDKTIDELGFYSFK-KSSRILMEK	156	P-------------------	157	bean yellow mosaic virus
AB079888	QPM-HSIHSANVFLMNLNESRETDKTIDELGFYSFK-KSSRILMEK	156	P-------------------	157	bean yellow mosaic virus
AB079886	LPM-HSIHSANVFLMNMSESRETDKTIDELGFYSFK-KSSRILMEK	156	P-------------------	157	bean yellow mosaic virus
**AB011819**	KPM-HSINTANIFLMNLEEGRSTDRTIDELGFHSLK-KSSQVLMEK	156	SGQRI---------------	161	**clover yellow vein virus**
NC_007913	RTM-MSYRMALDLIEVTRNKADSNRTLLEHGYSIFV-KSTYEMLEK	156	IISPS---------------	161	konjac mosaic virus
AJ885005	FPC-KSIDTAICALTVMDEEHKSDQALRDNGFYAHL-LNSSEILEK	156	ATWKI---------------	161	thunberg fritillary virus
AB088221	MTS-HSYQPALLQLQVMANRRDMNSTLDLAGFSILQSEDSMYWMEK	157	VISWN---------------	162	papaya leaf distortion mosaic virus
NC_001768	QKK-KSYTHATRFLLMISEENEMDDPVLNAGYRVLE-ASSHEIMEK	156	PISHC---------------	161	tobacco vein mottling virus
AJ131401	IPN-VSYFQAIQYLTMLAASKEVDAEVRVTGYYTFK-LQTSELLEK	156	LT------------------	158	potato virus A
NC_004039	IPN-VSYFQAIQYLTMLAASKEVDAEVRVTGYYTFK-LQTSELLEK	156	LT------------------	158	potato virus A
AJ131400	IPN-VSYFQAIQYLTMLAASKEVDAEVRVTGYYTFK-LQTSELLEK	156	LT------------------	158	potato virus A
AJ131402	IPN-VSYFQGIQYLTMLAASKEVDAEVRVTGYYTFK-LQTSELLEK	156	LT------------------	158	potato virus A
AJ131403	IPN-ISYFQAIQYLTMLAASKEADAEVRVTGYYTFK-LQASELLEK	156	LT------------------	158	potato virus A
NC_007728	SQS-HSYKPALDLLSQFLERDVTNKQLSDNGFVDIN-ENLYVEMEK	153	SM------------------	155	east Asian passiflora virus
AB604610	THS-HSYRPALGMLTQFLERDLTNKQLSDNGFIDLH-ENLFIEVEK	153	SLCKG---------------	158	east Asian passiflora virus
DQ399708	QSA-HSYKTAKDLLAIYIERRASNNQLIENGFVDIN-DQLYITHEK	153	SMWIV---------------	158	watermelon mosaic virus
AB218280	QSA-QSYKTAKDLLAIYIERRASNNQLIENGFVDIN-DQLYVTHEK	153	SMWIV---------------	158	watermelon mosaic virus
KC292915	QSA-HSYKTAKDLLAMYIERKASNNQLIENGFSDMN-DQLYVMHEK	153	FM------------------	155	watermelon mosaic virus
AY294044	QAP-HSYKTAKDLLTMYIERKASNNQLVENGFVDMN-DKLYMAYEK	153	STQIA---------------	158	soybean mosaic virus
D00507	QAP-HSYKTAKDLLTMYIERKASNNQLVENGFVDMN-DKLYMAYEK	153	STQIA---------------	158	soybean mosaic virus
AY294045	QAP-HSYKTAKDLLPMYIERKASNNQLVENGFVDMN-DKLYMAYEK	153	STQIA---------------	158	soybean mosaic virus
AY216010	QAS-HSYKTAKDLLTMYIERKASNNQLVENGFVDMN-DKLYMAYEK	153	STQIA---------------	158	soybean mosaic virus
AY216987	QAS-HSYKTAKDLLTMYIERKASNNQLVENGFVDMN-DKLYVAYEK	153	STQIA---------------	158	soybean mosaic virus
AY278998	DNQ-RAYVPALDLLTIQVEREFSNKELKTNGYPDLQ-QTLFDMREK	153	FMQSNCTIHGKS--------	165	zucchini yellow mosaic virus
AB188115	DNQ-RAYVPALDLLTIQVEREFSNKELKTNGYPDLQ-QTLFDMREK	153	CMQSNCTIHGKS--------	165	zucchini yellow mosaic virus
AY278999	DNQ-RAYVPALDLLTIQVEREFSNKELKTNGYPDLQ-QTLFDMREK	153	FMQSNCTIHGRS--------	165	zucchini yellow mosaic virus
AY279000	DNQ-RAYVPALDLLTIQVEREFSNKELKTNGYPDLQ-QTLFDMREK	153	FMQSNCTIHGKS--------	165	zucchini yellow mosaic virus
AF014811	DNQ-RAYAPALDLLTIQVEREFSNKELKTNGYPDLH-QTLHDMREK	153	CMRSSYTIHGKS--------	165	zucchini yellow mosaic virus
EU410442	KPW-TSYDRAVELMTIMENSELANETLDKHGFASLE-PKLKAAVEK	150	LTS-----------------	153	Algerian watermelon mosaic virus
EF579955	KPW-VTYDKALELLNVFENTMLTNESLETLGYRTIE-PKLKVAVEK	155	STRSAYNKHGKS--------	167	Moroccan watermelon mosaic virus
AJ278405	HVD-PAKRLLWSHLEAMTTRSEMNKELIAEGYALYD-ERLYTLMEK	155	VM------------------	157	sugarcane mosaic virus
JX237862	HVD-PAKRLLWSHLEAMTTRSEMNKELIAEGYALYD-ERLYTLMEK	155	VM------------------	157	sugarcane mosaic virus
AJ297628	HVD-PAKRLLWSHLEAMTTRSEMNKELIAEGYALYD-ERLYTLMEK	155	IM------------------	157	sugarcane mosaic virus
U57358	HVD-PAKRLLWSHLEAMTTRSEMNRELREEGYALYD-ERLYALMEK	155	VTSIS---------------	160	sorghum mosaic virus
NC_003377	HVD-PAKRLLWSHLEVMSSRAATNKDLLDQGYALYS-DRLYAIIEK	155	PM------------------	157	maize dwarf mosaic virus
AB465608	SRT-PGFALSQKIIRGLAERRESNRVLYEQGHSVASYAASHELLEK	157	FGIIF---------------	162	sweet potato feathery mottle virus
AB439206	SRT-PGFALSQRIIRGLAERRESNRVLYEQGHSVASYAASHELMEK	157	FGIVF---------------	162	sweet potato feathery mottle virus
NC_001841	SRT-PSFALSQKIIRGLAEGRESNRVLYEQGHSIASYAASHELMEK	157	FGIVF---------------	162	sweet potato feathery mottle virus
NC_001671	RTQ-HTYPIVQNQLDIMIERVSADAELLRDGFVVSK-GRVQALIEK	162	TIKTT---------------	167	pea seedrne mosaic virusrus
EU119422	FTS-HSKPLLMSQLEVMCNRRIADGELTGLGFKVYN-QDTYDRIEK	155	NTSTIFEIHGSP--------	167	cocksfoot streak virus
GU214748	AGI-RFRDEVVNVLTMMLAQSEMDSDLTKSGFVDLR-MPLYEMREK	155	YMQAN---------------	160	freesia mosaic virus
AF023848	VEN-PMRNELVSALTMMLARSDVDSTLNKTGFTGFS-DTLLEMREK	155	LLETSSTRYGQS--------	167	peanut mottle virus
NC_000947	QRYPIPYTVVMEMLIILASRSESDAALDAAGFKKFQ-RESIQLMEK	165	II------------------	167	Japanese yam mosaic virus
NC_011541	QRHTIPYTTVMKMLMVLATRAKANLELNIAGYNQIR-LASIEVMEK	164	IICKSWKNNGEG--------	176	narcissus yellow stripe virus
AB701695	GPDGAASQACNKFLGMLIHMSEPNNELANGGYTILR-DHSITILEK	164	VICKSWTSRGAN--------	176	turnip mosaic virus
AB701694	GPDGAASQACNKFLGMLIHMSEPNNELANGGYTILR-DHSITILEK	164	VICKSWTSHGAN--------	176	turnip mosaic virus
AB701737	GSDGAASQACNRFLGMLIHMSEPNEELANGGYTILR-DHSITILEK	164	VICKSWTKHGAN--------	176	turnip mosaic virus
AB701720	GPDSAASQACNKFLGMLIHMSEPNNELASGGYTILR-DHSITILEK	164	AICKSWTKHGAS--------	176	turnip mosaic virus
AB701726	GPDSAASQACNKFLGMLIHMSEPNNELASGGYTILR-DHSITILEK	164	AICKSWTKHGAS--------	176	turnip mosaic virus
AB701705	GPDSAASQACNKFLGMLIHMSEPNNELASGGYTILR-DHSITILEK	164	AICKSWTKHGAS--------	176	turnip mosaic virus
AB701696	GPDGAASQACNKFLGMLIHMSEPNNELADGGYTILR-DHSITILEK	164	AICKSWTKRGAN--------	176	turnip mosaic virus
AB701706	APDNAASQACNRFLGMLIHMSEPNNELADGGYTILR-DHSITILEK	164	AICKSWTKRGAS--------	176	turnip mosaic virus
AB701707	GPDNAASQACNRFLGMLIHMSEPNNELADGGYTILR-DHSITILEK	164	AICKSWTKRGAS--------	176	turnip mosaic virus
AB701721	GSDCAASQACNRFLGMLIHMSEPNNELASGGYTILR-DHSITILEK	164	VICRYWTKRGAN--------	176	turnip mosaic virus
AB701697	EPDSAAAQACNKFLGMLVHMAEPNNELADGGYTILR-DHSITILEK	164	VICKSWTKHGAN--------	176	turnip mosaic virus
AB701725	GPDEAAAQACNRFLGMLIHMAEPNNELADGGYTILR-DHSITILEK	164	VICKSWTKHGAS--------	176	turnip mosaic virus
AB701701	GPDSAAAQACNRFMGMLINMAEPNNELADGGYTILR-DHSIAILEK	164	VICKSWTKHGTS--------	176	turnip mosaic virus
AB701740	GPDNAAAQACNRFMGMLINMAEPNNELADGGYTILR-DHSITILEK	164	VICKSWTKHGTS--------	176	turnip mosaic virus
AB701702	GPDGAASRACNRFMGMLLHMAEPNYELANGGYTTLR-DHSISILEK	164	VICRSWTKHGTS--------	176	turnip mosaic virus
AB701710	GPDGAASRACNRFMGMLLHMAEPNYELANGGYTTLR-DHSISILEK	164	VICRSWTKHGTS--------	176	turnip mosaic virus
AB701736	GPDGAASRACNRFMGMLLHMAEPNYELANGGYTILR-DHSISILEK	164	VICRSWTKHGTS--------	176	turnip mosaic virus
AB701708	EPDGAASRACNRFMGMLLHMAEPNYELANGGYTTLR-DHSISILEK	164	VICRSWTRHGTS--------	176	turnip mosaic virus
AB701703	EPDSAASRACNRFMGMLLHMAEPNYELANGGYASLR-DHSISILEK	164	VICRSWTRHGTS--------	176	turnip mosaic virus
AB701699	GPDDAATRACNRFMGMLLHMAEPNYELANGGYTFLR-DHSISILEK	164	VICRSWTRHGTS--------	176	turnip mosaic virus
AB701729	GPDDAATRACNRFMGMLLHMAEPNYELANGGYTFLR-DHSISILEK	164	VICRSWTKHGTS--------	176	turnip mosaic virus
AB701716	GPDDAATRACNRFMGMLLHMAEPNYELANGGYTFLR-DHSISILEK	164	VICRSWTKHGTN--------	176	turnip mosaic virus
AB701717	GPDDAATRACNKFMGMLLHMAEPNYELANGGYTFLR-DHSISILEK	164	VICRSWTKHGTS--------	176	turnip mosaic virus
AB701739	GPDGAATRASNRFMGMLLHMAEPNYELANGGYTSLR-DHSISILEK	164	VICRSWTRHGTS--------	176	turnip mosaic virus
AB701700	GPDGAATRASNRFMGMLLHMAEPNYELANGGYTSLR-DHSISILEK	164	VICRSWTRHGTS--------	176	turnip mosaic virus
AB701727	GPDDAATRACNRFMGMLLHMAEPNWELADGGYTILR-DHSISILEK	164	VIYKSWTKHGTS--------	176	turnip mosaic virus
AB701742	GPDDAATRACNRFMGMLLHMAEPNWELADGGYTILR-DHSISILEK	164	VIYKSWTKHGTS--------	176	turnip mosaic virus
AB701711	GPDDAATRACNRFMGMLLHMAEPNWELADGGYTILR-DHSISILEK	164	VIYKSWTKHGTS--------	176	turnip mosaic virus
AB362512	GPDDAATRACNRFMGMLLHMAEPNWELADGGYTILR-DHSISILEK	164	VIYKSWTKHGAS--------	176	turnip mosaic virus
AB701712	GPDDAATRACNRFMGMLLHMAEPNWELADGGYTILR-DHSISILEK	164	VICKSWTKHGTS--------	176	turnip mosaic virus
AB701718	GPDDAATRACDRFMGMLLHMAEPNWELADGGYTILR-DHSISILEK	164	VIYKS---------------	169	turnip mosaic virus
AB701715	GPDDAATRACNRFMGMLLHMAEPNWELADGGYTILR-DHSISILEK	164	VIYKSWTKHGTS--------	176	turnip mosaic virus
AB701713	GPDDAATRACNRFMGMLLHMAEPNWELADGGYTILR-DHSISILEK	164	VIYKSWTKHGTS--------	176	turnip mosaic virus
AB194790	GPDDAATRACNRFMGMLLHMAEPDWELADGGYTILR-DHSISILEK	164	VIYKSWTKHGTS--------	176	turnip mosaic virus
AB194785	GPDDAATRACNRFMGMLLHMAEPNWELADGGYTILR-DHSISILEK	164	VIYKSWTKHGTS--------	176	turnip mosaic virus
AB701730	GPDDAATRACNRFMGMLLHMAEPNWELADGGYTILR-DHSISILEK	164	VICKSWTKHGTS--------	176	turnip mosaic virus
AB701733	GPDDAATHACNRFMGMLLHMAEPNWELADGGYTILR-DHSISILEK	164	VIYKSWTKHGTS--------	176	turnip mosaic virus
AB701709	GPDNAAACACNRFMGMLLHMAEPNWELADGGYTILR-DHSISILEK	164	VIYKSWTKHGTS--------	176	turnip mosaic virus
AB701704	RPDDAAARACNRFMGMLLHMAEPNWELADGGYTVLR-DHSISILEK	164	VIYKSWTKHGTS--------	176	turnip mosaic virus
AB362513	GADEAAAQACNRFMGMLIHMAEPNNELANGGFTILR-DHSISILEK	164	VICRSWTKRGAS--------	176	turnip mosaic virus
AB701722	GPEGAASQACNRFLGMLLHRSEPNDELLNGGYTTLR-DHSVAILEK	164	TICKSWTKRGAN--------	176	turnip mosaic virus
AB701723	SPEGAASQACNRFLGMLLHRSEPNDELLDGGYTILR-DHSIAILEK	164	TICKSWTKHGAN--------	176	turnip mosaic virus
AB701724	GPEGAASQACNRFLGMLLHRSEPNDELLDGGYTILR-DHSIAILEK	164	TICKSWTKHGAN--------	176	turnip mosaic virus
AB440238	ESDGAAAQACNRFLGMLIHRSEPNNELLDGGYTTLR-DHSIAILEK	164	AICKSWTKRGAN--------	176	turnip mosaic virus
AB440239	EPDRAAAQACNRFLGMLIHMSEPNNELLDGGYTTLR-DHSIAILEK	164	AICKSWTKRGAN--------	176	turnip mosaic virus
AB701734	GSDVAASQACNRFLGMLIHMSEPNDELVDGGYTILR-DHSIAILEK	164	AICKSWTKRGAS--------	176	turnip mosaic virus
AB701698	APDDAAAKACNRFMGMLLHMAEPNNELADGGYTILR-DHSISILEK	164	VICRSWTNHGAN--------	176	turnip mosaic virus
AB701690	GPDVAATIACNKFMAMLLHMAEPNNELAGGGYTILR-SHSLDILEK	164	AICKSWTRLGKN--------	176	turnip mosaic virus
AB701692	GPDVAATIACNKFMAMLLHMAEPNNELAGGGYTILR-SHSLDILEK	164	AICKSWTKHGKN--------	176	turnip mosaic virus
AB701693	GPDVAATIACNKFMAMLLHMAEPNNELAGGGYTILR-SHSLDILEK	164	AICKSWTKLGKN--------	176	turnip mosaic virus
AB818538	RCS-YARAFMQRTIEARLESISADEELDASGFRTLR-WKSVRVLEK	157	FTQRIWRPHGENCNLWKSVI	177	Habenaria mosaic virus
NC_005778	CRS-ASICCVLEFLLTSQLDTECDGELNAIGFRTLK-HKSLHIMEK	154	STRRI---------------	159	chilli veinal mottle virus
AJ972878	CRS-ASICHVLEFLITSQLDTECDDELNAVGFRTLK-HKSLHIMEK	154	STRRI---------------	159	chilli veinal mottle virus
NC_014898	NHD-VSRVVILELLQVMMERNETDSTLLSAGFYMLK----------	155	THQA----------------	158	lupine mosaic virus

Supplementary Figure 8 (part 3/3)

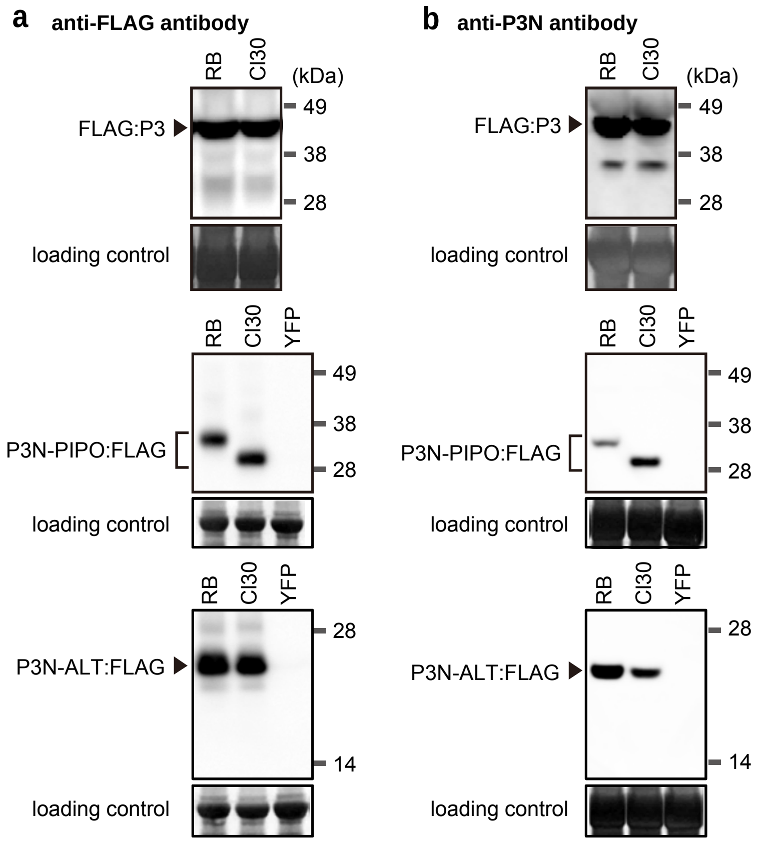

Supplementary Figure 9. Western blotting to confirm specific binding of the anti-P3N peptide antibody to the FLAG-tagged P3 products of Cl30, P3, P3N-PIPO and P3N-ALT. RNAs that produce FLAG:P3, P3N-PIPO:FLAG and P3N-ALT proteins (Supplementary Methods) were transiently expressed in *N. benthamiana* leaves by agroinfiltration, as in Fig. 2. The products were detected by western blotting using an anti-FLAG monoclonal antibody (**a**) or the anti-P3N peptide polyclonal antibody (**b**). Loading controls are the part of the PVDF membranes covering the RubisCO large subunit band that were stained with Amido Black after western transfer. The membranes were stained with 0.1% Amido Black in 45% methanol and 10% acetic acid followed by destaining in 90% methanol and 2% acetic acid.

Supplementary Table 1. Occurrence of G_0A_{6+} and $G_{1\text{-}2}A_{6+}$ motifs in GenBank/EMBL/DDBJ accessions.

Family	Genus	number of accessions			% found	
		total[a]	G_0A_{6+}[b]	$G_{1\text{-}2}A_{6+}$[b]	G_0A_{6+}	$G_{1\text{-}2}A_{6+}$
Alphaflexiviridae		**132**	**86**	**20**	**65.2**	**15.2**
	Potexvirus	132	86	20	65.2	15.2
Alphatetraviridae		**1**	**0**	**0**	**0.0**	**0.0**
	Betatetravirus	1	0	0	0.0	0.0
Arteriviridae		**465**	**379**	**291**	**81.5**	**62.6**
	Arterivirus	465	379	291	81.5	62.6
Astroviridae		**14**	**14**	**14**	**100.0**	**100.0**
	Avastrovirus	14	14	14	100.0	100.0
Bornaviridae		**12**	**4**	**0**	**33.3**	**0.0**
	Bornavirus	12	4	0	33.3	0.0
Betaflexiviridae		**48**	**41**	**14**	**85.4**	**29.2**
	Capillovirus	17	17	7	100.0	41.2
	Carlavirus	2	1	0	50.0	0.0
	Foveavirus	29	23	7	79.3	24.1
Betaflexiviridae		**45**	**35**	**9**	**77.8**	**20.0**
	Trichovirus	26	26	8	100.0	30.8
	Vitivirus	19	9	1	47.4	5.3
Caliciviridae		**81**	**23**	**6**	**28.4**	**7.4**
	Lagovirus	49	22	6	44.9	12.2
	Norovirus	22	0	0	0.0	0.0
	Sapovirus	10	1	0	10.0	0.0
Closteroviridae		**118**	**72**	**35**	**61.0**	**29.7**
	Closterovirus	75	71	34	94.7	45.3
	Vesivirus	43	1	1	2.3	2.3
Coronaviridae		**535**	**442**	**212**	**82.6**	**39.6**
	Alphacoronavirus	191	149	18	78.0	9.4
	Betacoronavirus	336	287	194	85.4	57.7
	Bafinivirus	1	1	0	100.0	0.0
	Deltacoronavirus	4	2	0	50.0	0.0
	Gammacoronavirus	1	1	0	100.0	0.0
	Torovirus	2	2	0	100.0	0.0
Filoviridae		**162**	**162**	**47**	**100.0**	**29.0**
	Ebolavirus	162	162	47	100.0	29.0
Flaviviridae		**5,990**	**5,351**	**2,138**	**89.3**	**35.7**
	Flavivirus	4,765	4,761	1,873	99.9	39.3
	Hepacivirus	1,035	400	139	38.6	13.4
	Pestivirus	190	190	126	100.0	66.3
Hepeviridae		**35**	**0**	**0**	**0.0**	**0.0**
	Hepevirus	35	0	0	0.0	0.0
Hypoviridae		**7**	**2**	**2**	**28.6**	**28.6**
	Hypovirus	7	2	2	28.6	28.6
Luteoviridae		**215**	**16**	**3**	**7.4**	**1.4**
	Enamovirus	3	0	0	0.0	0.0
	Luteovirus	101	13	2	12.9	2.0
	Polerovirus	111	3	1	2.7	0.9
Narnaviridae		**12**	**2**	**1**	**16.7**	**8.3**
	Mitovirus	9	2	1	22.2	11.1
	Narnavirus	3	0	0	0.0	0.0
Paramyxoviridae		**1,269**	**1,244**	**42**	**98.0**	**3.3**
	Avulavirus	324	303	25	93.5	7.7
	Morbillivirus	84	81	6	96.4	7.1
	Pneumovirus	593	592	3	99.8	0.5
	Respirovirus	190	190	5	100.0	2.6
	Rubulavirus	78	78	3	100.0	3.8
Picornaviridae		**1,135**	**415**	**143**	**36.6**	**12.6**
	Aphthovirus	374	37	13	9.9	3.5
	Cardiovirus	43	5	1	11.6	2.3
	Enterovirus	436	192	45	44.0	10.3
	Erbovirus	2	2	2	100.0	100.0
	Hepatovirus	153	151	77	98.7	50.3

	Kobuvirus	33	2	1	6.1	3.0
	Parechovirus	46	24	3	52.2	6.5
	Senecavirus	3	0	0	0.0	0.0
	Teschovirus	45	2	1	4.4	2.2
Potyviridae		**116**	**105**	**86**	**90.5**	**74.1**
	Bymovirus	64	53	38	82.8	59.4
	Ipomovirus	3	3	2	100.0	66.7
	Potyvirus	46	46	43	100.0	93.5
	Rymovirus	2	2	2	100.0	100.0
	Tritimovirus	1	1	1	100.0	100.0
Retroviridae		**2,485**	**2,423**	**1,862**	**97.5**	**74.9**
	Alpharetrovirus	104	93	92	89.4	88.5
	Betaretrovirus	126	109	59	86.5	46.8
	Deltaretrovirus	128	127	21	99.2	16.4
	Epsilonretrovirus	5	5	1	100.0	20.0
	Gammaretrovirus	28	10	1	35.7	3.6
	Lentivirus	2,059	2,045	1,671	99.3	81.2
	Spumavirus	35	34	17	97.1	48.6
Rhabdoviridae		**408**	**298**	**58**	**73.0**	**14.2**
	Cytorhabdovirus	4	3	1	75.0	25.0
	Ephemerovirus	7	7	4	100.0	57.1
	Lyssavirus	295	190	21	64.4	7.1
	Novirhabdovirus	35	33	12	94.3	34.3
	Nucleorhabdovirus	12	10	4	83.3	33.3
	Sigmavirus	1	1	0	100.0	0.0
	Tibrovirus	2	2	0	100.0	0.0
	Tupavirus	1	1	0	100.0	0.0
	Vesiculovirus	51	51	16	100.0	31.4
Secoviridae		**8**	**4**	**4**	**50.0**	**50.0**
	Sequivirus	2	2	2	100.0	100.0
	Waikavirus	6	2	2	33.3	33.3
Togaviridae		**505**	**372**	**52**	**73.7**	**10.3**
	Alphavirus	456	372	52	81.6	11.4
	Rubivirus	49	0	0	0.0	0.0
Tombusviridae		**132**	**5**	**1**	**3.8**	**0.8**
	Alphanecrovirus	10	0	0	0.0	0.0
	Aureusvirus	7	0	0	0.0	0.0
	Avenavirus	1	0	0	0.0	0.0
	Betanecrovirus	20	0	0	0.0	0.0
	Carmovirus	44	2	1	4.5	2.3
	Dianthovirus	9	2	0	22.2	0.0
	Gallantivirus	1	0	0	0.0	0.0
	Macanavirus	1	0	0	0.0	0.0
	Machlomovirus	13	0	0	0.0	0.0
	Panicovirus	3	1	0	33.3	0.0
	Tombusvirus	22	0	0	0.0	0.0
	Zeavirus	1	0	0	0.0	0.0
Totiviridae		**15**	**10**	**3**	**66.7**	**20.0**
	Giardiavirus	3	2	0	66.7	0.0
	Leishmaniavirus	6	6	3	100.0	50.0
	Totivirus	6	2	0	33.3	0.0
Tymoviridae		**10**	**3**	**3**	**30.0**	**30.0**
	Marafivirus	10	3	3	30.0	30.0
Virgaviridae		**125**	**115**	**87**	**92.0**	**69.6**
	Tobamovirus	125	115	87	92.0	69.6
unassigned					**0.0**	**0.0**
	Sobemovirus	64	1	0	1.6	0.0
	Umbravirus	5	0	0	0.0	0.0
**Total**		**14,149**	**11,624**	**5,133**	**82.2**	**36.3**

[a] Number of accessions in GenBank/EMBL/DDBJ.

[b] Number of accessions containing the indicated motif at least once.

Supplementary Table 2. Statistical analysis of the indel frequency observed in the amplicon encompassing G_2A_6 motif.

Window	Geno-type	log_e-transformed estimate ± SE[a]		Adjusted	Geno-type	log_e-transformed estimate ± SE[a]		Adjusted
		Virus	Plasmid	*p*-value[b]		Virus	Plasmid	*p*-value[b]
Window size = 3 base								
Indel = +1 base								
29 - 31	CI30	-9.119 ± 0.866	-10.028 ± 1.118	1	RB	-9.259 ± 0.866	-10.212 ± 1.225	1
31 - 33	CI30	-3.889 ± 0.119	-6.446 ± 0.178	8.20E-50*	RB	-3.736 ± 0.119	-7.163 ± 0.241	2.47E-39*
32 - 34	CI30	-3.893 ± 0.119	-6.446 ± 0.178	9.88E-50*	RB	-3.736 ± 0.119	-7.208 ± 0.246	6.29E-39*
33 - 35	CI30	-3.910 ± 0.119	-6.446 ± 0.178	2.29E-49*	RB	-3.748 ± 0.119	-7.208 ± 0.246	1.49E-38*
Indel = -1 base								
2 - 4	CI30	-6.020 ± 0.165	-5.897 ± 0.138	1	RB	-5.863 ± 0.165	-6.180 ± 0.191	1
3 - 5	CI30	-6.000 ± 0.164	-5.897 ± 0.138	1	RB	-5.863 ± 0.164	-6.164 ± 0.189	1
4 - 6	CI30	-6.000 ± 0.164	-5.897 ± 0.138	1	RB	-5.863 ± 0.164	-6.164 ± 0.189	1
5 - 7	CI30	-7.735 ± 0.527	-8.965 ± 0.646	1	RB	-8.991 ± 0.527	-8.345 ± 0.504	1
6 - 8	CI30	-5.096 ± 0.118	-5.679 ± 0.124	1	RB	-5.665 ± 0.118	-5.060 ± 0.115	1
7 - 9	CI30	-5.096 ± 0.118	-5.679 ± 0.124	1	RB	-5.665 ± 0.118	-5.060 ± 0.115	1
8 - 10	CI30	-5.162 ± 0.121	-5.717 ± 0.126	1	RB	-5.702 ± 0.121	-5.093 ± 0.118	1
11 - 13	CI30	-7.160 ± 0.306	-7.519 ± 0.300	1	RB	-7.317 ± 0.306	-7.726 ± 0.373	1
12 - 14	CI30	-6.636 ± 0.233	-6.951 ± 0.225	1	RB	-6.722 ± 0.233	-7.156 ± 0.284	1
13 - 15	CI30	-6.269 ± 0.195	-6.363 ± 0.175	1	RB	-6.376 ± 0.195	-6.577 ± 0.224	1
14 - 16	CI30	-6.000 ± 0.175	-6.193 ± 0.163	1	RB	-6.272 ± 0.175	-5.837 ± 0.177	1
15 - 17	CI30	-5.656 ± 0.155	-5.852 ± 0.146	1	RB	-6.188 ± 0.155	-5.609 ± 0.152	1
16 - 18	CI30	-5.538 ± 0.142	-5.798 ± 0.136	1	RB	-5.939 ± 0.142	-5.471 ± 0.143	1
17 - 19	CI30	-5.288 ± 0.132	-5.830 ± 0.137	1	RB	-5.947 ± 0.132	-5.267 ± 0.127	1
18 - 20	CI30	-5.641 ± 0.170	-6.019 ± 0.167	1	RB	-5.977 ± 0.170	-5.495 ± 0.170	1
19 - 21	CI30	-5.368 ± 0.127	-5.670 ± 0.122	1	RB	-5.618 ± 0.127	-5.321 ± 0.132	1
20 - 22	CI30	-5.378 ± 0.134	-5.921 ± 0.139	1	RB	-5.878 ± 0.134	-5.479 ± 0.137	1
21 - 23	CI30	-5.378 ± 0.134	-5.933 ± 0.139	1	RB	-5.863 ± 0.134	-5.479 ± 0.137	1
22 - 24	CI30	-5.684 ± 0.174	-6.688 ± 0.204	1	RB	-6.656 ± 0.174	-5.748 ± 0.158	1
23 - 25	CI30	-6.132 ± 0.215	-6.355 ± 0.204	1	RB	-6.444 ± 0.215	-6.374 ± 0.230	1
24 - 26	CI30	-6.061 ± 0.198	-6.591 ± 0.204	1	RB	-6.776 ± 0.198	-6.321 ± 0.199	1
25 - 27	CI30	-6.600 ± 0.265	-7.056 ± 0.267	1	RB	-7.416 ± 0.265	-7.113 ± 0.278	1
26 - 28	CI30	-8.834 ± 0.626	-7.713 ± 0.361	1	RB	-7.950 ± 0.626	-9.598 ± 0.913	1
27 - 29	CI30	-9.813 ± 1.414	-8.236 ± 1.080	1	RB	-10.645 ± 1.414	-9.519 ± 1.225	1
28 - 30	CI30	-9.813 ± 1.414	-8.082 ± 1.069	1	RB	-10.645 ± 1.414	-9.519 ± 1.225	1
29 - 31	CI30	-8.141 ± 0.556	-8.560 ± 0.556	1	RB	-8.837 ± 0.556	-8.905 ± 0.646	1
30 - 32	CI30	-8.323 ± 0.586	-9.659 ± 0.802	1	RB	-8.837 ± 0.586	-9.598 ± 0.837	1
31 - 33	CI30	-4.899 ± 0.108	-6.242 ± 0.146	2.43E-16*	RB	-4.942 ± 0.108	-6.486 ± 0.176	7.04E-17*
32 - 34	CI30	-4.933 ± 0.112	-6.259 ± 0.149	1.16E-15*	RB	-4.960 ± 0.112	-6.531 ± 0.181	3.88E-16*
33 - 35	CI30	-4.933 ± 0.111	-6.259 ± 0.148	8.17E-16*	RB	-4.962 ± 0.111	-6.531 ± 0.180	2.65E-16*
40 - 42	CI30	-9.239 ± 0.791	-9.253 ± 0.677	1	RB	-8.703 ± 0.791	-9.598 ± 1.000	1
Window size = 5 base								
Indel = +1 base								
4 - 8	CI30	-9.932 ± 1.118	-10.352 ± 1.118	1	RB	-9.397 ± 1.118	-9.598 ± 1.225	1
17 - 21	CI30	-9.932 ± 1.155	-9.659 ± 0.913	1	RB	-9.684 ± 1.155	-10.291 ± 1.414	1
18 - 22	CI30	-9.932 ± 1.225	-10.352 ± 1.225	1	RB	-10.090 ± 1.225	-8.499 ± 1.080	1
19 - 23	CI30	-9.932 ± 1.414	-10.352 ± 1.414	1	RB	-10.783 ± 1.414	-8.682 ± 1.095	1
20 - 24	CI30	-9.932 ± 1.414	-10.352 ± 1.414	1	RB	-10.783 ± 1.414	-8.682 ± 1.095	1
21 - 25	CI30	-9.932 ± 1.414	-10.352 ± 1.414	1	RB	-10.783 ± 1.414	-8.682 ± 1.095	1
27 - 31	CI30	-9.119 ± 0.866	-10.028 ± 1.118	1	RB	-9.259 ± 0.866	-10.212 ± 1.225	1
28 - 32	CI30	-7.735 ± 0.401	-10.352 ± 1.025	1	RB	-7.787 ± 0.401	-10.291 ± 1.054	1
29 - 33	CI30	-3.889 ± 0.119	-6.428 ± 0.177	9.88E-50*	RB	-3.734 ± 0.119	-7.163 ± 0.242	2.56E-39*
30 - 34	CI30	-3.889 ± 0.119	-6.446 ± 0.178	6.12E-50*	RB	-3.734 ± 0.119	-7.163 ± 0.241	2.30E-39*
31 - 35	CI30	-3.889 ± 0.119	-6.446 ± 0.178	7.43E-50*	RB	-3.735 ± 0.119	-7.163 ± 0.241	2.41E-39*
32 - 36	CI30	-3.893 ± 0.119	-6.446 ± 0.178	9.88E-50*	RB	-3.736 ± 0.119	-7.208 ± 0.246	6.29E-39*
33 - 37	CI30	-3.910 ± 0.119	-6.446 ± 0.178	2.29E-49*	RB	-3.748 ± 0.119	-7.208 ± 0.246	1.49E-38*
Indel = -1 base								
0 - 4	CI30	-6.020 ± 0.165	-5.897 ± 0.138	1	RB	-5.863 ± 0.165	-6.180 ± 0.191	1
1 - 5	CI30	-6.000 ± 0.164	-5.897 ± 0.138	1	RB	-5.863 ± 0.164	-6.164 ± 0.189	1
2 - 6	CI30	-6.000 ± 0.164	-5.897 ± 0.138	1	RB	-5.863 ± 0.164	-6.164 ± 0.189	1
3 - 7	CI30	-5.855 ± 0.155	-5.852 ± 0.135	1	RB	-5.820 ± 0.155	-6.072 ± 0.178	1
4 - 8	CI30	-4.756 ± 0.095	-5.089 ± 0.092	1	RB	-5.066 ± 0.095	-4.774 ± 0.098	1
5 - 9	CI30	-5.088 ± 0.118	-5.679 ± 0.124	1	RB	-5.665 ± 0.118	-5.055 ± 0.115	1
6 - 10	CI30	-5.096 ± 0.118	-5.679 ± 0.124	1	RB	-5.665 ± 0.118	-5.060 ± 0.115	1
7 - 11	CI30	-5.096 ± 0.118	-5.679 ± 0.124	1	RB	-5.665 ± 0.118	-5.060 ± 0.115	1
8 - 12	CI30	-5.162 ± 0.121	-5.717 ± 0.126	1	RB	-5.702 ± 0.121	-5.093 ± 0.118	1
9 - 13	CI30	-7.160 ± 0.306	-7.519 ± 0.300	1	RB	-7.317 ± 0.306	-7.726 ± 0.373	1
10 - 14	CI30	-6.636 ± 0.233	-6.951 ± 0.225	1	RB	-6.722 ± 0.233	-7.156 ± 0.284	1
11 - 15	CI30	-6.269 ± 0.195	-6.363 ± 0.175	1	RB	-6.376 ± 0.195	-6.577 ± 0.224	1
12 - 16	CI30	-5.728 ± 0.152	-5.957 ± 0.143	1	RB	-5.971 ± 0.152	-5.696 ± 0.158	1
13 - 17	CI30	-5.337 ± 0.128	-5.564 ± 0.121	1	RB	-5.727 ± 0.128	-5.416 ± 0.133	1

14 - 18	Cl30	-5.288 ± 0.124	-5.469 ± 0.115	1	RB	-5.607 ± 0.124	-5.267 ± 0.127	1
15 - 19	Cl30	-4.962 ± 0.110	-5.389 ± 0.111	1	RB	-5.531 ± 0.110	-4.862 ± 0.106	1
16 - 20	Cl30	-5.049 ± 0.112	-5.389 ± 0.109	1	RB	-5.480 ± 0.112	-4.944 ± 0.111	1
17 - 21	Cl30	-4.928 ± 0.103	-5.181 ± 0.098	1	RB	-5.218 ± 0.103	-4.944 ± 0.107	1
18 - 22	Cl30	-4.814 ± 0.102	-5.432 ± 0.108	1	RB	-5.358 ± 0.102	-4.798 ± 0.101	1
19 - 23	Cl30	-4.949 ± 0.108	-5.477 ± 0.111	1	RB	-5.431 ± 0.108	-4.949 ± 0.108	1
20 - 24	Cl30	-5.187 ± 0.117	-5.540 ± 0.115	1	RB	-5.500 ± 0.117	-5.267 ± 0.124	1
21 - 25	Cl30	-4.991 ± 0.113	-5.616 ± 0.120	1	RB	-5.618 ± 0.113	-5.132 ± 0.114	1
22 - 26	Cl30	-5.347 ± 0.146	-6.162 ± 0.162	1	RB	-6.272 ± 0.146	-5.520 ± 0.137	1
23 - 27	Cl30	-6.061 ± 0.178	-6.162 ± 0.162	1	RB	-6.272 ± 0.178	-6.321 ± 0.199	1
24 - 28	Cl30	-6.061 ± 0.198	-6.545 ± 0.201	1	RB	-6.776 ± 0.198	-6.321 ± 0.199	1
25 - 29	Cl30	-6.565 ± 0.260	-6.855 ± 0.252	1	RB	-7.382 ± 0.260	-7.033 ± 0.270	1
26 - 30	Cl30	-8.546 ± 0.553	-7.407 ± 0.329	1	RB	-7.893 ± 0.553	-8.905 ± 0.707	1
27 - 31	Cl30	-8.141 ± 0.556	-8.272 ± 0.518	1	RB	-8.837 ± 0.556	-8.905 ± 0.646	1
28 - 32	Cl30	-8.141 ± 0.540	-8.272 ± 0.500	1	RB	-8.703 ± 0.540	-8.905 ± 0.646	1
29 - 33	Cl30	-4.896 ± 0.117	-6.165 ± 0.149	7.78E-14*	RB	-4.945 ± 0.117	-6.444 ± 0.179	1.99E-15*
30 - 34	Cl30	-4.899 ± 0.109	-6.226 ± 0.146	5.08E-16*	RB	-4.943 ± 0.109	-6.486 ± 0.177	8.48E-17*
31 - 35	Cl30	-4.899 ± 0.108	-6.242 ± 0.146	2.43E-16*	RB	-4.942 ± 0.108	-6.486 ± 0.176	7.04E-17*
32 - 36	Cl30	-4.933 ± 0.112	-6.259 ± 0.149	1.16E-15*	RB	-4.960 ± 0.112	-6.531 ± 0.181	3.88E-16*
33 - 37	Cl30	-4.933 ± 0.111	-6.259 ± 0.148	8.17E-16*	RB	-4.962 ± 0.111	-6.531 ± 0.180	2.65E-16*
Window size = 7 base								
Indel = +1 base								
2 - 8	Cl30	-9.932 ± 1.069	-10.352 ± 1.069	1	RB	-8.837 ± 1.069	-9.598 ± 1.225	1
3 - 9	Cl30	-9.932 ± 1.095	-10.352 ± 1.095	1	RB	-9.173 ± 1.095	-9.598 ± 1.225	1
4 - 10	Cl30	-9.932 ± 1.118	-10.352 ± 1.118	1	RB	-9.397 ± 1.118	-9.598 ± 1.225	1
15 - 21	Cl30	-9.932 ± 1.118	-9.659 ± 0.866	1	RB	-9.397 ± 1.118	-9.598 ± 1.225	1
16 - 22	Cl30	-9.932 ± 1.155	-9.659 ± 0.913	1	RB	-9.684 ± 1.155	-8.499 ± 1.080	1
17 - 23	Cl30	-9.932 ± 1.155	-9.253 ± 0.817	1	RB	-9.684 ± 1.155	-8.499 ± 1.080	1
18 - 24	Cl30	-9.932 ± 1.225	-9.659 ± 1.000	1	RB	-10.090 ± 1.225	-8.499 ± 1.080	1
19 - 25	Cl30	-9.932 ± 1.225	-10.352 ± 1.225	1	RB	-10.090 ± 1.225	-8.682 ± 1.095	1
20 - 26	Cl30	-9.932 ± 1.225	-10.352 ± 1.225	1	RB	-10.090 ± 1.225	-8.682 ± 1.095	1
21 - 27	Cl30	-9.932 ± 1.414	-10.352 ± 1.414	1	RB	-10.783 ± 1.414	-8.682 ± 1.095	1
25 - 31	Cl30	-9.119 ± 0.837	-10.028 ± 1.095	1	RB	-9.035 ± 0.837	-10.212 ± 1.225	1
26 - 32	Cl30	-7.735 ± 0.401	-10.352 ± 1.025	1	RB	-7.787 ± 0.401	-10.291 ± 1.054	1
27 - 33	Cl30	-3.889 ± 0.119	-6.428 ± 0.177	9.88E-50*	RB	-3.734 ± 0.119	-7.163 ± 0.242	2.56E-39*
28 - 34	Cl30	-3.889 ± 0.119	-6.428 ± 0.177	9.12E-50*	RB	-3.734 ± 0.119	-7.163 ± 0.241	2.54E-39*
29 - 35	Cl30	-3.889 ± 0.119	-6.428 ± 0.177	9.12E-50*	RB	-3.734 ± 0.119	-7.163 ± 0.241	2.54E-39*
30 - 36	Cl30	-3.889 ± 0.119	-6.446 ± 0.178	6.12E-50*	RB	-3.734 ± 0.119	-7.163 ± 0.241	2.30E-39*
31 - 37	Cl30	-3.889 ± 0.119	-6.446 ± 0.178	7.43E-50*	RB	-3.735 ± 0.119	-7.163 ± 0.241	2.41E-39*
32 - 38	Cl30	-3.893 ± 0.119	-6.446 ± 0.178	9.88E-50*	RB	-3.736 ± 0.119	-7.208 ± 0.246	6.29E-39*
33 - 39	Cl30	-3.906 ± 0.120	-6.447 ± 0.179	4.85E-49*	RB	-3.747 ± 0.120	-7.117 ± 0.238	6.13E-39*
37 - 43	Cl30	-9.239 ± 0.913	-10.352 ± 1.155	1	RB	-9.684 ± 0.913	-9.598 ± 1.000	1
Indel = -1 base								
1 - 7	Cl30	-5.855 ± 0.155	-5.852 ± 0.135	1	RB	-5.820 ± 0.155	-6.072 ± 0.178	1
2 - 8	Cl30	-4.756 ± 0.095	-5.089 ± 0.092	1	RB	-5.066 ± 0.095	-4.774 ± 0.098	1
3 - 9	Cl30	-4.756 ± 0.095	-5.089 ± 0.092	1	RB	-5.066 ± 0.095	-4.774 ± 0.098	1
4 - 10	Cl30	-4.756 ± 0.095	-5.089 ± 0.092	1	RB	-5.066 ± 0.095	-4.774 ± 0.098	1
5 - 11	Cl30	-5.088 ± 0.118	-5.679 ± 0.124	1	RB	-5.665 ± 0.118	-5.055 ± 0.115	1
6 - 12	Cl30	-5.096 ± 0.118	-5.679 ± 0.124	1	RB	-5.665 ± 0.118	-5.060 ± 0.115	1
7 - 13	Cl30	-4.976 ± 0.110	-5.532 ± 0.114	1	RB	-5.490 ± 0.110	-4.993 ± 0.110	1
8 - 14	Cl30	-4.956 ± 0.107	-5.461 ± 0.110	1	RB	-5.394 ± 0.107	-4.973 ± 0.109	1
9 - 15	Cl30	-6.269 ± 0.195	-6.363 ± 0.175	1	RB	-6.376 ± 0.195	-6.577 ± 0.224	1
10 - 16	Cl30	-5.728 ± 0.152	-5.957 ± 0.143	1	RB	-5.971 ± 0.152	-5.696 ± 0.158	1
11 - 17	Cl30	-5.337 ± 0.128	-5.564 ± 0.121	1	RB	-5.727 ± 0.128	-5.416 ± 0.133	1
12 - 18	Cl30	-5.145 ± 0.115	-5.348 ± 0.107	1	RB	-5.441 ± 0.115	-5.185 ± 0.120	1
13 - 19	Cl30	-4.791 ± 0.099	-5.198 ± 0.099	1	RB	-5.265 ± 0.099	-4.766 ± 0.099	1
14 - 20	Cl30	-4.889 ± 0.102	-5.159 ± 0.098	1	RB	-5.258 ± 0.102	-4.819 ± 0.103	1
15 - 21	Cl30	-4.691 ± 0.091	-4.927 ± 0.086	1	RB	-4.996 ± 0.091	-4.635 ± 0.094	1
16 - 22	Cl30	-4.507 ± 0.088	-5.039 ± 0.091	1	RB	-5.073 ± 0.088	-4.483 ± 0.086	1
17 - 23	Cl30	-4.639 ± 0.092	-5.058 ± 0.092	1	RB	-5.089 ± 0.092	-4.674 ± 0.093	1
18 - 24	Cl30	-4.701 ± 0.094	-5.181 ± 0.096	1	RB	-5.117 ± 0.094	-4.685 ± 0.095	1
19 - 25	Cl30	-4.680 ± 0.096	-5.264 ± 0.101	1	RB	-5.265 ± 0.096	-4.730 ± 0.095	1
20 - 26	Cl30	-4.969 ± 0.107	-5.341 ± 0.105	1	RB	-5.362 ± 0.107	-5.121 ± 0.113	1
21 - 27	Cl30	-4.969 ± 0.110	-5.516 ± 0.115	1	RB	-5.525 ± 0.110	-5.121 ± 0.113	1
22 - 28	Cl30	-5.347 ± 0.146	-6.132 ± 0.160	1	RB	-6.272 ± 0.146	-5.520 ± 0.137	1
23 - 29	Cl30	-6.040 ± 0.177	-6.075 ± 0.157	1	RB	-6.261 ± 0.177	-6.284 ± 0.196	1
24 - 30	Cl30	-6.040 ± 0.196	-6.440 ± 0.195	1	RB	-6.758 ± 0.196	-6.284 ± 0.196	1
25 - 31	Cl30	-6.406 ± 0.239	-6.796 ± 0.237	1	RB	-7.199 ± 0.239	-6.959 ± 0.255	1
26 - 32	Cl30	-7.735 ± 0.389	-7.356 ± 0.300	1	RB	-7.564 ± 0.389	-8.499 ± 0.527	1
27 - 33	Cl30	-4.896 ± 0.119	-6.136 ± 0.149	4.47E-13*	RB	-4.946 ± 0.119	-6.444 ± 0.181	3.81E-15*
28 - 34	Cl30	-4.896 ± 0.119	-6.136 ± 0.149	4.47E-13*	RB	-4.946 ± 0.119	-6.444 ± 0.181	3.81E-15*
29 - 35	Cl30	-4.896 ± 0.117	-6.165 ± 0.149	7.78E-14*	RB	-4.945 ± 0.117	-6.444 ± 0.179	1.99E-15*
30 - 36	Cl30	-4.899 ± 0.109	-6.226 ± 0.146	5.08E-16*	RB	-4.943 ± 0.109	-6.486 ± 0.177	8.48E-17*
31 - 37	Cl30	-4.899 ± 0.108	-6.242 ± 0.146	2.43E-16*	RB	-4.942 ± 0.108	-6.486 ± 0.176	7.04E-17*

32 - 38	Cl30	-4.933 ± 0.112	-6.259 ± 0.149	1.16E-15*	RB	-4.960 ± 0.112	-6.531 ± 0.181	3.88E-16*
33 - 39	Cl30	-4.935 ± 0.117	-6.259 ± 0.153	6.14E-15*	RB	-4.960 ± 0.117	-6.466 ± 0.180	6.63E-15*
36 - 42	Cl30	-9.239 ± 0.775	-9.253 ± 0.658	1	RB	-8.480 ± 0.775	-8.682 ± 0.837	1
Window size = 9 base								
Indel = +1 base								
0 - 8	Cl30	-9.932 ± 1.069	-10.352 ± 1.069	1	RB	-8.837 ± 1.069	-9.598 ± 1.225	1
1 - 9	Cl30	-9.932 ± 1.069	-10.352 ± 1.069	1	RB	-8.837 ± 1.069	-9.598 ± 1.225	1
2 - 10	Cl30	-9.932 ± 1.069	-10.352 ± 1.069	1	RB	-8.837 ± 1.069	-9.598 ± 1.225	1
3 - 11	Cl30	-9.932 ± 1.095	-10.352 ± 1.095	1	RB	-9.173 ± 1.095	-9.598 ± 1.225	1
4 - 12	Cl30	-9.932 ± 1.118	-10.352 ± 1.118	1	RB	-9.397 ± 1.118	-9.598 ± 1.225	1
13 - 21	Cl30	-9.932 ± 1.118	-9.659 ± 0.866	1	RB	-9.397 ± 1.118	-9.598 ± 1.225	1
14 - 22	Cl30	-9.932 ± 1.118	-9.659 ± 0.866	1	RB	-9.397 ± 1.118	-8.345 ± 1.069	1
15 - 23	Cl30	-9.932 ± 1.118	-9.253 ± 0.764	1	RB	-9.397 ± 1.118	-8.345 ± 1.069	1
16 - 24	Cl30	-9.932 ± 1.155	-9.253 ± 0.817	1	RB	-9.684 ± 1.155	-8.499 ± 1.080	1
17 - 25	Cl30	-9.932 ± 1.118	-9.253 ± 0.764	1	RB	-9.397 ± 1.118	-8.499 ± 1.080	1
18 - 26	Cl30	-9.932 ± 1.155	-9.659 ± 0.913	1	RB	-9.684 ± 1.155	-8.499 ± 1.080	1
19 - 27	Cl30	-9.932 ± 1.225	-10.352 ± 1.225	1	RB	-10.090 ± 1.225	-8.682 ± 1.095	1
20 - 28	Cl30	-9.932 ± 1.225	-10.352 ± 1.225	1	RB	-10.090 ± 1.225	-8.682 ± 1.095	1
21 - 29	Cl30	-9.932 ± 1.225	-9.659 ± 1.000	1	RB	-10.090 ± 1.225	-8.682 ± 1.095	1
23 - 31	Cl30	-9.239 ± 0.837	-9.659 ± 0.837	1	RB	-9.174 ± 0.837	-10.291 ± 1.225	1
24 - 32	Cl30	-7.735 ± 0.398	-10.352 ± 1.024	1	RB	-7.738 ± 0.398	-10.291 ± 1.054	1
25 - 33	Cl30	-3.889 ± 0.119	-6.428 ± 0.177	9.12E-50*	RB	-3.734 ± 0.119	-7.163 ± 0.241	2.54E-39*
26 - 34	Cl30	-3.889 ± 0.119	-6.428 ± 0.177	9.12E-50*	RB	-3.734 ± 0.119	-7.163 ± 0.241	2.54E-39*
27 - 35	Cl30	-3.889 ± 0.119	-6.428 ± 0.177	9.12E-50*	RB	-3.734 ± 0.119	-7.163 ± 0.241	2.54E-39*
28 - 36	Cl30	-3.889 ± 0.119	-6.428 ± 0.177	9.12E-50*	RB	-3.734 ± 0.119	-7.163 ± 0.241	2.54E-39*
29 - 37	Cl30	-3.889 ± 0.119	-6.428 ± 0.177	9.12E-50*	RB	-3.734 ± 0.119	-7.163 ± 0.241	2.54E-39*
30 - 38	Cl30	-3.889 ± 0.119	-6.446 ± 0.178	6.12E-50*	RB	-3.734 ± 0.119	-7.163 ± 0.241	2.30E-39*
31 - 39	Cl30	-3.885 ± 0.120	-6.447 ± 0.179	1.35E-49*	RB	-3.733 ± 0.120	-7.076 ± 0.234	1.06E-39*
32 - 40	Cl30	-3.889 ± 0.120	-6.447 ± 0.179	2.03E-49*	RB	-3.734 ± 0.120	-7.117 ± 0.238	2.53E-39*
33 - 41	Cl30	-3.906 ± 0.120	-6.447 ± 0.179	4.85E-49*	RB	-3.747 ± 0.120	-7.117 ± 0.238	6.13E-39*
Indel = -1 base								
0 - 8	Cl30	-4.756 ± 0.095	-5.089 ± 0.092	1	RB	-5.066 ± 0.095	-4.774 ± 0.098	1
1 - 9	Cl30	-4.756 ± 0.095	-5.089 ± 0.092	1	RB	-5.066 ± 0.095	-4.774 ± 0.098	1
2 - 10	Cl30	-4.756 ± 0.095	-5.089 ± 0.092	1	RB	-5.066 ± 0.095	-4.774 ± 0.098	1
3 - 11	Cl30	-4.756 ± 0.095	-5.089 ± 0.092	1	RB	-5.066 ± 0.095	-4.774 ± 0.098	1
4 - 12	Cl30	-4.756 ± 0.095	-5.089 ± 0.092	1	RB	-5.066 ± 0.095	-4.774 ± 0.098	1
5 - 13	Cl30	-4.969 ± 0.110	-5.532 ± 0.114	1	RB	-5.490 ± 0.110	-4.988 ± 0.109	1
6 - 14	Cl30	-4.902 ± 0.105	-5.432 ± 0.108	1	RB	-5.367 ± 0.105	-4.944 ± 0.106	1
7 - 15	Cl30	-4.826 ± 0.100	-5.270 ± 0.101	1	RB	-5.265 ± 0.100	-4.862 ± 0.102	1
8 - 16	Cl30	-4.712 ± 0.094	-5.137 ± 0.095	1	RB	-5.134 ± 0.094	-4.656 ± 0.095	1
9 - 17	Cl30	-5.337 ± 0.128	-5.564 ± 0.121	1	RB	-5.727 ± 0.128	-5.416 ± 0.133	1
10 - 18	Cl30	-5.145 ± 0.115	-5.348 ± 0.107	1	RB	-5.441 ± 0.115	-5.185 ± 0.120	1
11 - 19	Cl30	-4.791 ± 0.099	-5.198 ± 0.099	1	RB	-5.265 ± 0.099	-4.766 ± 0.099	1
12 - 20	Cl30	-4.791 ± 0.097	-5.069 ± 0.093	1	RB	-5.137 ± 0.097	-4.766 ± 0.099	1
13 - 21	Cl30	-4.557 ± 0.085	-4.803 ± 0.081	1	RB	-4.832 ± 0.085	-4.558 ± 0.089	1
14 - 22	Cl30	-4.411 ± 0.083	-4.871 ± 0.084	1	RB	-4.919 ± 0.083	-4.402 ± 0.082	1
15 - 23	Cl30	-4.456 ± 0.083	-4.830 ± 0.082	1	RB	-4.891 ± 0.083	-4.430 ± 0.084	1
16 - 24	Cl30	-4.423 ± 0.082	-4.863 ± 0.083	1	RB	-4.886 ± 0.082	-4.399 ± 0.083	1
17 - 25	Cl30	-4.435 ± 0.084	-4.914 ± 0.086	1	RB	-4.969 ± 0.084	-4.504 ± 0.085	1
18 - 26	Cl30	-4.562 ± 0.088	-5.039 ± 0.090	1	RB	-5.021 ± 0.088	-4.601 ± 0.090	1
19 - 27	Cl30	-4.664 ± 0.094	-5.193 ± 0.098	1	RB	-5.199 ± 0.094	-4.723 ± 0.095	1
20 - 28	Cl30	-4.969 ± 0.107	-5.328 ± 0.105	1	RB	-5.362 ± 0.107	-5.121 ± 0.113	1
21 - 29	Cl30	-4.962 ± 0.110	-5.469 ± 0.113	1	RB	-5.520 ± 0.110	-5.109 ± 0.112	1
22 - 30	Cl30	-5.337 ± 0.145	-6.061 ± 0.157	1	RB	-6.261 ± 0.145	-5.504 ± 0.136	1
23 - 31	Cl30	-5.943 ± 0.169	-6.048 ± 0.154	1	RB	-6.198 ± 0.169	-6.248 ± 0.190	1
24 - 32	Cl30	-5.943 ± 0.185	-6.420 ± 0.188	1	RB	-6.640 ± 0.185	-6.248 ± 0.190	1
25 - 33	Cl30	-4.729 ± 0.109	-5.802 ± 0.129	8.22E-11*	RB	-4.858 ± 0.109	-6.032 ± 0.153	4.29E-15*
26 - 34	Cl30	-4.876 ± 0.117	-5.973 ± 0.140	5.39E-12*	RB	-4.898 ± 0.117	-6.402 ± 0.178	2.59E-15*
27 - 35	Cl30	-4.896 ± 0.119	-6.136 ± 0.149	4.47E-13*	RB	-4.946 ± 0.119	-6.444 ± 0.181	3.81E-15*
28 - 36	Cl30	-4.896 ± 0.119	-6.136 ± 0.149	4.47E-13*	RB	-4.946 ± 0.119	-6.444 ± 0.181	3.81E-15*
29 - 37	Cl30	-4.896 ± 0.117	-6.165 ± 0.149	7.78E-14*	RB	-4.945 ± 0.117	-6.444 ± 0.179	1.99E-15*
30 - 38	Cl30	-4.899 ± 0.109	-6.226 ± 0.146	5.08E-16*	RB	-4.943 ± 0.109	-6.486 ± 0.177	8.48E-17*
31 - 39	Cl30	-4.901 ± 0.113	-6.242 ± 0.150	1.33E-15*	RB	-4.940 ± 0.113	-6.423 ± 0.176	1.38E-15*
32 - 40	Cl30	-4.936 ± 0.119	-6.259 ± 0.154	1.03E-14*	RB	-4.957 ± 0.119	-6.466 ± 0.182	1.25E-14*
33 - 41	Cl30	-4.936 ± 0.118	-6.259 ± 0.154	7.98E-15*	RB	-4.959 ± 0.118	-6.466 ± 0.181	9.13E-15*

[a] Indel frequencies were estimated by fitting the negative binominal model to observed counts of the reads with indels obtained by amplicon sequenceing. SE, standard error of estimated mean.

[b] *p*-values for differences between the estimated indel frequencies between inoculated plasmid and systemically propagated virus were obtained by the model fitting and were corrected for multiple tests by Holm's method.

Supplementary Table 3. Primers used in this study.

Name	Sequence (5′–3′)
796	AGCTACTAGTCTAAGCCTTGTACAGCTCGTC
797	AGCTCTCGAGATGGTGAGCAAGGGCGAGGAGCTG
1007	AGCTACTAGTTTATTCCATGACAAACCACTTTG
1009	AGTCCTCGAGATGGGCAAATCATTGACAGG
1010	AGTCCTCGAGATGGGCAACCAATCTGTTGG
1011	AGCTACTAGTCTTGTTGCGACCATTCTCTTC
1012	AGCTACTAGTATGCTTGTTACTGACAATCTG
1013	AGCTACTAGTGTCCTCTCGACTCTTCTCTC
1049	AGCTACTAGTTTACTCCATGACAAACCACTTTGG
1123	AGCTACTAGTAATCCTCTGCCCAAATTTTTTCC
1124	AGCTACTAGTAATCCTCTGCCCAGATTTTTTCC
1125	AGCTACTAGTAGGTTTTTTCCATGAGAATTC
1349	GCATACTAGTAATCCTCTGCCCAAATTTCTCCATGAGTACCTGGCTAGAC
1350	GCATACTAGTAATCCTCTGCCCAGATTTCTCCATGAGTACTTGACTAG
1351	GCATACTAGTAGGTTTCTCCATGAGAATTCGAGTTGAC
3668	GGAGAAAAACCTAGATGGCGGACTTAGATC
3669	GATCTAAGTCCGCCATCTAGGTTTTTCTCC
3879	GGAGATCTCTCTTTGAAAAAGTCTAGTCAAGTACTCA
3880	GGAGATCTCTCTTTGAAAAAGTCTAGCCAGGTACTCA
3881	GGCATATGTTGTTACTGACAATCTGAGTGATGC
3882	GGCATATGTTGTTGCTGACAATCTGAGTGATGC
afs_as	CTAAATCCTCTGCCCAGATTTCTCC
afs_s	GGAGAAATCTGGGCAGAGGATTTAG
BamHI-kzk-GFP-F	TTTGGATCCAACAATGGTGAGCAAGGGCGAG
delPIPO_s	GGAGAAAATCTGGGCTGAGGATTTAG
delPIPO_as	ATGAGTACTTGACTAGACTTTTTC
GFP-SmaI-SacI-R	AAAGAGCTCGCCCGGGTTACTTGTACAGCTCGTCCATGCCG
P3s3	GGAGAAAAATTTAGGCAGAGGATTT
P3as3	AAATCCTCTGCCTAAATTTTTCTCC
sGFP-387-R	GATGCCCTTCAGCTCGATGCGGTTCAC
sGFP6421-R	TCGTTGGGGTCTTTGCTCAGGGCG
sGFP642-stop-F	TAAGCGCGATCACATGGTCCTGCT
TTTf1	GACGAAAAAATTTGCTATGGCACAAGCTGGAGTACAACTACA
TTTf2	GACCGAAAAAATTTGTATGGCACAAGCTGGAGTACAACTACA
TTTf3	GAGAAAAAATTTGCTTATGGCACAAGCTGGAGTACAACTACA

Supplementary Methods

Plasmids used for Supplementary Fig. 5

To construct pSP/GFP-f1 [to prepare *GFP-*G_1A_6*(f1)* RNA], pSP/GFP-f2 [to prepare *GFP-*G_1A_6*(f2)* RNA] and pSP/GFP-f3 [to prepare *GFP-*G_1A_6*(f3)* RNA], a template plasmid pSP/GFPstp was generated. The sGFP(S65T) sequence present in the 35Somega-sGFP(S65T) plasmid[69] was PCR amplified using primers BamHI-kzk-GFP-F and GFP-SmaI-SacI-R (Supplementary Table 3). The PCR product was inserted into pSP64 Poly(A) vector using the *Bam*HI and *Sac*I sites. The resulting plasmid was used as a template for inverse PCR with primers sGFP642-stop-F and sGFP6421-R (Supplementary Table 3) to introduce a stop codon with the +1 reading frame. The PCR product was phosphorylated and circularized to produce pSP-GFPstp. Inverse PCR was further conducted using pSP/GFPstp as a template, reverse primer sGFP-387-R (Supplementary Table 3) and one of the following forward primers; TTTf1 for pSP/GFP-f1; TTTf2 for pSP/GFP-f2; and TTTf3 for pSP/GFP-f3 (Supplementary Table 3). The PCR product was phosphorylated and circularized.

Indel enrichment analysis for Supplementary Fig. 6

Enrichment of indels at each position in the amplicons was analysed using Fisher's exact test. Briefly, a 2 × 2 contingency table comprising counts of reads with or without indels in viral or plasmid samples was prepared for each position and each genotype. The ratios of counts of reads with or without indels for each position were used as null hypotheses and *p*-values for enrichment of indels in viral samples were obtained. Storey's *q*-values were calculated from the *p*-values. These were performed using R software (version 3.1.2) with the qvalue package (version 1.43).

Plasmids used for Supplementary Fig. 9

P3 of RB and Cl30 were amplified with primers 1009/1049 and 1009/1007 (Supplementary Table 3) from $P3^{+P3N\text{-}PIPO}$ and pCl30, respectively. Each fragment was digested with *Xho*I and *Spe*I, and introduced into the pTA/3FLAG-XhSp vector to obtain pTA/FLAG-P3-RB and pTA/FLAG-P3-Cl30, which produce P3 proteins tagged with 3×FLAG at the N-terminus. The pTA/3FLAG-XhSp vector was created by introducing 3×FLAG tag + Gly/Ser linker + *Xho*I + *Spe*I fragment with *Sal*I site at the 5′ terminus (gtcgacATGGACTACAAAGACCATGACGGTGATTATAAAGATCATGACATCGATTACA AGGATCATGATGGGggtggaagtggaggtagtggtggaagtggaggtagtCTCGAGatggccACTAGT) into the cloning site using the *Xho*I and *Spe*I sites of pTA7001.

Supplementary References

68. Sarkisova, T. & Petrzik, K. A new potyvirus identified in Czech Republic. *Acta Virol.* **53**, 143 (2009).

69. Chiu, W. *et al*. Engineered GFP as a vital reporter in plants. *Curr. Biol.* **6**, 325–330 (1996).
